# Exploiting Organometallic Chemistry to Functionalize Small Cuprous Oxide Colloidal Nanocrystals

**DOI:** 10.1021/jacs.3c10892

**Published:** 2024-02-01

**Authors:** Bradley E. Cowie, Kristian L. Mears, Mark S’ari, Ja Kyung Lee, Martha Briceno de Gutierrez, Curran Kalha, Anna Regoutz, Milo S. P. Shaffer, Charlotte K. Williams

**Affiliations:** † Department of Chemistry, University of Oxford, Chemistry Research Laboratory, 12 Mansfield Road, Oxford OX1 3TA, U.K.; ‡ Johnson Matthey, Johnson Matthey, Blounts Court, Sonning Common, Reading RG4 9NH, U.K.; § Department of Chemistry, University College London, 20 Gordon Street, London WC1H 0AJ, U.K.; ∥ Department of Materials, Imperial College London, London SW7 2AZ, U.K.; ⊥ Department of Chemistry, Imperial College London, 82 Wood Lane, London W12 0BZ, U.K.

## Abstract

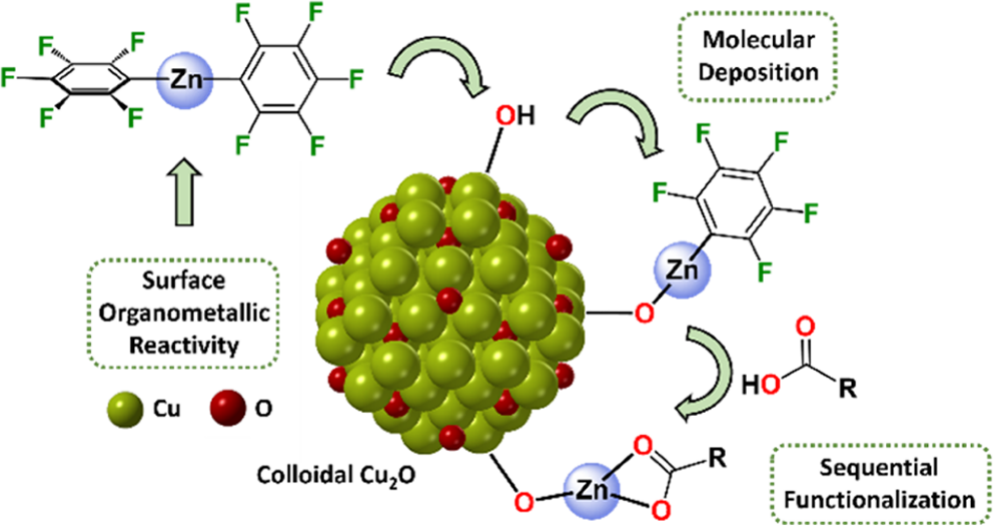

The ligand chemistry of colloidal semiconductor nanocrystals mediates their solubility, band gap, and surface facets. Here, selective organometallic chemistry is used to prepare small, colloidal cuprous oxide nanocrystals and to control their surface chemistry by decorating them with metal complexes. The strategy is demonstrated using small (3–6 nm) cuprous oxide (Cu_2_O) colloidal nanocrystals (NC), soluble in organic solvents. Organometallic complexes are coordinated by reacting the surface Cu–OH bonds with organometallic reagents, M(C_6_F_5_)_2_, M = Zn(II) and Co(II), at room temperature. These reactions do not disrupt the Cu_2_O crystallinity or nanoparticle size; rather, they allow for the selective coordination of a specific metal complex at the surface. Subsequently, the surface-coordinated organometallic complex is reacted with three different carboxylic acids to deliver Cu–O–Zn(O_2_CR’) complexes. Selective nanocrystal surface functionalization is established using spectroscopy (IR, ^19^F NMR), thermal gravimetric analyses (TGA), transmission electron microscopy (TEM, EELS), and X-ray photoelectron spectroscopy (XPS). Photoluminescence efficiency increases dramatically upon organometallic surface functionalization relative to that of the parent Cu_2_O NC, with the effect being most pronounced for Zn(II) decoration. The nanocrystal surfaces are selectively functionalized by both organic ligands and well-defined organometallic complexes; this synthetic strategy may be applicable to many other metal oxides, hydroxides, and semiconductors. In the future, it should allow NC properties to be designed for applications including catalysis, sensing, electronics, and quantum technologies.

## Introduction

Semiconductor nanocrystals (SC-NCs) are important in catalysis, electronics, optics, sensing, and quantum technologies.^1−3^ Colloidal SC-NCs, soluble in polar solvents, are important in formulations, both as “inks” for low-temperature deposition onto electrodes, metals, or glass substrates and to make highly dispersed organic–inorganic hybrid materials.^4,5^ There are many different syntheses of such colloidal SC-NCs; low-temperature routes are particularly attractive, especially for small particles where size-dependent effects can be accessed.^6−9^ The desired high solubility is usually obtained using excess ligands or surfactants during the synthesis.^6,10^ As well as moderating solubility, the ligand chemistry can also influence the SC-NC properties and performances.^6^ However, it can be challenging to control SC-NC surface-ligand chemistry, particularly where different ligands or functional groups need to be introduced onto a single surface.^11^ To help differentiate and understand SC-NC ligand chemistry, several researchers have applied covalent bond classification (CBC) methods, well-known in coordination chemistry.^12−15^ Ligands are classed as neutral donors, such as amines (L-type); single electron donors, such as carboxylates or halides (X-type); or electron-pair acceptors, such as metal(carboxylates) or boranes (Z-type).^12−15^ While organic ligands are perhaps most often applied, Talapin and co-workers discovered SC-NC ligand electronic coupling effects and/or supercrystal lattices using “inorganic” ligands.^16−18^ Many surface-coordinated ligands can exchange with free (pro-)ligands in solution; recent investigations of these exchange reactions focused on CdS, CdSe, or PbS NC.^19−25^ The extent and rate of ligand exchange can be explored using NMR spectroscopies and isothermal calorimetry.^25−27^ Relevant to this work, cuprous oxide SC-NC ligand exchange reactions were investigated using carboxylates (X-type) or amines (L-type).^5,28,29^ In addition to the intentional ligand, metal oxide SC-NCs also likely feature metal hydroxides, water, or solvents as ligands, even when excess surfactant is applied. Alivisatos and co-workers showed that Pb–OH groups were present on colloidal PbS NC, as well as the “added” oleate ligands.^30^ These “unintentional” metal-hydroxide ligands are not usually explicitly considered in SC-NC chemistry yet may affect the exchange reactions and have functions in catalysis. They also provide additional sites for selective SC-NC functionalization, as demonstrated in this work.

Most SC-NC syntheses apply excess ligand(s), but unused reagents are problematic in many applications, including in polymer nanocomposites, catalysis, or theranostics.^10^ In these cases, colloidal stability is best achieved by careful control of the quantity of ligands and their surface coordination chemistry. One attractive synthetic strategy exploits the high reactivity of metal–carbon bonds to hydrolysis or insertions, e.g., Cu– or Zn–C bonds.^9,28,31−35^

Reactions conducted with substoichiometric quantities of nonhydrolyzable X-type ligands, compared to the number of binding sites on the SC-NC surface, yield small (1–5 nm), monodisperse, colloidal Cu_2_O or ZnO SC-NCs.^9,28,29,32,35,36^ By binding strongly chelating ligands to the SC-NC surface intrinsically during the synthesis, it is not necessary to rely on dynamic equilibria with excess ligands in solution. Stable colloids can be prepared with deliberately substoichiometric ligand coverage, leaving “free” surface sites available for application or reaction.^8,28,35,37,38^ This study explores the surface reactivity of putative Cu–OH moieties on colloidal cuprous oxide NC stabilized by long-chain carboxylate ligands. We reasoned that the surface Cu–OH might be reactive toward organometallic complexes, exploiting rapid and irreversible protonolysis reactions. Such reactions could produce colloidal NC functionalized by both organic ligands and organometallic complexes.

The use of reactions between surface metal oxide, M–OH bonds, and organometallic complexes, i.e., M’–C, has been used in heterogeneous catalysis and to attach organometallic complexes to solid-state surfaces.^39^ Surface organometallic chemistry (SOMC) has been used to attach molecular or nanoparticle catalysts to silica, alumina or zirconia supports.^39−43^ For example, Copéret and co-workers used SOMC to install Cu(II) complexes onto intermetallic or oxide surfaces as models for active sites in heterogeneous methane oxidation catalysts.^40,44,45^ The main difference between SOMC and the strategy proposed here is that single nanoparticle transformations must occur in solution and must be compatible with the carboxylate ligands that deliver colloidal stability. Solution reactions of nanoparticles with organometallic reagents also relate to the colloidal atomic layer deposition (c-ALD) process used to coat thin layers of amorphous metal oxides onto colloidal SC-NC.^46,47^ For example, Buonsanti and co-workers reacted metallic or SC nanocrystals with AlMe_3_, followed with O_2_, in repeated cycles, to deposit amorphous alumina layers.^47−49^ Here, by using specific organometallic species, we provide molecular control of the surface chemistry in the colloidal solution.

The solution phase surface organometallic chemistry is targeted to decorate small, colloidal cuprous oxide (Cu_2_O) NCs. Cuprous oxide is a wide band-gap semiconductor (bulk band gap ∼2.17 eV) with a high exciton binding energy.^28^ It is of interest for applications in photocatalytic CO_2_ reduction,^50^ solar cells,^51−54^ and gas sensing.^55^ Controlling surface chemistry provides a means to modulate catalytically active sites, install cocatalysts, or introduce surface dopants to adjust the band gap. While cuprous oxide is a promising photocatalyst, improvements are needed in catalyst lifetime since cuprous oxide is metastable with respect to copper and cupric oxide.^50^

## Results and Discussion

**Figure 1:**
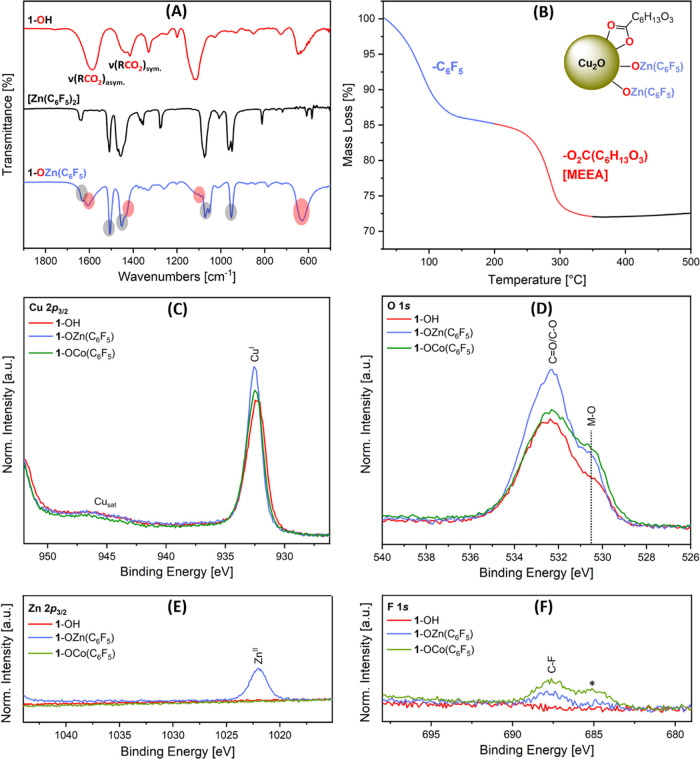
(A) Stacked FT-IR spectra of **1-OH**, [Zn(C_6_F_5_)_2_] and **1-OZn(C**_**6**_**F**_**5**_**)**; red highlights represent the coordinated MEEA ligand, and black highlights represent –C_6_F_5_ groups. (B) TGA thermogram of **1-OZn(C**_**6**_**F**_**5**_**)**. (C) Cu 2p_3/2_, (D) O 1s, (E) Zn 2p_3/2_, and (F) F 1s XP spectra for **1-OH**, **1-OZn(C**_**6**_**F**_**5**_**)**, and **1-OCo(C**_**6**_**F**_**5**_**)**; * represents MF_2_ (M = Zn, Co) attributed to sample degradation during the XPS experiments.

**Scheme 1:**
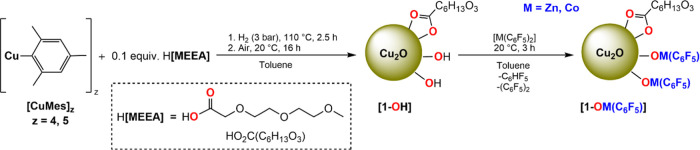
Synthesis of Cu_2_O@(MEEA)(OH) [1-OH] and Cu_2_O@(MEEA){OM(C_6_F_5_)} (M = Zn, 1-OZn(C_6_F_5_)); M = Co, 1-OCo(C_6_F_5_); 1 = Cu_2_O@(MEEA); MEEA = H_3_C(OCH_2_CH_2_)_2_OCH_2_CO_2_^–^ (2-[2-(2-Methoxyethoxy)ethoxy]acetate); and H[MEEA] = H_3_C(OCH_2_CH_2_)_2_OCH_2_CO_2_H (2-[2-(2-Methoxyethoxy)ethoxy]acetic acid)

Colloidal Cu_2_O NCs were synthesized by reacting copper(I)(mesitylene), [CuMes]_*z*_ (*z* = 4, 5), in toluene, with substoichiometric quantities of a carboxylic acid ligand, 2-[2-(2-methoxyethoxy)ethoxy]acetic acid, H[MEEA] (10 mol %).^8,9,28,56^ The solution was hydrogenated (3 bar) at 110 °C to form colloidal Cu@MEEA NCs.^28^ Solutions of the NCs were exposed to air to form colloidal cuprous oxide NCs.^8,9,28^ These products have cubic lattices and are 3–6 nm in size, as confirmed by both XRD (3 nm) and TEM (6.0 ± 1.5 nm; Figures S1–S3); the slightly smaller size observed by XRD compared to TEM may relate to other contributions to peak broadening and the presence of some twinned/multigrain NCs. Thermogravimetric analyses (TGA) and XPS confirmed the carboxylate (MEEA) ligand coordination (Figures 1D, S4, and S23).^28^ IR spectra show resonances at 1588 and 1438/1415 cm^–1^, assigned to asymmetric and symmetric carboxylate stretches (Figure 1A). A broadened resonance at 3425 cm^–1^ is assigned to surface hydroxyl groups, i.e., Cu_2_O@(MEEA)(OH) (**1-OH)** (Scheme 1 and Figure S5). Synthesizing **1-OH** with 20 and 30 mol % of the ligand, H[MEEA], also provided NCs that were 3 nm in size (XRD; Figure S6). We propose that the nanocrystal size is determined by the rapid nucleation step, and the available ligands are distributed over the nanocrystal surface to give a variable coverage.^28,37^ We have previously identified these two regimes, nucleation controlled (fixed size, variable surface coverage) and coverage controlled (variable size, saturated coverage), in the synthesis of colloidal ZnO nanocrystals from ZnEt_2_.^37^

To probe the reactivity of the surface hydroxyl groups, **1-OH** was reacted with known quantities of nonanoic acid, increasing from 10 to 20 mol % (0.1–0.2 equiv vs. the starting copper concentration of 100 mol % or 1 equiv). After each addition, the resulting colloidal NC FT-IR spectrum showed a clear reduction in the intensity of the *ν*(O–H) stretch (Figure S7). Under these conditions, the nonanoic acid reacted selectively with the hydroxyl moieties; there was no evidence for any free carbonyl or hydroxide stretches from H[MEEA] or for any unreacted nonanoic acid. To probe the quantity of surface hydroxyl groups, **1-OH** was reacted stepwise with the same molar quantities of [Zn(C_6_F_5_)_2_]. The Cu–OH groups reacted with [Zn(C_6_F_5_)_2_] by rapid and irreversible protonolysis to form Cu–O–Zn(C_6_F_5_) complexes (see below). ^19^F{^1^H} NMR spectroscopy was used to monitor the reaction since for every surface Zn(II) complex formed, one equivalent of a fluoroaromatic byproduct also formed. As such, a ^19^F{^1^H} NMR spectroscopic titration was undertaken, whereby the complete reaction of the NC surface hydroxyl moieties correlates with the appearance of unreacted [Zn(C_6_F_5_)_2_] (Figures S9–S11). In both surface reactivity investigations, 0.2 equiv or 20 mol % (vs. total copper concentration) of nonanoic acid or [Zn(C_6_F_5_)_2_] resulted in a complete reaction of the surface −OH groups, i.e., saturation of the remaining surface Cu sites. To contextualize the experimental titrations, the initial surface coverages of the Cu_2_O NCs by both the MEEA ligands and hydroxyl moieties were estimated as follows. In the synthesis of **1-OH**, 10 mol % of the H[MEEA] carboxylic acid ligand was added relative to the Cu(I) precursor (100 mol %). As shown by IR spectroscopy and TGA analysis, the MEEA coordinates to but does not fully saturate the cuprous oxide surfaces (see the Supporting Information for estimates of surface coverage).

To investigate the coverage further, experimentally, reactions between **1-OH** and either 20 or 30 mol % [Zn(C_6_F_5_)_2_] relative to the starting Cu stoichiometry (100 mol %) were undertaken. In a toluene solution, at room temperature, **1-OZn(C**_**6**_**F**_**5**_**)** formed as a dark green solid (Scheme 1 and Figure S12). Analysis of the reaction supernatant, using ^19^F{^1^H} NMR spectroscopy, showed only the fluoroaromatic byproducts of protonolysis (∼1:1 C_6_HF_5_:(C_6_F_5_)_2_), for the 20 mol % reaction, but for the 30 mol % reaction significant quantities of unreacted [Zn(C_6_F_5_)_2_] were also observed (see the Supporting Information). These findings are consistent with an estimated surface Cu–OH content of around 20 mol % (see Supporting Information).

**1-OZn(C**_**6**_**F**_**5**_**)** was isolated by decantation of the residual toluene and repeatedly washed; powder XRD measurements confirm the retention of the cubic Cu_2_O phase with the same average crystallite size as the starting cuprous oxide NCs, i.e., **1-OH** (3 nm; see the Supporting Information).^28^ The product was characterized using solid-state ^19^F NMR spectroscopy, which showed broadened isotropic resonances at −117, −140 and −164 ppm. These peaks are assigned to the *o*-C_6_F_5_, *p*-C_6_F_5_, and *m*-C_6_F_5_ resonances of the Cu–O–Zn(C_6_F_5_) complexes (Figures S14 and S15).^57^ The FT-IR spectrum of **1-OZn(C**_**6**_**F**_**5**_**)** contains stretches associated with Zn–C_6_F_5_ groups at 1630, 1505, 1452, 1071, 1053, and 952 cm^–1^; these resonances were also observed in the spectrum of [Zn(C_6_F_5_)_2_] (Figures 1A and Supporting Information). Since the solid does not contain any unreacted [Zn(C_6_F_5_)_2_] (by ^19^F NMR spectroscopy), these resonances are assigned to Cu–O–Zn(C_6_F_5_) groups. The FT-IR spectrum also shows asymmetric and symmetric MEEA carboxylate stretches at 1605 and 1434 cm^–1^, respectively. The spectrum shows a characteristic MEEA *ν*(C–O–C) mode at 1107 cm^–1^, and the cuprous oxide NCs show a *ν*(Cu–O) mode at 629 cm^–1^.^29,58^ These resonances are at the same energy as those for **1-OH** and are consistent with MEEA remaining coordinated to the NP surface throughout the organometallic reaction (Figure 1A).

Due to the moisture sensitivity of **1-OZn(C**_**6**_**F**_**5**_**)**, thermal gravimetric analysis was conducted in a sealed aluminum pan, under N_2_. The TGA data show ∼15 wt % mass loss from 30 to 200 °C, consistent with thermolysis of C_6_F_5_ groups (Figure S18, N.B. [Zn(C_6_F_5_)_2_] also shows mass loss from 30 to 200 °C). TGA-MS at 100 °C shows a species with a *m*/*z* of 168, which is consistent with the loss of –C_6_F_5_ groups (Figure S19). The TGA data show a second 13 wt % mass loss from 250 to 300 °C, corresponding to the MEEA ligand loss (Figure 1B).^28^ Overall, the total organic fraction mass loss is 28 wt %, which is consistent with a theoretical 32 wt % ligand loading and with the formation of Cu_2_O and ZnO upon thermolysis. The TGA data contrast with those for the original**1-OH**, which show thermal reduction to Cu at temperatures above ∼250 °C, consistent with partial reduction and ligand loss; there was also a mass increase above ∼250 °C, representing partial reoxidation of surface Cu (Figures S4 and S49A).^28^

X-ray photoelectron spectroscopy (XPS) can be particularly useful for surface analysis of colloidal SC-NCs. The survey X-ray photoelectron (XP) spectra of **1-OZn(C**_**6**_**F**_**5**_**)** show all of the expected elements (Figure S20). The Cu 2p_3/2_ XP spectrum displays a main intensity peak at ∼932.5 eV and a low-intensity satellite peak at +13 eV from the main photoionization peak, indicative of Cu(I) (Figure 1C).^9,59,60^ The Cu(I) LMM Auger signal shows a kinetic energy of 916.6 eV, commensurate with Cu_2_O, and the valence band spectrum contains a peak position and shape that is also diagnostic of Cu_2_O (Figures S21 and S22).^9^ Multiple states are observed in the O 1s spectra; the lowest binding energy peak (∼530.4 eV) is attributed to a metal oxide environment (Cu–O–Cu/Zn), whereas the higher binding energy environments are associated with C=O and C–O environments of the MEEA carboxylate ligand (Figure 1D).^61,62^ Further, the C 1s spectra show chemical environments consistent with O=C–O, C–O, C–C, and C–H functionalities of the MEEA ligand (Figure S23).^61,62^ A signal is observed at 1022.0 eV in the Zn 2p_3/2_ spectrum (Figure 1E), indicative of Zn(II) surface speciation (Figure 1E).^63^ The F 1s spectrum shows two fluorine environments: a higher BE environment at ∼687.5 eV, which corresponds to C–F_*x*_ bonds in the –C_6_F_5_ ligand,^64,65^ and a lower binding energy environment at 685.0 eV, tentatively attributed to metal-fluoride (Zn–F) environments (Figure 1F).^65,66^ The F 1s XP data for [Zn(C_6_F_5_)_2_] also show these two environments (Figure S24); the Zn–F environment observed in **1-OZn(C**_**6**_**F**_**5**_**)** and [Zn(C_6_F_5_)_2_] is attributed to sample degradation during the XPS experiments. Taken altogether, the XP spectra indicate the successful installation of –OZn(C_6_F_5_) groups onto the Cu_2_O NC surface. ICP-MS experiments indicate an atomic ratio of ∼13:100 Zn:Cu in samples of **1-OZn(C**_**6**_**F**_**5**_**)**, consistent, within experimental error, with the expected 20 mol % value relative to copper (100 mol %) determined by other methods.

Given the successful installation of Zn(II) organometallic complexes onto the Cu_2_O surface, attention turned to the generality of the protonolysis reaction to other organometallic reagents. As such, treating **1-OH** with 20 mol % (0.2 equiv) of [Co(C_6_F_5_)_2_]·2THF relative to copper (100 mol %) formed **1-OCo(C**_**6**_**F**_**5**_**)** as a green powder featuring Cu–O–Co(C_6_F_5_) surface complexes (Scheme 1). Similar to **1-OZn(C**_**6**_**F**_**5**_**)**, powder XRD indicates retention of cubic Cu_2_O NC, with crystallite sizes remaining at ∼3 nm by Scherrer analysis (Figure S25). The solution-state ^19^F NMR spectrum of the reaction supernatant shows C_6_HF_5_ and (C_6_F_5_)_2_, i.e., the expected reaction byproducts (Figure S26). The FT-IR spectrum shows resonances consistent with OCo(C_6_F_5_) and MEEA ligands coordinated to the particle surface (see Supporting Information). The TGA data for **1-OCo(C**_**6**_**F**_**5**_**)** shows two “stages” of decomposition: ∼15 wt % loss occurs from 100 to 275 °C, and then ∼18 wt % loss from 275 to 350 °C. Similarly to **1-OZn(C**_**6**_**F**_**5**_**)**, these data are consistent with the loss of the –C_6_F_5_ and MEEA ligands. The overall organic ligand content matches the theoretical value closely (experimental = 33 wt %, theoretical = 32 wt %; see the Supporting Information). The survey XP spectrum shows all of the anticipated elements, and the Cu 2p_3/2_, C 1s, and O 1s XP spectra are consistent with those of **1-OH** and **1-OZn(C**_**6**_**F**_**5**_**)** (Supporting Information).

The F 1s XP spectrum shows two fluorine environments assigned to C–F*_x_* bonds in –C_6_F_5_ (∼687.5 eV)^64,65^ and those associated with CoF_2_ due to sample degradation (∼685 eV; Figure 1F). The Co 2p XP spectrum contains higher intensity peaks at 781 and 798 eV, which are commensurate with Co(II); satellites (S_X/Y_) located at higher binding energies provide further validation for this assignment (Figure S31).^67^ ICP-MS indicates there is 15 mol % Co relative to Cu (100 mol %) in samples of **1-OCo(C**_**6**_**F**_**5**_**)** which is consistent with the Zn(II) loading in the previous sample and with calculated values (theoretical = 20 mol %). Overall, the colloidal Cu_2_O NC can be reacted with different organometallic reagents to install a second metal. In the future, other s-, transition metal, and p-block organometallic reagents should be investigated.

One benefit of installing organometallic complexes is their potential to undergo further reactions. As proof of this concept, **1-OZn(C**_**6**_**F**_**5**_**)** was treated with three different carboxylic acids to form new surface-coordinated Zn(II)(carboxylate) complexes. In these experiments, it is important to avoid any ligand exchange reactions with Cu-MEEA ligands. The carboxylic acids were, therefore, selected to show lower acidity (higher p*K*_a_) than H[MEEA] (Table S3). Further, the significantly higher basicity of –C_6_F_5_ versus MEEA, together with the different denticity (i.e., monodentate –C_6_F_5_ versus bidentate carboxylate), should ensure that substoichiometric quantities of carboxylic acids react with the –OZn(C_6_F_5_) moieties rather than the MEEA ligands.

**Scheme 2:**
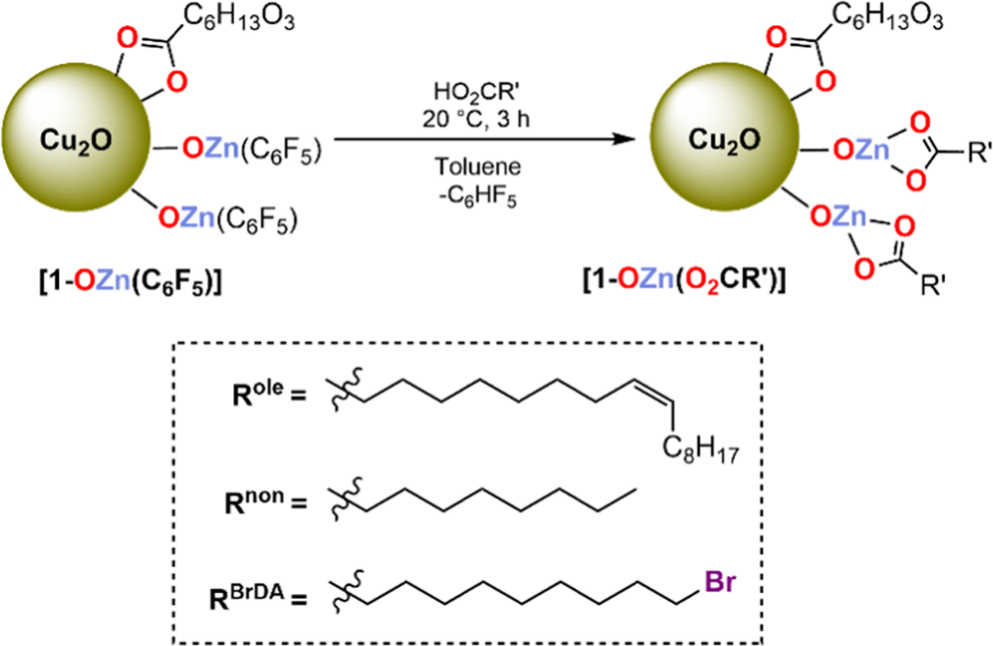
Illustrates the Reaction of 1-OZn(C_6_F_5_) with Carboxylic Acids to Form 1-OZn(O_2_CR’) Where HO_2_CR^ole^=oleic acid (HO_2_C(CH_2_)_7_CH = CH(CH_2_)_7_CH_3_), HO_2_CR^non^=nonanoic acid (HO_2_C(CH_2_)_7_CH_3_), and HO_2_CR^BrDA^=10-bromodecanoic acid (HO_2_C(CH_2_)_8_CH_2_Br).

Treating a slurry of **1-OZn(C**_**6**_**F**_**5**_**)** in toluene with 20 mol % (0.2 equiv) of HO_2_CR’, where R’ = R^ole^ is oleic acid, R^non^ is nonanoic acid, or R^BrDA^ is 10-bromodecanoic acid, relative to copper (100 mol %) resulted in solubilization to form dark green, colloidal solutions containing the products **1-OZn(O**_**2**_**CR’)** (Scheme 2, Figure S32). These solutions were stored as 36 mM solutions in toluene in the glovebox freezer. The dried NCs were analyzed by powder XRD, showing cubic Cu_2_O, with average crystallite sizes of 3 nm by Scherrer analysis; again, there was no change to the cuprous oxide nanocrystals during this reaction (Figures S33–S35). ICP-MS measurements indicate 23, 26, and 25 mol % Zn relative to Cu for R’ = R^ole^, R^non^, and R^BrDA^, respectively, in proximity to the anticipated value of 20 mol % Zn relative to Cu (100 mol %).

The ^19^F NMR spectra of the crude reaction mixtures confirmed the formation of the expected byproduct, C_6_HF_5_, without any remaining –Zn(C_6_F_5_) signals (Scheme 2 and Supporting Information). The ^1^H NMR spectrum of **1-OZn(O**_**2**_**CR**^**ole**^**)** in benzene-*d*_6_ shows broadened resonances, which are typical of such colloidal NCs. The resonance at ∼5.51 ppm (ω_1/2_ ∼ 20 Hz) is diagnostic of the internal alkene protons of the oleate ligand, and the line broadening is characteristic of nanoparticle surface coordination.^5^ The ^1^H NMR spectra of **1-OZn(O**_**2**_**CR**^**non**^**)** and **1-OZn(O**_**2**_**CR**^**BrDA**^**)** are similarly broadened to that for **1-OH**, consistent with carboxylate coordination (see the Supporting Information).^5^ Further, there were no signals observed for any free carboxylic acids, including original H[MEEA] pro-ligand, while stable colloids were retained. ^1^H NMR DOSY experiments for **1-OH** and **1-OZn(O**_**2**_**CR**^**ole**^**)** show that all relevant NMR signals diffuse at the same rate, indicative of the ligands being equivalent and remaining coordinated to the NC surface on the NMR time scale (Figures S45 and S46).

**Figure 2:**
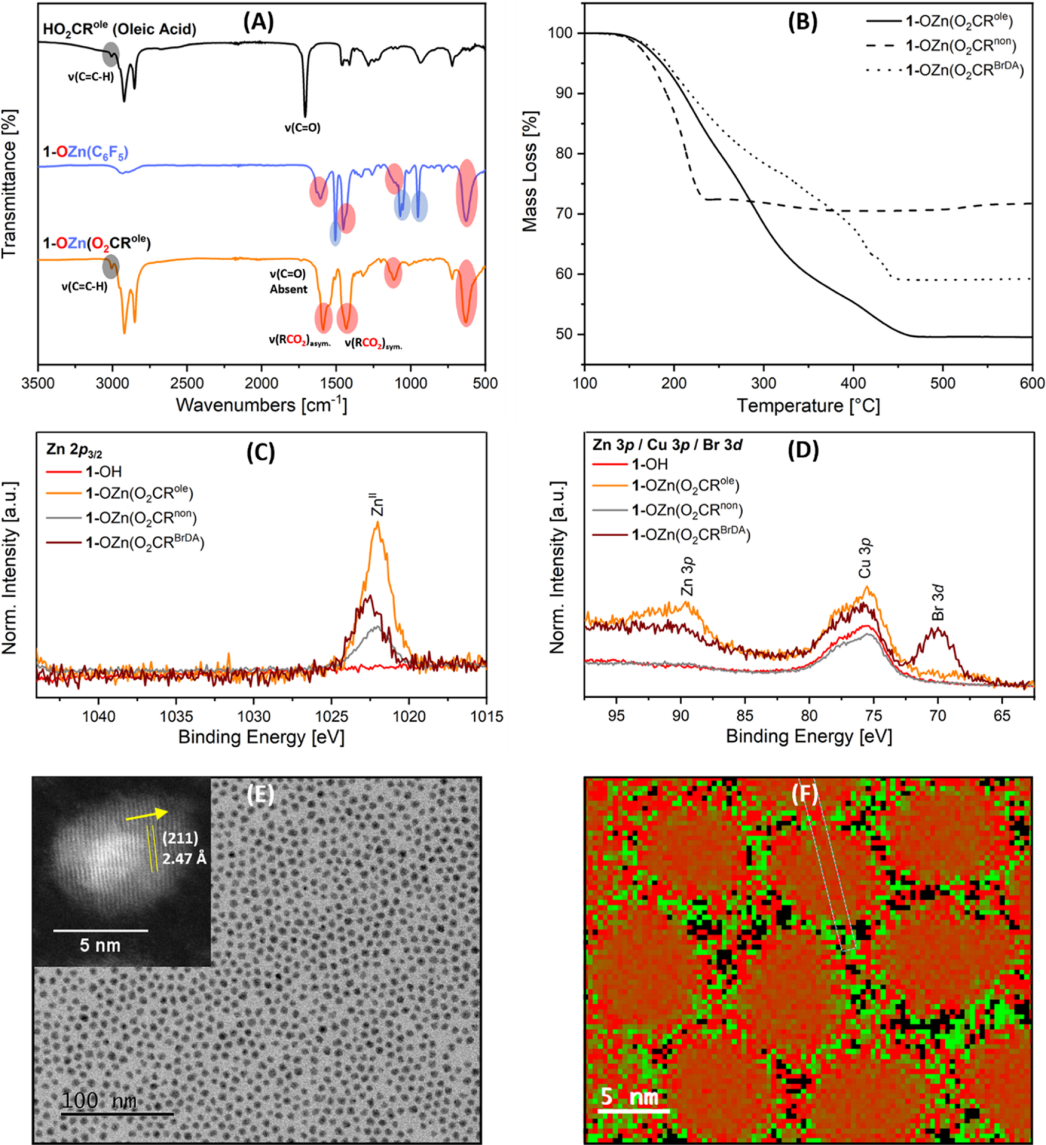
(A) Stacked FT-IR spectra of **HO**_**2**_**CR**^**ole**^, **1-OZn(C**_**6**_**F**_**5**_**)**, and **1-OZn(O**_**2**_**CR**^**ole**^**)**; red highlights represent the coordinated MEEA ligand, blue highlights represent –C_6_F_5_ groups, and black highlights represent the coordinated (O_2_CR^ole^) ligand. (B) Stacked TGA thermograms of **1-OZn(O**_**2**_**CR’)** (R’ = R^ole^, R^non^, R^BrDA^). (C) Zn 2p_3/2_ and (D) Zn 3p/Cu 3p/Br 3d XP spectra for **1-OH** and **1-OZn(O**_**2**_**CR’)**. (E) Annular bright-field STEM image with the HRTEM image and lattice fringes for **1-OZn(O**_**2**_**CR**^**ole**^**)**. (F) EELS analysis for **1-OZn(O**_**2**_**CR**^**ole**^**)**; red = Cu, green = Zn.

The FT-IR spectra for **1-OZn(O**_**2**_**CR’)** show asymmetric and symmetric carboxylate stretches at ∼1590–1530 and ∼1430–1410 cm^–1^, assigned to both the new carboxylates and MEEA ligands (Figure 2A and Supporting Information). Resonances at ∼1110 and ∼630 cm^–1^ are assigned to *ν*(C–O–C) of coordinated MEEA and ν(Cu–O) of Cu_2_O, respectively, and are consistent with **1-OH** and **1-OZn(C**_**6**_**F**_**5**_**)** (Figure 2A). The FT-IR spectrum of **1-OZn(O**_**2**_**CR**^**ole**^**)** shows a further diagnostic resonance at 3006 cm^–1^, assigned to the oleate alkene group C=C–H, and is useful to confirm the second carboxylate ligand coordination in the product. The FT-IR spectra of **1-OZn(O**_**2**_**CR’)** do not show any stretches for free carboxylic acids, indicating that on the faster time scale of IR spectroscopy (vs. NMR), all of the carboxylates are surface coordinated. Further, diagnostic IR resonances due to the Zn–C_6_F_5_ groups disappeared after the reaction.

Thermogravimetric analyses, conducted in air, of **1-OZn(O**_**2**_**CR’)** (R’ = R^ole^, R^BrDA^) and **1-OZn(O**_**2**_**CR**^**non**^**)** all show ligand mass loss onsets around ∼150 °C, with the later compound stabilising by 225 °C, and the others showing a broader decomposition range (Figure 2B). The total organic ligand contents are 50, 30, and 41 wt % for R’ = R^ole^, R^non^, and R^BrDA^, respectively, which closely match the expected values for ligand loss of 46, 31, and 43 wt %. These data are consistent with the complete reaction of the starting Zn(II) organometallic complex and further confirm that the MEEA ligand remains surface coordinated, and similarly to **1-M(C**_**6**_**F**_**5**_**)** (M = Zn, Co), are consistent with only loss of ligand (see Figure S50 and Table S4).

The XPS data for the **1-OZn(O**_**2**_**CR’)** samples show Cu 2p_3/2_ and Cu LMM Auger signals consistent with Cu_2_O NCs, similar to the previous XPS analysis (see Supporting Information). Both the C=O and M–O environments are observed in the O 1s spectra, as expected (Supporting Information). All of the samples show Zn 2p_3/2_ spectra consistent with Zn(II), and the peaks for **1-OZn(O**_**2**_**CR**^**BrDA**^**)** (1022.7 eV) are slightly shifted relative to **1-OZn(C**_**6**_**F**_**5**_**)**, **1-OZn(O**_**2**_**CR**^**ole**^**)**, and **1-OZn(O**_**2**_**CR**^**non**^**)** (Figures 1E and 2C). Importantly, the Br 3d XP spectrum of **1-OZn(O**_**2**_**CR**^**BrDA**^**)** contains a signal at 70 eV, which is typical of C–Br_*x*_, suggesting that the carboxylate ligand is surface coordinated (Figure 2D).^68^ The samples did not show any F 1s signals (Figure S56).

The colloidal NC featuring Zn(carboxylate) complexes were no longer air-sensitive, and selected samples of **1-OZn(O**_**2**_**CR’)** were analyzed by TEM. The data show small, uniform colloidal nanoparticles; **1-OZn(O**_**2**_**CR**^**ole**^**)** shows an average diameter of 5.5 ± 1.4 nm and **1-OZn(O**_**2**_**CR**^**non**^**)** an average size of 5.0 ± 1.4 nm for (Figure 2E and Supporting Information). The particle sizes following surface functionalization with –OZn(O_2_CR’) are consistent with the starting **1-OH** sample (6.0 ± 1.5 nm, Supporting Information). HRTEM shows the (211) lattice fringe at values of 2.47 Å for R’ = R^ole^, 2.54 Å for R’ = R^non^ (Figure 2E and Supporting Information). The EELS data for **1-OZn(O**_**2**_**CR**^**ole**^**)** and **1-OZn(O**_**2**_**CR**^**non**^**)** show signals corresponding to both Cu and Zn, with Zn being clearly observed on the nanocrystal surfaces (Figure 2F and Supporting Information). The measured Zn content is around 25 mol % related to Cu, which agrees well with the anticipated value of 20 mol %.

**Figure 3:**
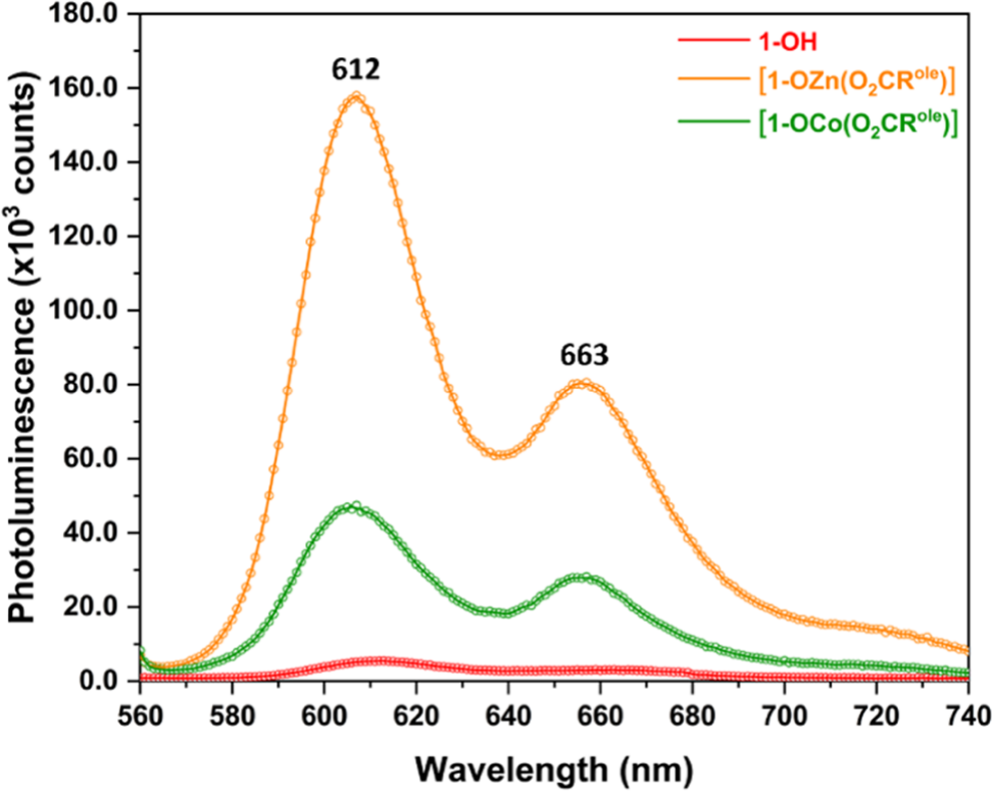
Stacked photoluminescence spectra of **1-OH**, **1-OZn(O**_**2**_**CR**^**ole**^**)**, and **1-OCo(O**_**2**_**CR**^**ole**^**)**. In each case, the solution concentration is 0.18 mM in toluene. Experimental data (open circles) and fitted spectrum (smooth line).

To evaluate the effects of organometallic surface functionalization on the optical properties of the parent cuprous oxide NCs, photoluminescence (PL) spectroscopy was conducted on **1-OH**, **1-OZn(O**_**2**_**CR**^**ole**^**)**, and **1-OCo(O**_**2**_**CR**^**ole**^**)**; the latter material was synthesized by treating **1-OCo(C**_**6**_**F**_**5**_**)** with oleic acid, HO_2_CR^ole^, in toluene at room temperature and was characterized by FT-IR spectroscopy (Figure S68). In all three samples, two emission bands of the same relative intensity are observed at 612 and 663 nm, arising from an identical Cu_2_O NC core (Figure 3). The partially covered colloidal NC, **1-OH**, exhibits very low PL intensity (Figure S69). However, after organometallic surface functionalization, there is a dramatic increase in emission intensity at identical colloid concentrations; the increases are 30-fold and 10-fold for **1-OZn(O**_**2**_**CR**^**ole**^**)** and **1-OCo(O**_**2**_**CR**^**ole**^**)**, respectively (Figures 3 and S70–S72). The effect is most pronounced for Zn(II) decoration, as might be expected given its filled d-shells, which provide no obvious recombination pathway. It is well-known that SC-NCs often require core–shell structures to show efficient emission.^69^ The remarkable observation that the addition of precisely coordinated, single metal complexes on the surface also improves emission intensity shows that nonradiative decay mechanisms (through surface defects or interactions with solvent molecules) are suppressed without requiring a full “shell.” Simply passivating the unsaturated surface −OH sites is sufficient.

## Conclusions

In summary, small (3–6 nm) colloidal cuprous oxide nanocrystals were successfully functionalized with Zn(II) or Co(II) complexes. The reactions between cuprous oxide colloidal NCs, specifically Cu–OH surface ligands, and [Zn(C_6_F_5_)_2_] or [Co(C_6_F_5_)_2_]·2THF occurred at room temperature and in bulk, in organic solvents. The quantitative reactions allowed for precise surface functionalization with both organic ligands (carboxylates) and organometallic complexes. The zinc complexes were further reacted with other carboxylic acids to form surface-coordinated Zn-carboxylate complexes. Surface organometallic functionalization showed a dramatic increase in photoluminescence relative to that of the parent Cu_2_O NC; the effect was more pronounced for surface functionalization with Zn(II) than with Co(II). The finding demonstrates the important influence of secondary functionalization on properties and may be directly relevant to fluorescence applications. The deposition process is self-limiting since the –OM(R) formation depends on direct reaction with the Cu–OH groups. Complementary analytical techniques, such as powder XRD, FT-IR, ^19^F NMR spectroscopy, TGA, TGA-MS, ICP-MS, XPS, TEM, and EELS, all confirmed NC functionalization with both organic (carboxylate) and organometallic/inorganic complexes. As illustrated, this approach provides a quantitative analytical probe of reactive surface sites on nanoparticles that are often overlooked. Sequential, self-limiting reactions allow multiple functions to be systemically introduced with atomic precision. This colloidal NC surface modification strategy is amenable to many different organometallic reagents and may be applied to a wide range of NC cores, particularly oxides, but extending to sulfides^28^ and other systems. For example, partially hydroxyl terminated colloidal ZnO nanocrystals^37^ formed Zn–O–Cu bridges with Cu nanoparticles deposited from mesityl copper;^8^ single copper center surface groups should be accessible under more controlled conditions. Precise nanoparticle multifunctionalization is expected to be important for applications including theranostics, (photo)catalysis, and photo/electrical applications.

## Supporting Information Available

Full experimental details and procedures and characterization data (PXRD, FT-IR, TGA, ^19^F NMR, XPS, TEM, and PL) (PDF)
