# Supplementary material for: Exploiting Organometallic Chemistry to Functionalize Small Cuprous Oxide Colloidal Nanocrystals
Source: J Am Chem Soc. 2024 Feb 1;146(6):3816–24. doi: 10.1021/jacs.3c10892 (PMC10870705; doi:10.1021/jacs.3c10892)
Supplement: Supplementary file 1 — ja3c10892_si_001.pdf [file ja3c10892_si_001.pdf]

Electronic Supplementary Information

Exploiting Organometallic Chemistry to Functionalize Small Cuprous Oxide Colloidal Nanocrystals

Bradley E. Cowie[a], Kristian L. Mears[a], Mark S'Ari[b], Ja Kyung Lee[b], Martha Briceno de Gutierrez[b], Curran Kalha[c], Anna Regoutz[c], Milo S. P. Shaffer*[d,e], Charlotte K. Williams*[a]

[a] Department of Chemistry, University of Oxford, Chemistry Research Laboratory, 12 Mansfield Road, Oxford, OX1 3TA, U.K.
[b] Johnson Matthey, Johnson Matthey, Blounts Court, Sonning Common, Reading, RG4 9NH, U.K.
[c] Department of Chemistry, University College London, 20 Gordon Street, London, WC1H 0AJ, U.K.
[d] Department of Materials, Imperial College London, London, SW7 2AZ, U.K.
[e] Department of Chemistry, Imperial College London, 82 Wood Lane, London W12 0BZ, U.K.

Corresponding author email addresses: Charlotte.williams@chem.ox.ac.uk; m.shaffer@imperial.ac.uk

Table of Contents

**General Details**	**2**
**Surface Coverage Titration Experiments**	**4**
**Syntheses**	**5**
• Cu_2O@(MEEA){OZn(C_6F_5)} (**1-OZn(C_6F_5)**)	**5**
• Cu_2O@(MEEA){OCo(C_6F_5)} (**1-OCo(C_6F_5)**)	**5**
• Cu_2O@(MEEA){OZn(O_2CR^{ole})} (**1-OZn(O_2CR^{ole})**)	**5**
• Cu_2O@(MEEA){OZn(O_2CR^{non})} (**1-OZn(O_2CR^{non})**)	**5**
• Cu_2O@(MEEA){OZn(O_2CR^{BrDA})} (**1-OZn(O_2CR^{BrDA})**)	**5**
• Cu_2O@(MEEA){OCo(O_2CR^{ole})} (**1-OCo(O_2CR^{ole})**)	**6**
**Determination of Nanoparticle Surface Coverage**	**7**
**Experimental Spectra and Data**	**8**
**References**	**68**

General Details

All manipulations were conducted inside a nitrogen-filled glove box or on a double-manifold Schlenk line equipped with a nitrogen atmosphere using proper Schlenk technique.[1] Toluene was obtained from a solvent purification system and stored, under an N_2 atmosphere over molecular sieves (3 Å), in an ampoule equipped with a Young's tap. Benzene-d_6 was purchased from Cambridge Isotope Laboratories Inc., dried over molecular sieves (3 Å) and distilled prior to use.

Mesitylcopper(I), $[CuMes]_z$ (z = 4, 5), was purchased from Strem Chemicals, recrystallized from toluene (80 mL for 5 g of $[CuMes]_z$) at –30 °C and stored in the glove box freezer; $[CuMes]_z$ is thermally and photochemically sensitive, so its manipulation was kept to a minimal. $[Zn(C_6F_5)_2]$ was purchased from Sigma Aldrich and stored in the glove box prior to use. 2-[2-(2-methoxyethoxy)ethoxy]acetic acid, H[MEEA], and nonanoic acid, HO_2CR^{non}, were purchased from Sigma Aldrich, distilled under dynamic vacuum and stored in the glove box prior to use. Oleic acid, HO_2CR^{ole}, was purchased from fluorochem, distilled under dynamic vacuum and stored in the glove box prior to use. 10-bromodecanoic acid, HO_2CR^{BrDA}, was purchased from fluorochem and stored in the glove box prior to use. Cu_2O@(MEEA)(OH) (**1-OH**)[2] was synthesized according to the literature procedure.

Solution-state 1D ^1H and $^{19}F\{1H\}$ NMR spectra, and ^1H DOSY NMR spectra were obtained on a Bruker AV-400 NMR spectrometer at room temperature; ^1H NMR spectra are referenced relative to $SiMe_4$ (0 ppm) through a resonance of the employed deuterated solvent or proteo impurity of the solvent (benzene-d_6 = 7.16 ppm), and ^{19}F NMR spectra are referenced externally relative to α,α,α-trifluorotoluene (–63.8 ppm). Solid-state $^{19}F\{^1H\}$ NMR spectra were obtained on a Bruker AVIII 400 MHz spectrometer at room temperature at three different spinning speeds (17, 20, 22 kHz) and referenced externally relative to Teflon at –123.2 ppm (relative to $CFCl_3$ at 0 ppm). FT-IR spectra were obtained on a Shimadzu IRSpirit spectrometer, fitted with a KBr window and DLATGS detector with temperature control. FT-IR spectra were recorded inside a glove box using a single reflection ATR accessory and measured in transmission scanning mode. Samples were drop-cast onto the sample holder from toluene and scanned from 4700-340 cm^{-1} (100 scans, 4 cm^{-1} resolution).

Powder X-ray diffraction experiments were performed using a PANalytical Xpert Pro diffractometer, using a Cu Kα radiation source (λ = 0.154 nm) at 40 mA and 40 kV with a step size of 0.033° 2θ, scan step time of 70 s and scan range of 5–90° 2θ. Baseline corrections were processed using Fityk Software (version 1.3.1; Marcin Wojdyr, 2010),[3] and line fittings were processed using either Fityk Software[3] or Origin2020. The average crystallite size (*D*) was estimated according to the Scherrer equation, $D = k\lambda/\beta\cos\theta$, where β is the full width at half maximum (FWHM) of the diffraction peak after instrumental broadening correction and k is the shape factor for the average crystallite. β is calculated from $\beta^2 = \beta_o^2 - b^2$, where β_o is the measured FWHM of the sample following fitting to a Gaussian function, and b is the measured FWHM of a well-crystallized material (LaB_6, 99.5%, Alfa Aesar) to account for instrument broadening and k = 0.9 for powders, assuming spherical shape. Air-sensitive samples were prepared in a glovebox by drop casting a toluene solution of the sample onto a glass slide; samples were then placed into a sealed sample holder.

Thermal gravimetric analysis thermograms were collected on a TGA5500 System (TA Instruments) equipped with the TRIOS software package. Samples of **1-OM(C_6F_5)** (M = Zn, Co) were analyzed in an N_2 atmosphere in sealed 80 µL aluminum pans (TA Instruments), whereas samples of **1-OZn(O_2CR')** (R' = ole, non, BrDA) were analyzed in air in 100 µL platinum bails (TA Instruments). Samples of **1-OZn(O_2CR')** were heated to 100 °C at a rate of 5 °C per minute, held for 15 minutes to ensure the removal of excess solvent/moisture, then heated to 600 °C at a rate of 5 °C per minute. Samples of **1-OM(C_6F_5)** were heated directly to 600 °C at a rate of 5 °C per minute as the onset of -C_6F_5 loss was observed at ~40 °C. TGA curves were normalized to 100 % following any residual solvent loss. Thermal gravimetric analysis mass spectrometry for **1-OZn(C_6F_5)** was obtained with a Netzsch STA 449 F1 Jupiter TGA/DTA coupled with a GMS 403 D Aëolos mass spectrometer. The analysis was performed under a N_2 atmosphere, and the heating sequence for the sample of **1-OZn(C_6F_5)** was as follows: Segment 1 = ramp from 20 to 100 °C at 2 °C/minute (40 minutes); Segment 2 = isotherm at 100 °C for 15 minutes; Segment 3 = ramp from 100 to 600 °C at 2 °C/minute.

ICP-MS measurements were performed by Dr Nigel Howard (University of Cambridge) and Trang To (University of Oxford). Atomic ratios of Zn and Co relative to Cu (100 %) are reported. The standard deviation on Zn and Cu for samples of **1-OZn(C_6F_5)** and **1-OZn(O_2CR')** (R' = R^{ole}, R^{non}, R^{BrDA}) are 0.6 and 0.04 ppb, respectively, and those on Co and Cu for samples of **1-OCo(C_6F_5)** are 3 and 21 ppb, respectively. Repeat ICP-MS experiments indicate

there is less Zn and Co relative to Cu in samples of **1-OM(C_6F_5)** (M = Zn, Co) than expected (10-13 % Zn relative to Cu, and 15 % Co relative to Cu vs. ~20 % expected), despite values of ~20 % Zn relative to Cu is being observed for **1-OZn(O_2CR')** (R' = R^{ole}, R^{non}, R^{BrDA}), which are obtained through the addition of carboxylic acid, HO_2CR', to **1-OZn(C_6F_5)**, without additional Zn added. Lower concentrations of Zn and Co relative to Cu for **1-OM(C_6F_5)** have been attributed to the air- and moisture-sensitivity of these samples.

X-ray photoelectron spectroscopy (XPS) was used to characterize the surface of the nanocrystals. The spectra were recorded on a Thermo Scientific K-Alpha X-ray photoelectron spectrometer system operating at $1x10^{-8}$ mbar base pressure. This system incorporates a monochromated, microfocused Al Kα X-ray source (hν = 1486.6 eV) and a 180° double focusing hemispherical analyser with a 2D detector. An X-ray spot size of 400 µm was used and the X-ray source was operated at 6 mA emission current and 12 kV anode bias. A flood gun was used to minimize sample charging. Samples were mounted using conductive carbon tape and transferred to the spectrometer using a special glove box module which ensured that samples were never exposed to air. Data were collected at 200 eV pass energy for survey spectra, and 20 eV pass energy for both core level and valence band spectra. All data were analyzed using the Avantage software package.

Transmission electron microscope (TEM) samples were prepared by drop-casting diluted colloidal solutions (toluene, 36 mM) onto ultrathin (~3 nm) carbon films on lacy carbon support film, 300 mesh, gold TEM grids (Agar scientific) while in a glove box. TEM images were acquired on a Cs aberration corrected Titan 80/300 TEM/STEM microscope operated at 300 kV and equipped with a Bruker XFlash EDS detector and Gatan Tridiem Giff.

Fluorescence spectra were acquired at 298 K using an Edinburgh Instruments FS5 spectrofluorometer operating Fluoracle® software and equipped with a xenon arc lamp (providing 230–1000 nm excitation range), a thermostatic sample holder (SC-20) and both an R13456 PMT detector (200–950 nm spectral coverage, Hamamatsu) and an InGaAs analogue NIR detector (850–1650 nm spectral coverage). Spectroscopic measurements were conducted using quartz cuvettes (10 mm path length, Starna Scientific Ltd).

Surface Coverage Titration Experiments:

General Method: The number of surface -OH groups in **1-OH** was probed through the addition of nonanoic acid to Cu_2O@MEEA and evaluating the disappearance of the O-H stretch in the resulting IR spectra, as well as through the addition of [Zn(C_6F_5)$_2$] to Cu_2O@MEEA and evaluating the appearance of unreacted [Zn(C_6F_5)$_2$] by ^{19}F NMR.

1-OH + Nonanoic Acid: A solution of nonanoic acid (10 mol%, 10.9 mg, 6.89×10^{-2} mmol) in toluene (0.5 mL) was added dropwise to a stirring solution of **1-OH** (18.9 mL, [Cu] = 36 mM) in toluene in a 30 mL vial inside the glove box. The reaction was stirred for 2 hours at room temperature; no colour change was observed. The degree of consumption of surface -OH groups was then evaluated by FT-IR, and it was found that the O-H stretch was still present. Additional nonanoic acid was then added in increments ((1) 2.2 mg, 1.39×10^{-2} mmol; (2) 3.2 mg, 2.02×10^{-2} mmol; (3) 3.0 mg, 1.90×10^{-2} mmol; (4) 3.0 mg, 1.90×10^{-2} mmol) as solutions in toluene (0.5 mL). Following each addition, the reaction was stirred for 2 hours at room temperature; the degree of consumption of surface -OH groups was then evaluated by FT-IR. Following the final addition, surface-OH groups were no longer observed.

1-OH + 20 mol% [Zn(C_6F_5)$_2$]: A solution of [Zn(C_6F_5)$_2$] (43.1 mg, 0.108 mmol) in toluene (~3 mL) was added dropwise to a stirring toluene solution of **1-OH** (15 mL; [Cu] = 36 mM) in a 30 mL vial inside the glove box. The reaction was stirred for 2 hours at room temperature, resulting in precipitation of a dark green powder. The degree of consumption of [Zn(C_6F_5)$_2$] to form surface -OZn(C_6F_5) groups was then evaluated by ^{19}F NMR.

1-OH + 30 mol% [Zn(C_6F_5)$_2$]: A solution of [Zn(C_6F_5)$_2$] (8.6 mg, 2.15×10^{-2} mmol) in toluene (~0.5 mL) was added dropwise to a stirring toluene solution of **1-OH** (2.0 mL; [Cu] = 36 mM) in a 10 mL vial inside the glove box. The reaction was stirred for 2 hours at room temperature, resulting in precipitation of a dark green powder. The degree of consumption of [Zn(C_6F_5)$_2$] to form surface -OZn(C_6F_5) groups was then evaluated by ^{19}F NMR.

Syntheses:

Cu_2O@(MEEA){OZn(C_6F_5)} (1-OZn(C_6F_5)): A solution of [Zn(C_6F_5)$_2$] (144 mg, 0.361 mmol) in toluene (~5 mL) was added dropwise to a stirring solution of **1-OH** (50 mL, 18 mM in toluene) in a 30 mL vial inside the glove box. The solution was stirred for 2 hours at room temperature, during which time **1-OZn(C_6F_5)** precipitated as a dark green solid. The reaction solution was left to sit to allow the solid to settle, and the supernatant was decanted. The remaining solid was washed with toluene (3 × 10 mL) and then dried under reduced pressure. Isolated yield = 129 mg (68 %). PXRD: 2θ = 30, 37, 43, 62, 75° (Cubic Cu_2O; JCPDS 00-002-1067). FT-IR ν_{max}/cm^{-1}: 2933 (C–H, m), 2893 (C–H, m), 1630 (C=C in C_6F_5, m), 1605 (asym. RCO_2, m), 1505 (C–F in C_6F_5, s), 1452 (sym. RCO_2 + C–F in C_6F_5, s), 1434 (sym. RCO_2, s), 1375 (w), 1354 (w), 1332 (w), 1260 (w), 1202 (w), 1107 (C–O of RCO_2, m), 1091 (C–O of RCO_2, m), 1071 (C–F in C_6F_5, s), 1053 (C–F in C_6F_5, s), 1012 (w), 952 (C–F in C_6F_5, s), 876 (w), 841 (w), 785 (s), 629 (s). ICP-MS: Zn = 186 ppb; Cu = 1481 ppb (13:100 Zn:Cu).

Cu_2O@(MEEA){OCo(C_6F_5)} (1-OCo(C_6F_5)): A solution of [Co(C_6F_5)$_2$]·2THF (108 mg, 0.201 mmol) in toluene (~4 mL) was added dropwise to a stirring solution of **1-OH** (27.9 mL, 0.18 mM in toluene) in a 30 mL vial inside the glove box. The solution was stirred for 2 hours at room temperature, during which time **1-OCo(C_6F_5)** precipitated as a dark green solid. The reaction solution was then left to sit to allow the solid to settle, and the supernatant was decanted. The remaining solid was washed with toluene (3 × 10 mL) and then dried under reduced pressure. Isolated yield = 70 mg (67 %). PXRD: 2θ = 30, 37, 43, 62, 74, 78° (Cubic Cu_2O; JCPDS 00-002-1067). FT-IR ν_{max}/cm^{-1}: 2940 (C–H, w), 2882 (C–H, w), 1628 (C=C in C_6F_5, m), 1604 (asym. RCO_2, m), 1500 (C–F in C_6F_5, s), 1438 (sym. RCO_2 + C–F in C_6F_5, s), 1424 (sym. RCO_2, s), 1330 (w), 1248 (w), 1200 (w), 1116 (C–O in RCO_2, m), 1090 (C–O in RCO_2, m), 1066 (C–F in C_6F_5, s), 1044 (C–F in C_6F_5, s), 1009 (w), 943 (C–F in C_6F_5, s), 890 (w), 845 (w), 781 (w), 721 (w), 632 (s). ICP-MS: Co = 605 ppb; Cu = 4012 ppb (15:100 Co:Cu).

Cu_2O@(MEEA){OZn(O_2CR^{ole})} (1-OZn(O_2CR^{ole})): A solution of oleic acid (HO_2CR^{ole}; 43.7 mg, 0.155 mmol) in toluene (~2 mL) was added dropwise to a stirring slurry of **1-OZn(C_6F_5)** (81.4 mg, 0.387 mmol) in toluene (21.5 mL) in a 30 mL vial inside the glove box. The solution was stirred overnight at room temperature, during which time the initially insoluble green powder re-dissolved in toluene, providing **1-OZn(O_2CR^{ole})** as a dark green colloidal solution. **1-OZn(O_2CR^{ole})** was stored as a colloidal solution in toluene. ^{1}H NMR (benzene-d_6, 298 K, 400 MHz): δ 5.51 (broad s, RC*H*=C*H*R', ~2 H), 2.11 (broad s, ~4H), 1.37, 1.30 (2×broad s, ~21H), 0.93 (broad s, ~6H). PXRD: 2θ = 30, 37, 43, 62, 75° (Cubic Cu_2O; JCPDS 00-002-1067). FT-IR ν_{max}/cm^{-1}: 3006 (C–H of RC*H*=C*H*R', w), 2953 (C–H, m), 2920 (C–H, s), 2852 (C–H, s), 1588, 1544 (asym. RCO_2, s), 1507 (w), 1431 (sym. RCO_2, s), 1321 (w), 1200 (w), 1112 (C–O of RCO_2, m), 724 (m), 629 (s). ICP-MS: Zn = 71.2 ppb; Cu = 305 ppb (23:100 Zn:Cu).

Cu_2O@(MEEA){OZn(O_2CR^{non})} (1-OZn(O_2CR^{non})): A solution of nonanoic acid (HO_2CR^{non}; 17.3 mg, 0.109 mmol) in toluene (~2 mL) was added dropwise to a stirring slurry of **1-OZn(C_6F_5)** (57.2 mg, 0.272 mmol) in toluene (15 mL) in a 30 mL vial inside the glove box. The solution was stirred overnight at room temperature, during which time the initially insoluble green powder re-dissolved in toluene, providing **1-OZn(O_2CR^{non})** as a dark green colloidal solution. **1-OZn(O_2CR^{non})** was stored as a colloidal solution in toluene. ^{1}H NMR (benzene-d_6, 298 K, 400 MHz): δ 3.34 (broad s, ~4 H), 1.37, 1.30, 1.22, 1.16 (4×broad s, ~9H), 0.92, 0.77 (2×broad s, ~4H). PXRD: 2θ = 30, 37, 43, 62, 75° (Cubic Cu_2O; JCPDS 00-002-1067). FT-IR ν_{max}/cm^{-1}: 2938 (C–H, m), 2904 (C–H, s), 2855 (C–H, m), 2835 (C–H, s), 1570, 1536 (asym. RCO_2, s), 1422, 1408 (sym. RCO_2, s), 1311 (w), 1237 (w), 1194 (w), 1104 (C–O of RCO_2, m), 1007 (w), 953 (m), 884(w), 846 (w), 809 (w), 769 (w), 739 (w), 718 (m), 623 (s). ICP-MS: Zn = 17.7 ppb; Cu = 67.3 ppb (26:100 Zn:Cu).

Cu_2O@(MEEA){OZn(O_2CR^{BrDA})} (1-OZn(O_2CR^{BrDA})): A solution of 10-bromodecanoic acid (HO_2CR^{BrDA}; 45.2 mg, 0.180 mmol) in toluene (~2 mL) was added dropwise to a stirring slurry of **1-OZn(C_6F_5)** (94.7 mg, 0.450 mmol) in toluene (25 mL) in a 40 mL vial inside the glove box. The solution was stirred overnight at room temperature, during which time the initially insoluble green powder re-dissolved in toluene, providing **1-OZn(O_2CR^{BrDA})** as a dark green colloidal solution. **1-OZn(O_2CR^{BrDA})** was stored as a colloidal solution in toluene. ^{1}H NMR (benzene-d_6, 298 K, 400 MHz): δ 3.01, 2.92 (2×broad s, ~2H), 1.54 (broad s, ~2H), 1.36 (broad s, ~4H), 1.36–0.68 (broad m, ~10H). PXRD: 2θ = 37, 43, 62, 74° (Cubic Cu_2O; JCPDS 00-002-1067). FT-IR ν_{max}/cm^{-1}: 2941 (C–H, w), 2903 (C–H, s), 2833 (C–H, s), 1578, 1542 (asym. RCO_2, s), 1501 (w), 1421, 1409 (sym. RCO_2, s), 1310 (w), 1252 (w), 1238 (w), 1226 (w), 1194 (w), 1103 (C–O of RCO_2, m), 719 (m), 640 (s), 560 (m). ICP-MS: Zn = 22.0 ppb; Cu = 90.0 ppb (25:100 Zn:Cu).

Cu_2O@(MEEA){OCo(O_2CR^{ole})} (1-OCo(O_2CR^{ole})): A solution of oleic acid (HO_2CR^{ole}; 69.4 mg, 0.246 mmol) in toluene (1.0 mL) was added dropwise to a stirring slurry of **1-OCo(C_6F_5)** (20.6 mg, 0.098 mmol) in toluene (9.0 mL) in a 30 mL vial inside the glove box. The solution was stirred overnight at room temperature, during which time the initially insoluble green powder re-dissolved in toluene, providing **1-OCo(O_2CR^{ole})** as a dark green colloidal solution. **1-OCo(O_2CR^{ole})** was stored as a colloidal solution in toluene. FT-IR ν_{max}/cm^{-1}: 2990 (C–H of RC*H*=C*H*R', w), 2906 (C–H, m), 2836 (C–H, s), 2852 (C–H, s), 1575, 1541 (asym. RCO_2, s), 1451 (w), 1408 (sym. RCO_2, s), 1308 (w), 1255 (w), 1087, 1013 (C–O of RCO_2, m), 717 (m), 686 (s).

Determination of Nanoparticle Surface Coverage:

The method for determining the theoretical nanoparticle surface coverage by ligand coordination has been adapted from the literature.[4]

The theoretical total surface area of a nanoparticle sample, S_T, is:

$$S_T = \frac{3nV_m}{r}$$

where n is the number of moles of Cu_2O, V_m is the molar volume of Cu_2O, and r is the particle radius, in Å, from TEM measurements (Figure S3); see Tables S1 and S2 for the appropriate values.

Next, the number of moles of ligand (i.e. MEEA and OH) per mole of Cu_2O, η_C, was calculated and determined using the following equation:

$$\eta_C = \frac{(\frac{W_O}{M_O})}{(\frac{W_i}{M_i})}$$

where W_O and W_i are the weight percent (wt%) of organic and inorganic components, respectively, and M_O and M_i are the molecular weight of the ligand and that of Cu_2O, respectively. See Tables S1 and S2 for the appropriate values.

Assuming that all ligands are bound to the surface of the nanoparticle and form a close packed monolayer, the theoretical surface area occupied by ligand coordination, S_S, was then calculated using the following equation:

$$S_S = Z \times \eta_C \times Surface\ Area\ per\ Ligand$$

where Z is Avogadro's number. The MEEA ligand is estimated to cover an area of 20.5 Å^2,[5] and surface area per OH groups was estimated as 7.8 Å^2 using a similar method to Tasker and co-workers.[6]

Finally, the ratio of S_S to S_T provides an estimated percentage surface coverage (Table S2).

Table S1. Summarized values from theoretical nanoparticle surface coverage calculations.

Material	n	V_m [Å^3 mol^{-1}]	Particle Size, r, by TEM [Å]	W_O [%]	M_O [g mol^{-1}]	W_i [%]
**Cu_2O@(MEEA)$_{0.1}$**	1	2.385×10^{25}	30	12.4	177.2	87.6
**Cu_2O@(OH)$_{0.2}$**	1	2.385×10^{25}	30	2.4	17.0	97.6

Table S2. Summarized values from theoretical nanoparticle surface coverage calculations.

Material	M_i [g mol^{-1}]	Surface Area per Ligand [Å^2]	Estimated Particle Surface Area [S_T, Å^2 mol^{-1}]	Moles of Ligand per Mole of Particle [η_C, mol]	Estimated Surface Area Occupied by Ligand [S_S, Å^2]	Surface Coverage [%]
**Cu_2O@(MEEA)$_{0.1}$**	143.1	20.5	2.385×10^{24}	0.1143	1.411×10^{24}	59
**Cu_2O@(OH)$_{0.2}$**	143.1	7.8	2.385×10^{24}	0.2069	9.768×10^{23}	41

Experimental Spectra and Data:

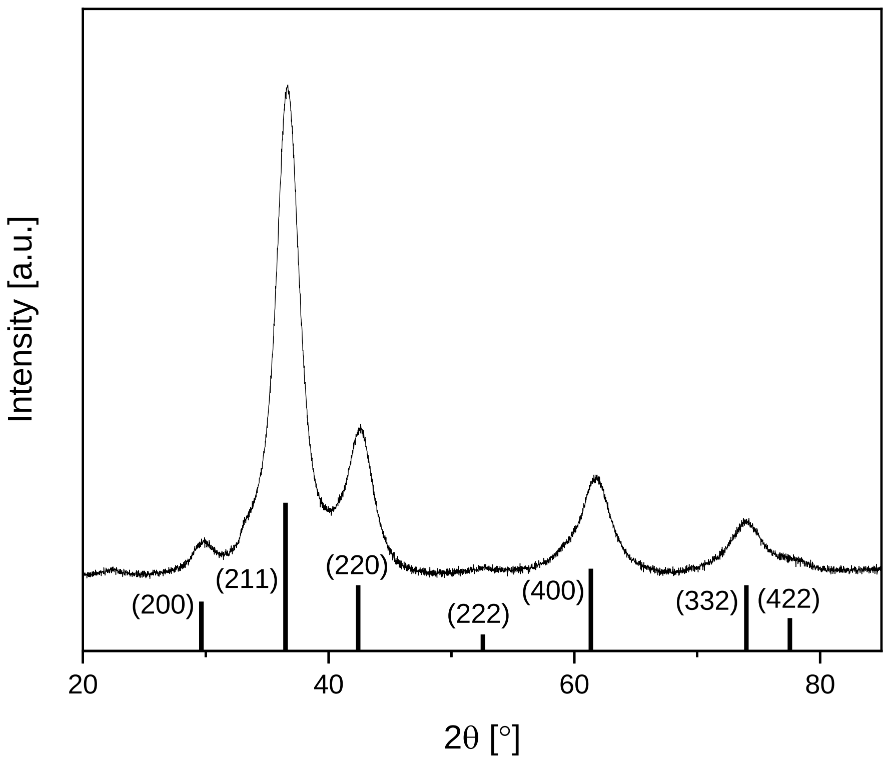

Figure S1. Powder X-ray diffraction pattern of Cu_2O@(MEEA)(OH) (**1-OH**); the crystallites are 3 nm (Scherrer analysis). The diffraction pattern is indexed against cubic Cu_2O as vertical bars (JCPDS 00-002-1067).[2]

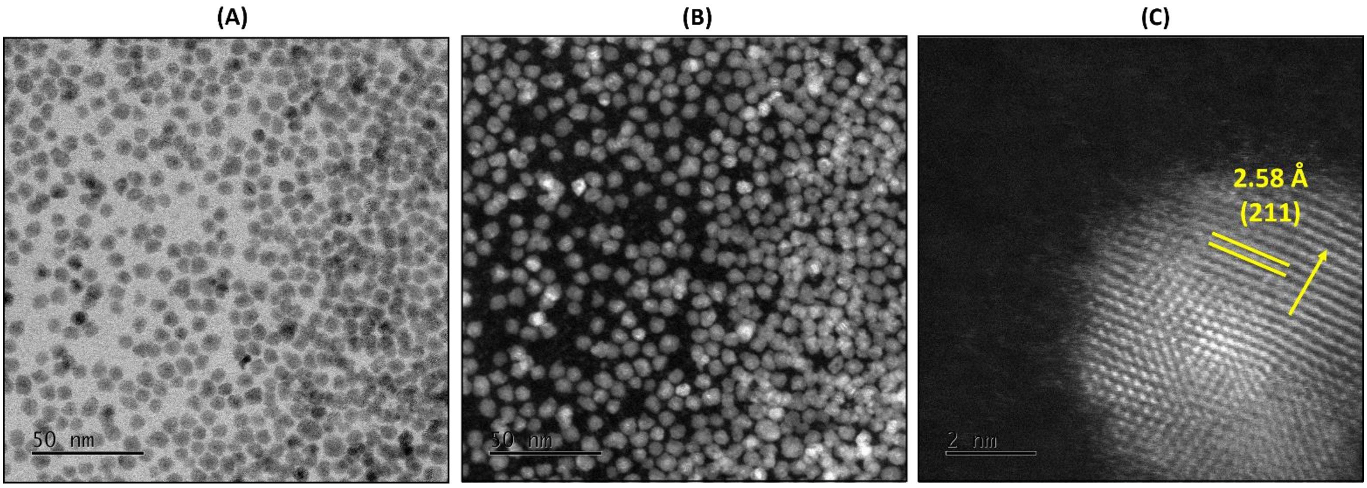

Figure S2. (A) Annular bright field STEM image, **(B)** Annular dark field STEM image and **(C)** HR-TEM image with lattice fringes and multiple grain boundaries shown for Cu_2O@(MEEA)(OH) (**1-OH**).

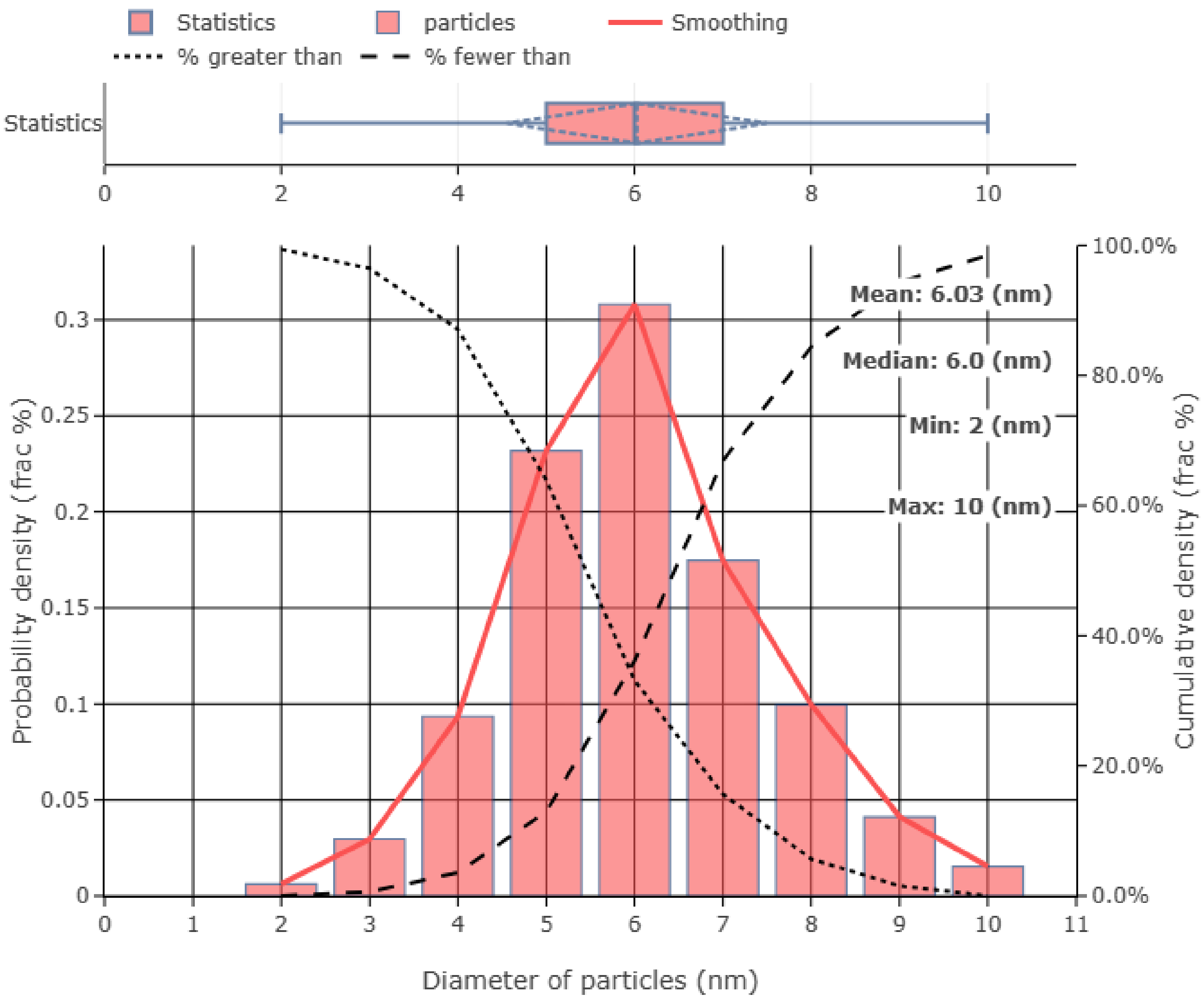

Figure S3. Size distribution histogram obtained from the TEM data for Cu_2O@(MEEA)(OH) (**1-OH**).

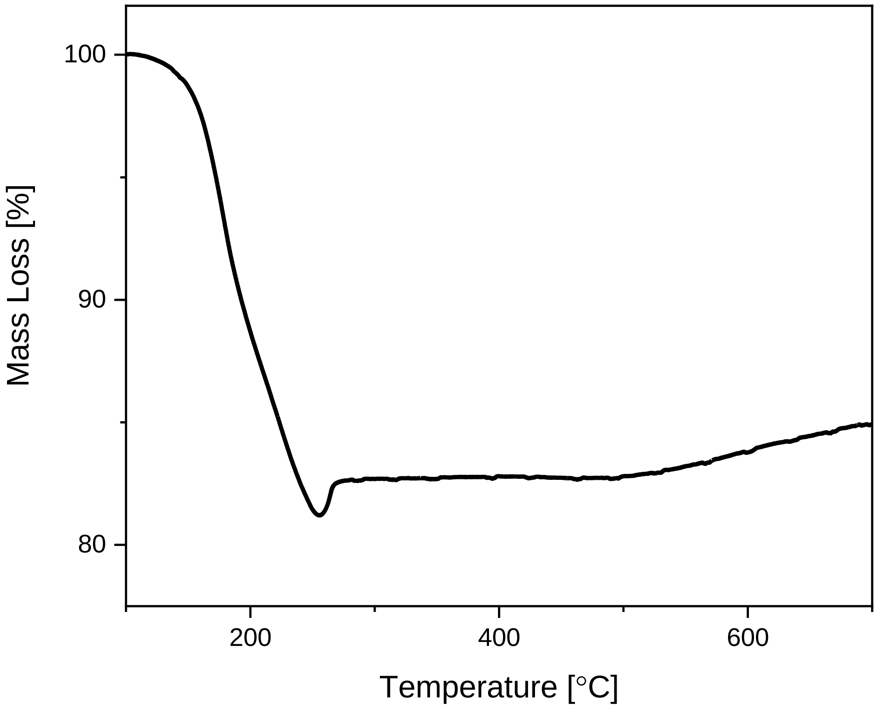

Figure S4. TGA thermogram of Cu_2O@(MEEA)(OH) (**1-OH**) in air. Observed mass loss = 19 wt%; theoretical = 24 wt% (for loss of ligand and oxide).[2]

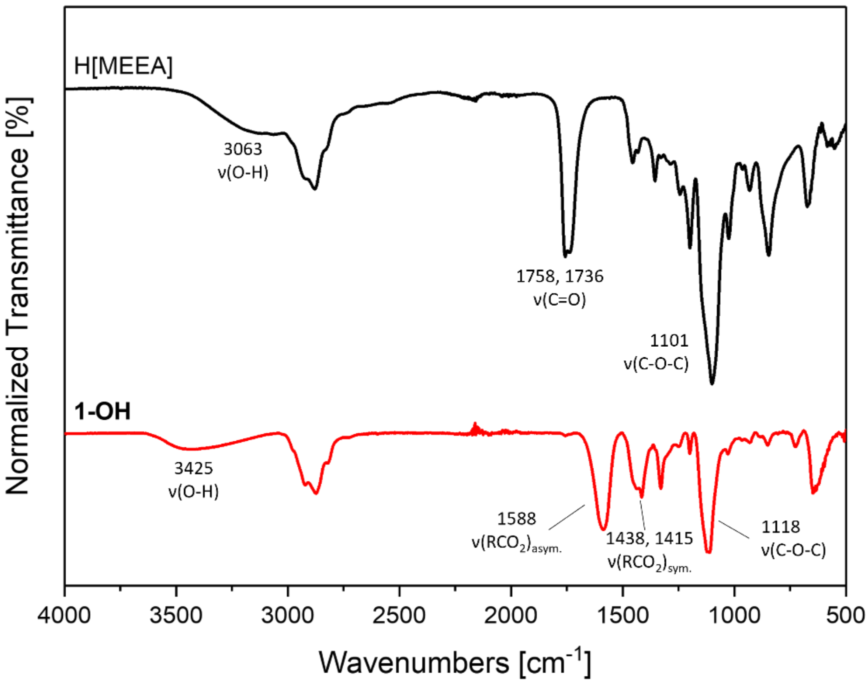

Figure S5. FT-IR spectra of H[MEEA] (black plot) and Cu_2O@(MEEA)(OH) (**1-OH**; red plot). Upon coordination, the free ν(C=O) at 1758/1736 cm^{-1} splits into asymmetric and symmetric carboxylate stretches at 1588 and 1438/1415 cm^{-1}, respectively. Further, the band located at 3425 cm^{-1} in the FT-IR spectrum of **1-OH** is shifted relative to ν(O-H) of H[MEEA] (3063 cm^{-1}) and does not decrease in intensity when samples of **1-OH** are subjected to dynamic vacuum over time and is observed despite FT-IR spectra being collected on dried samples. Therefore, it has been assigned as ν(O-H) of surface hydroxyl groups, as opposed to H_2O bound to the crystallite surface.[2]

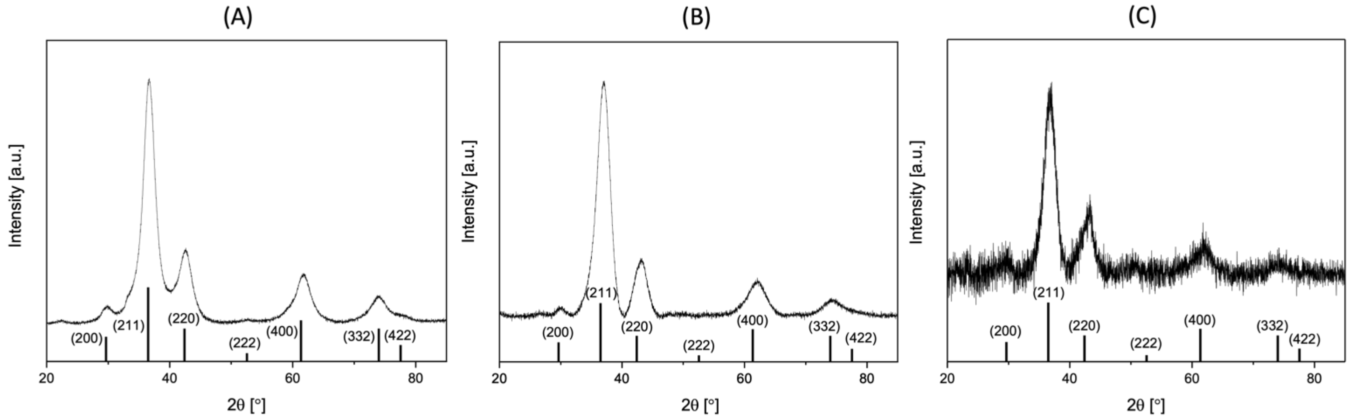

Figure S6. Powder X-ray diffraction pattern of Cu_2O@(MEEA)(OH) (**1-OH**) when synthesized using **(A)** 10 mol%, **(B)** 20 mol% and **(C)** 30 mol% H[MEEA] loading. In all three cases, the crystallites are 3 nm in size (Scherrer analysis). The diffraction pattern is indexed against cubic Cu_2O as vertical bars (JCPDS 00-002-1067). The powder X-ray diffraction data in **(A)** and **(B)** were collected in 12-hour experiments, whereas the data in **(C)** was collected in a 1-hour experiment, resulting in increased signal-to-noise ratio.

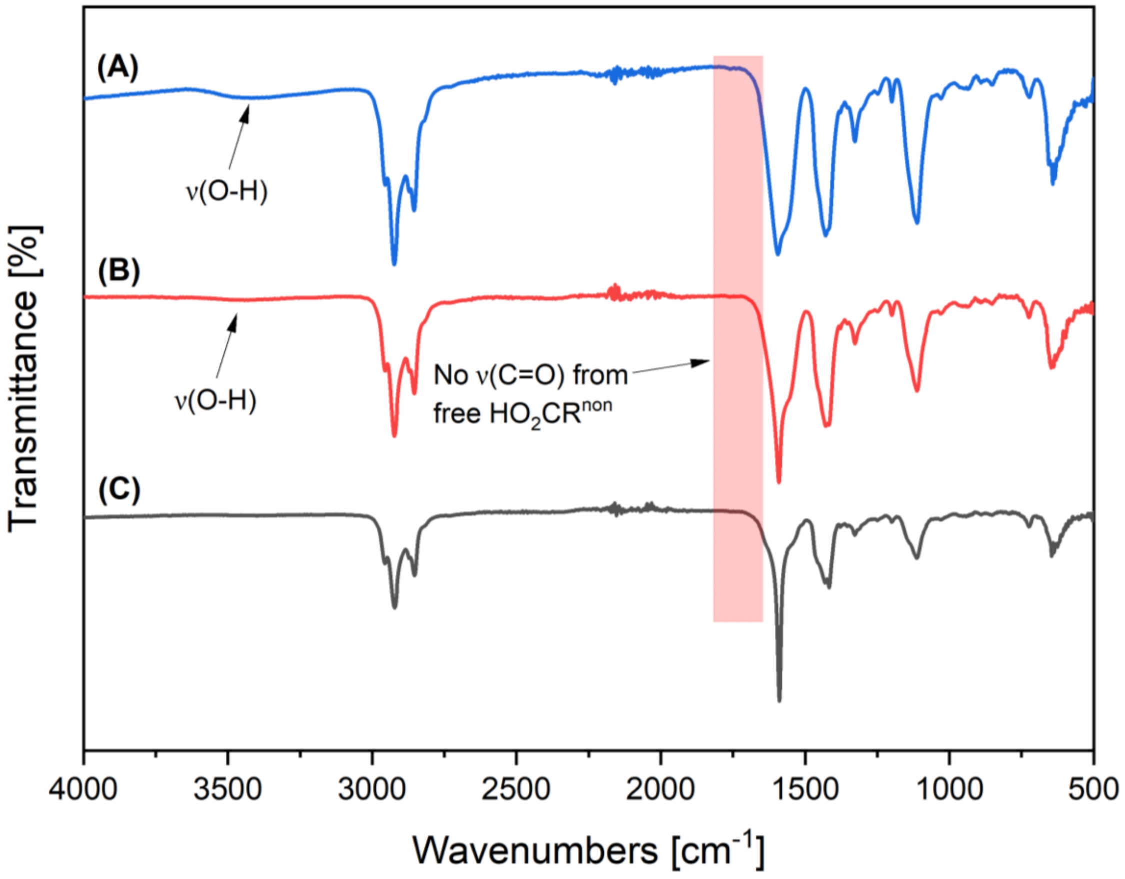

Figure S7. Stacked FT-IR spectra following the addition of **(A)** 10 mol% **(B)** 15 mol% and **(C)** 20 mol% of nonanoic acid (HO_2CR^{non}) to **1-OH**; surface titration experiments used to evaluate the quantity of -OH groups bound to the surface of Cu_2O@(MEEA).

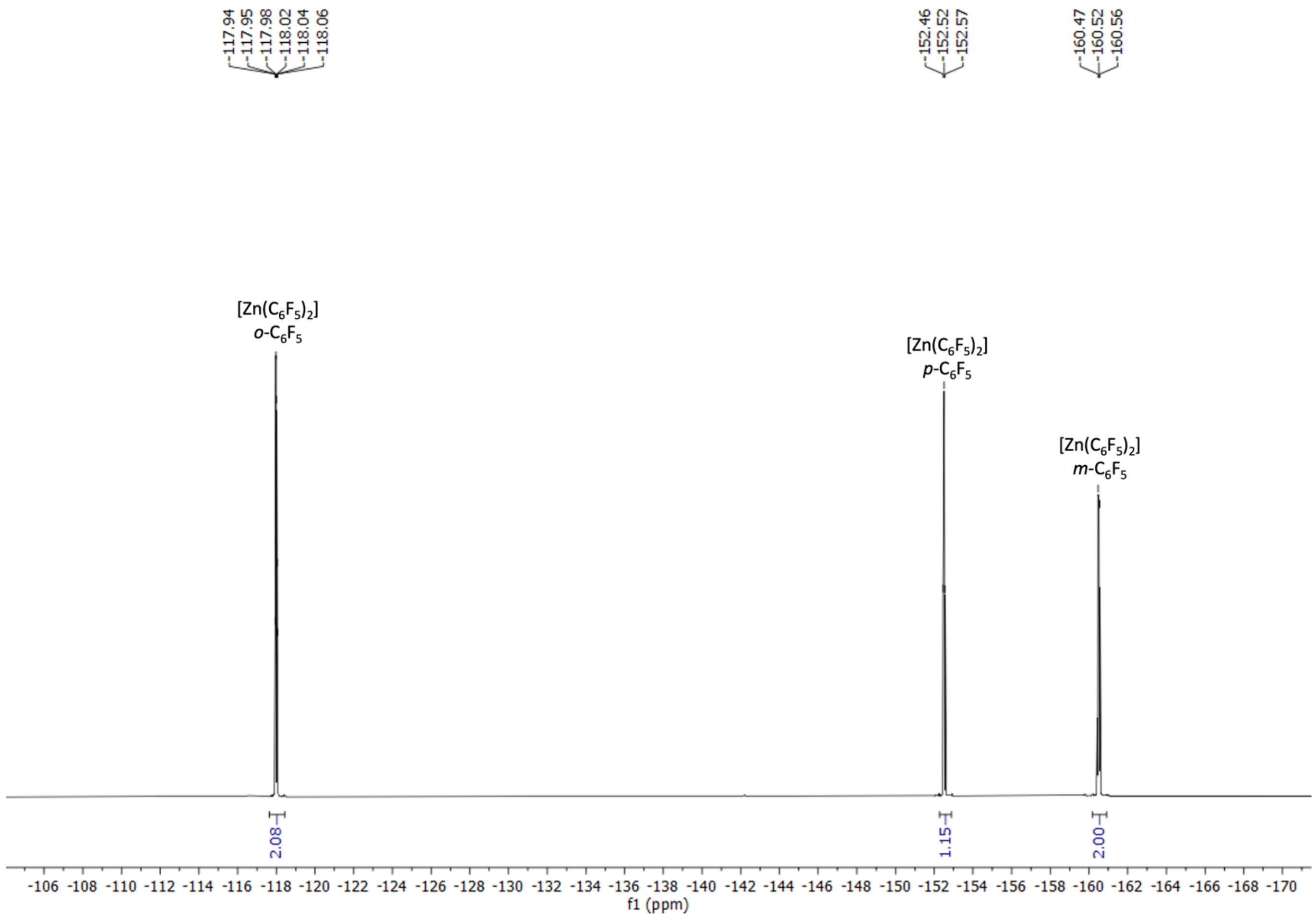

Figure S8. $^{19}F\{^1H\}$ NMR spectrum of $[Zn(C_6F_5)_2]$ (benzene-d_6, 377 MHz, 298 K).

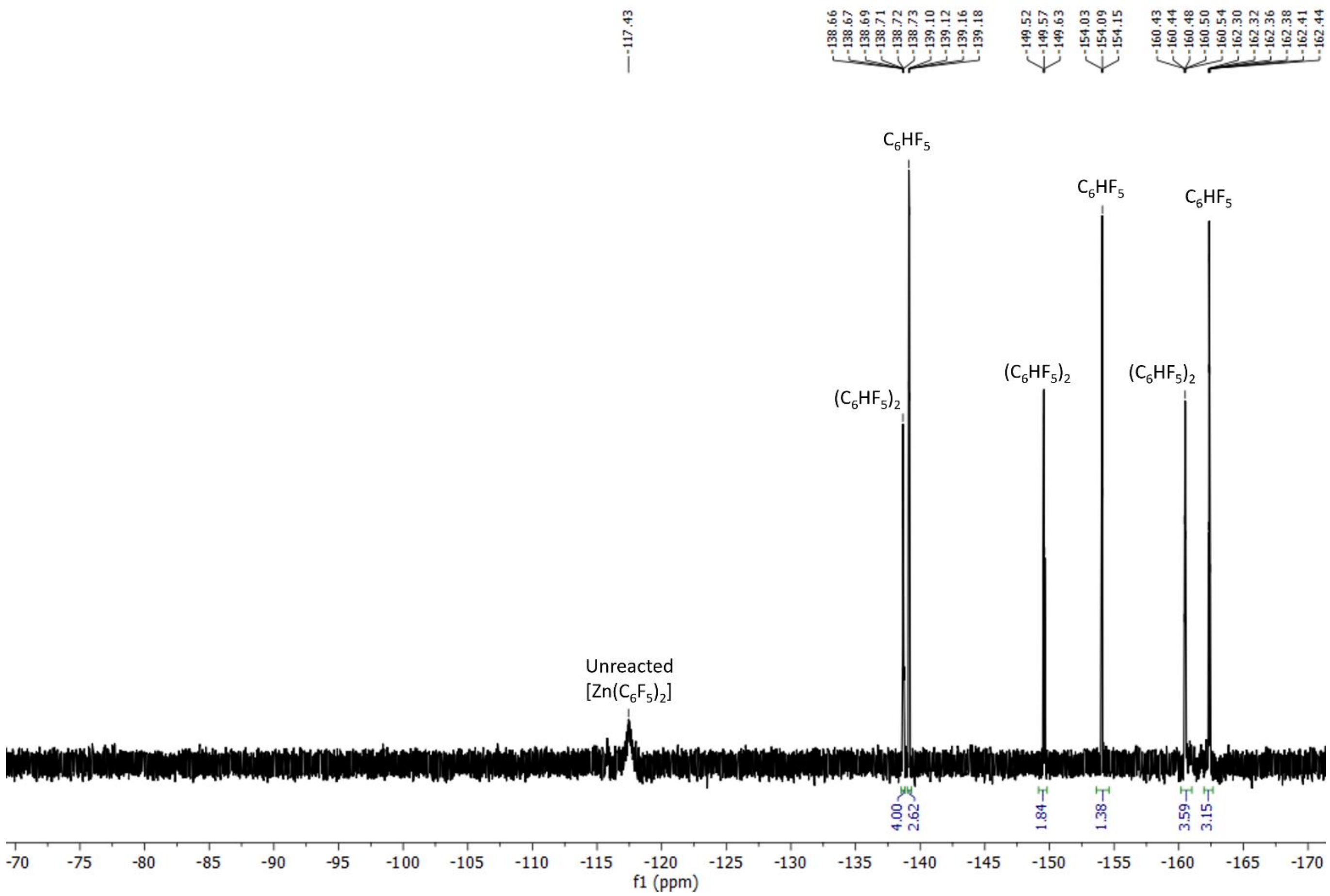

Figure S9. $^{19}F\{^{1}H\}$ NMR spectrum of the crude reaction mixture following the addition of 20 mol% of $[Zn(C_6F_5)_2]$ to **1-OH** (benzene-d_6/toluene, 377 MHz, 298 K). ^{19}F NMR chemical shifts for $[Zn(C_6F_5)_2]$ (377 MHz, benzene-d_6): –118.0, –152.5 and –160.5 ppm (see Figure S8); ^{19}F NMR chemical shifts for C_6HF_5 (188 MHz, benzene-d6): –139.4, –154.0 and –162.7 ppm;[7] ^{19}F NMR chemical shifts for $(C_6F_5)_2$ (377 MHz, $CDCl_3$): –138.6, –150.9 and –161.4 ppm.[8]

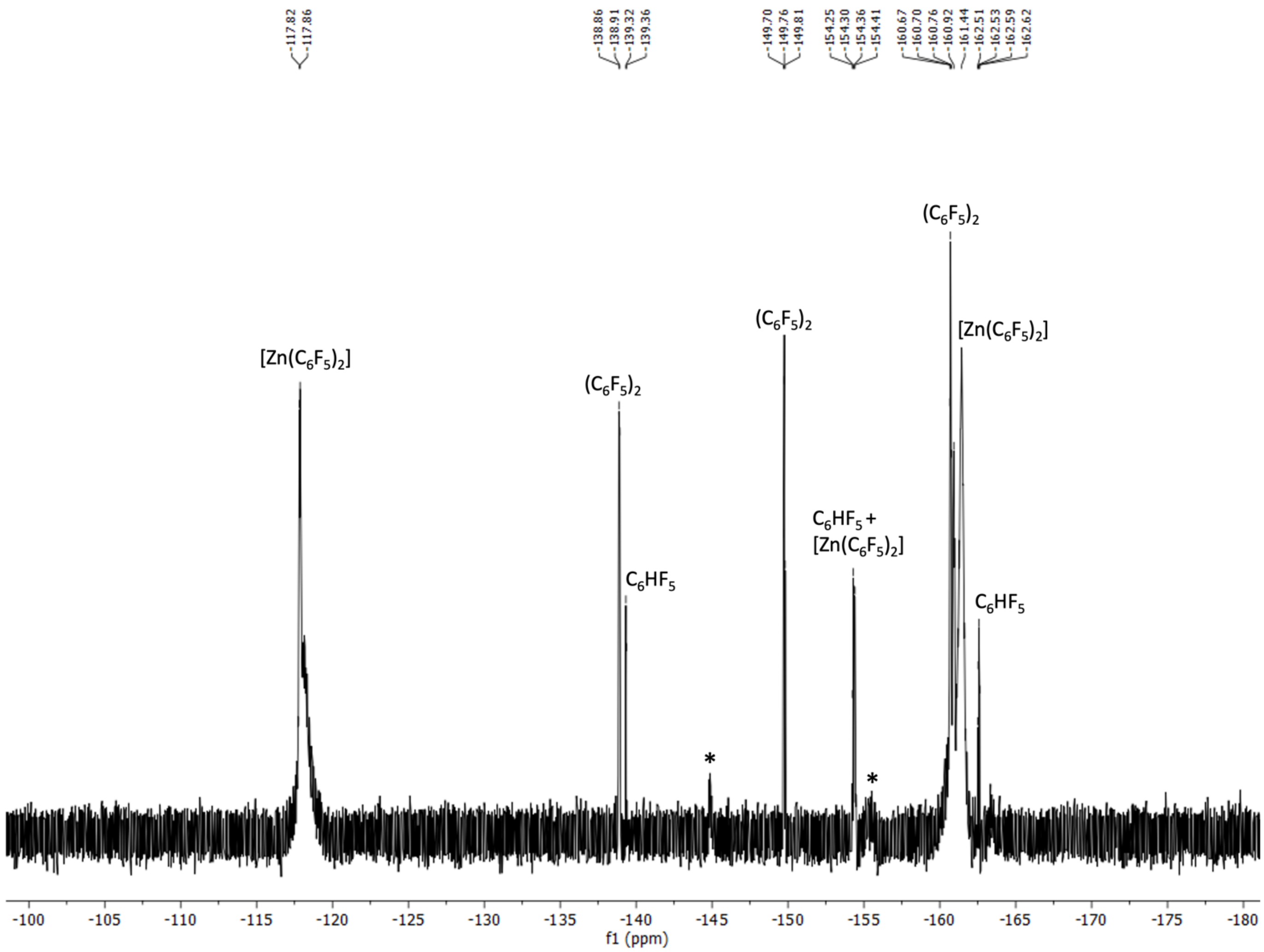

Figure S10. $^{19}F\{^1H\}$ NMR spectrum of the soluble reaction by-products following the addition of 30 mol% of $[Zn(C_6F_5)_2]$ to **1-OH** (benzene-d_6/toluene, 377 MHz, 298 K). Unreacted $[Zn(C_6F_5)_2]$ is now observable, indicating 20 mol% is the optimal quantity for nanocrystal surface decoration. * Represent unidentified reaction by-products; since the majority product is insoluble, these side products represent a very small fraction of the whole. ^{19}F NMR chemical shifts for $[Zn(C_6F_5)_2]$ (377 MHz, benzene-d_6): –118.0, –152.5 and –160.5 ppm (see Figure S8); ^{19}F chemical shifts for C_6HF_5 (188 MHz, benzene-d6): –139.4, –154.0 and –162.7 ppm;[7] ^{19}F chemical shifts for $(C_6F_5)_2$ (376 MHz, $CDCl_3$): –138.6, –150.9 and –161.4 ppm.[8]

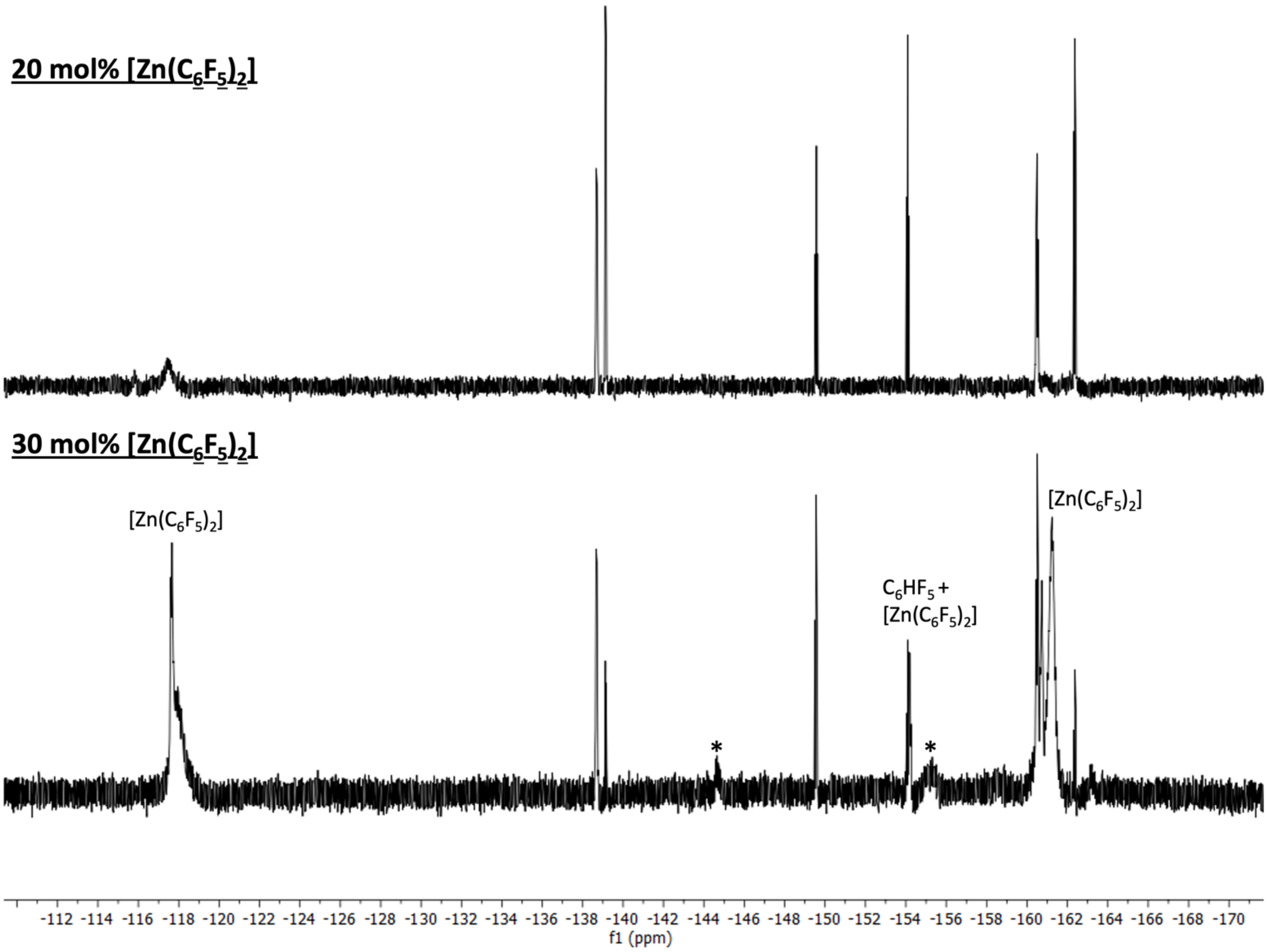

Figure S11. Stacked $^{19}F\{^{1}H\}$ NMR spectra of the soluble reaction by-products following the addition of 20 and 30 mol% of $[Zn(C_6F_5)_2]$ to **1-OH** (benzene-d_6/toluene, 377 MHz, 298 K). Unreacted $[Zn(C_6F_5)_2]$ is observable in the 30 mol% reaction, indicating 20 mol% $[Zn(C_6F_5)_2]$ is the optimal quantity for nanocrystal surface decoration. * Represent unidentified reaction by-products; since the majority product is insoluble, these side products represent a very small fraction of the whole. ^{19}F NMR chemical shifts for $[Zn(C_6F_5)_2]$ (377 MHz, benzene-d_6): –118.0, –152.5 and –160.5 ppm (see Figure S8); ^{19}F chemical shifts for C_6HF_5 (188 MHz, benzene-d_6): –139.4, –154.0 and –162.7 ppm;[7] ^{19}F chemical shifts for $(C_6F_5)_2$ (376 MHz, $CDCl_3$): –138.6, –150.9 and –161.4 ppm.[8]

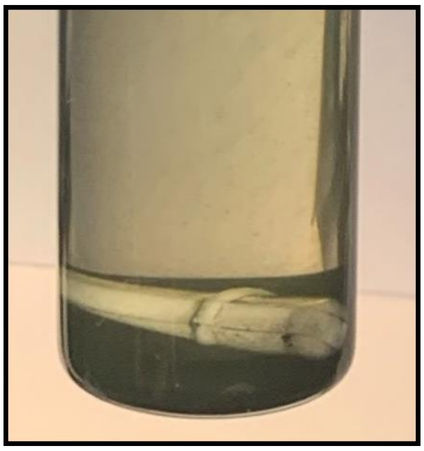

Figure S12. Photograph of **1-OZn(C_6F_5)**, which precipitates from toluene as a dark green powder.

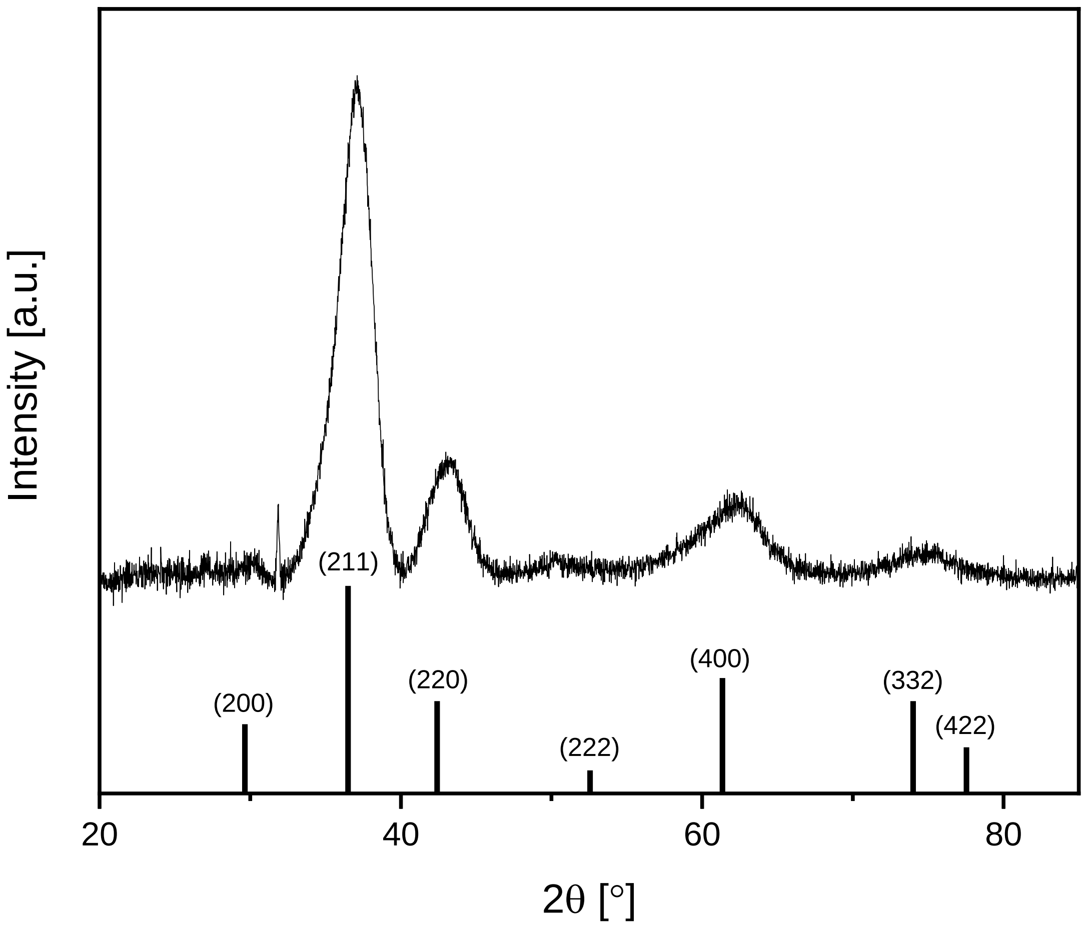

Figure S13. Powder X-ray diffraction pattern of **1-OZn(C_6F_5)**. Pattern indexed against cubic Cu_2O as vertical bars (JCPDS 00-002-1067). Average crystallite size = 3 nm (Scherrer analysis).

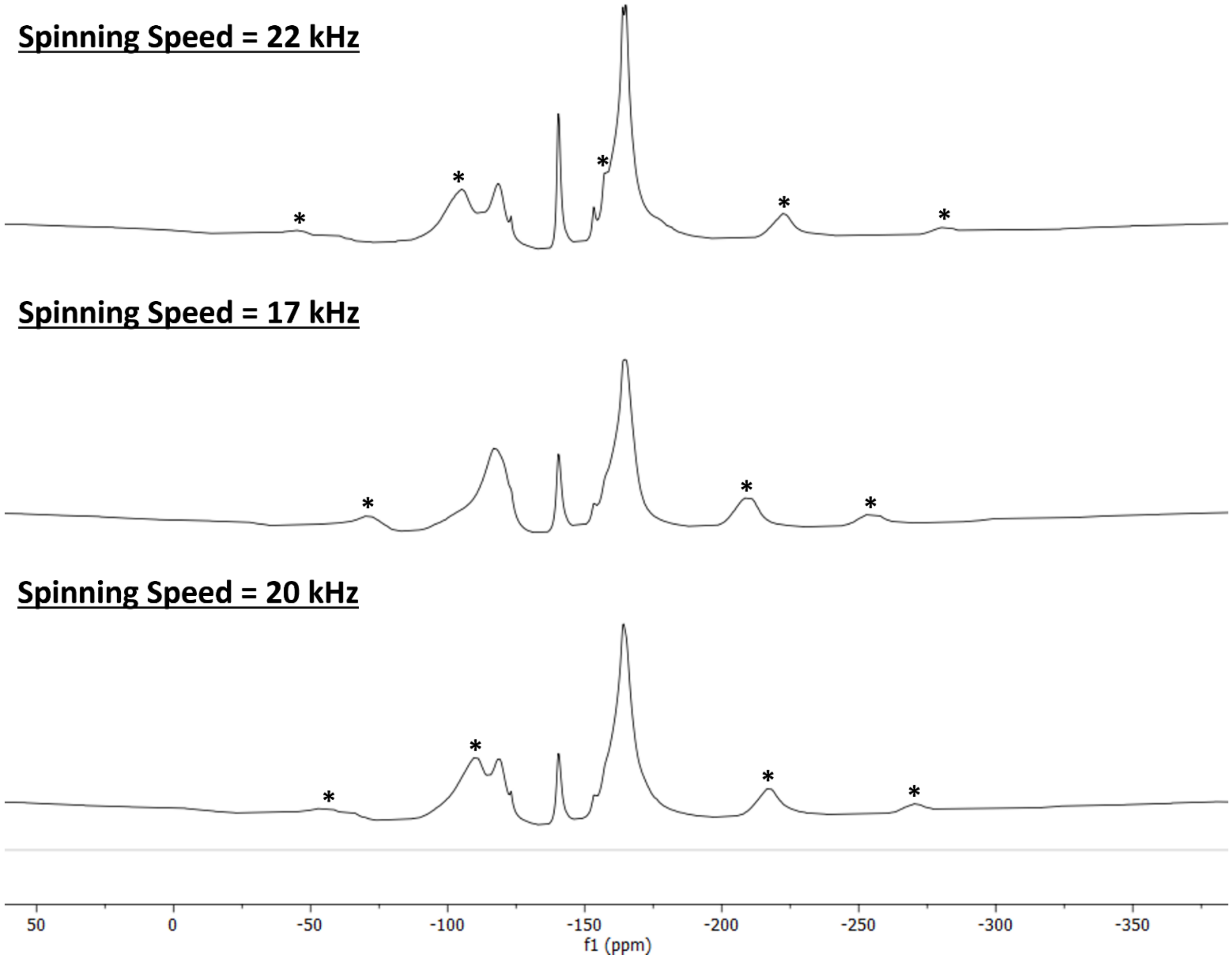

Figure S14. Stacked solid-state $^{19}F\{^{1}H\}$ NMR spectra of isolated **1-OZn(C_6F_5)** at 3 different spinning speeds to locate the spinning side bands (400 MHz, 298 K). * = spinning side band.

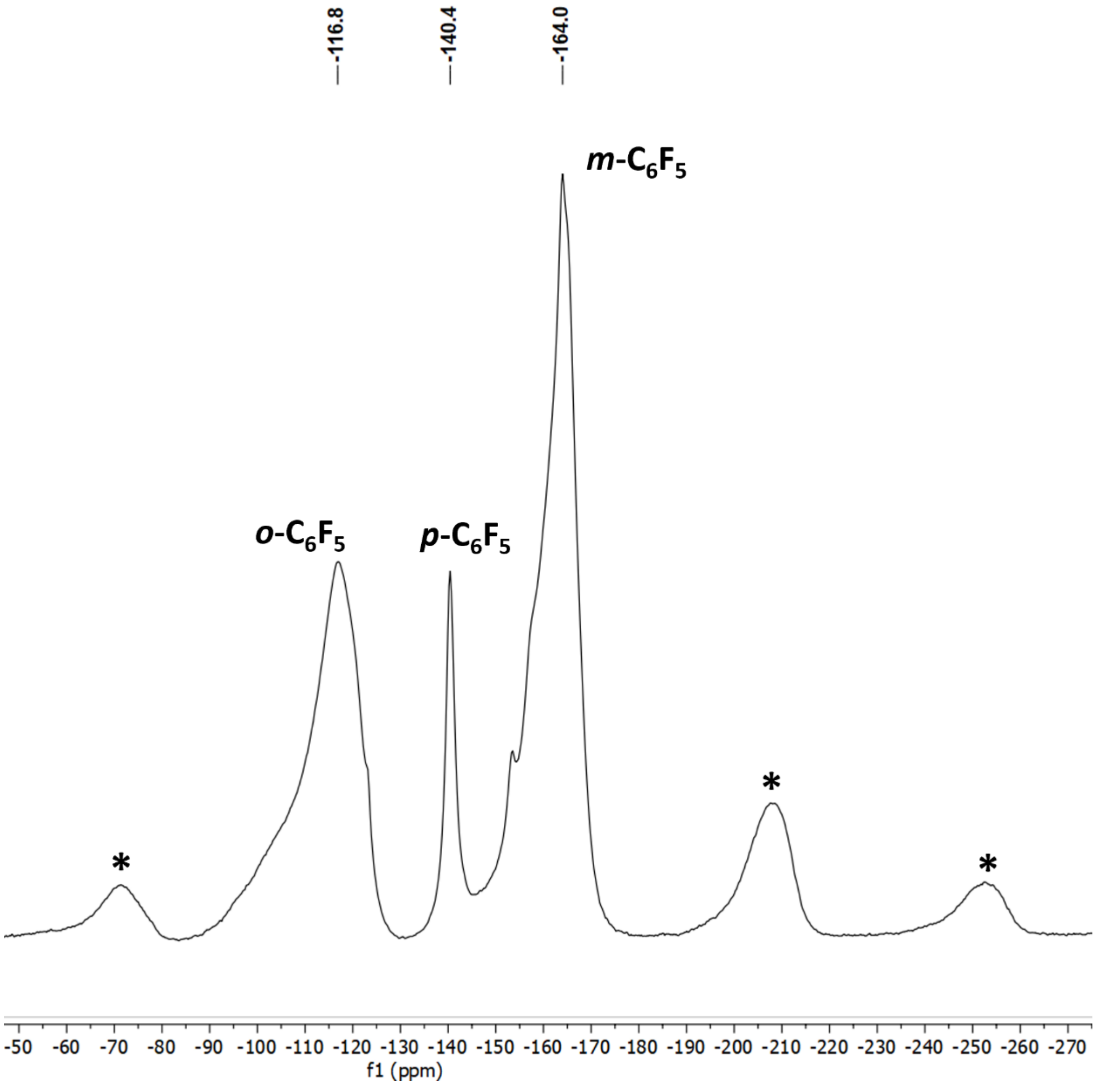

Figure S15. Solid-state $^{19}F\{^{1}H\}$ NMR spectrum of isolated **1-OZn(C_6F_5)** (400 MHz, 298 K, 17 kHz). * = spinning side band.

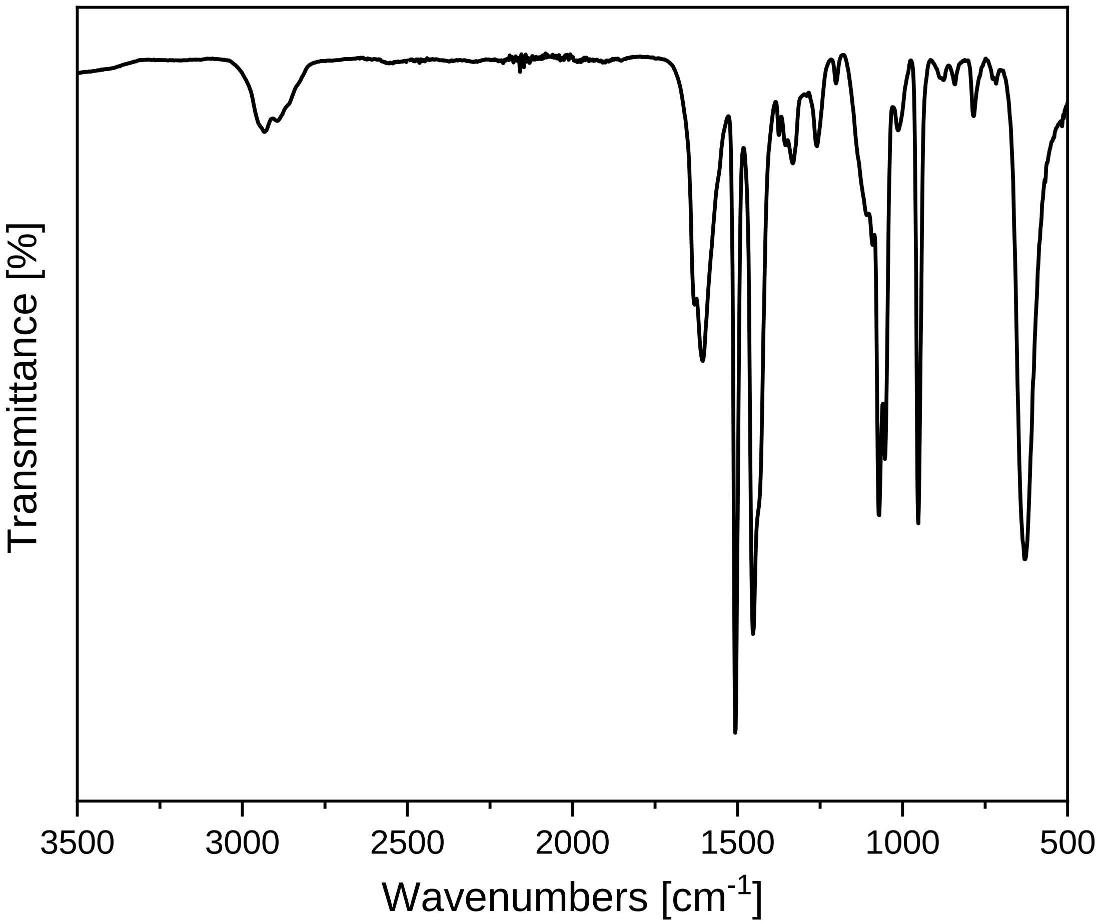

Figure S16. FT-IR spectrum of **1-OZn(C_6F_5)**.

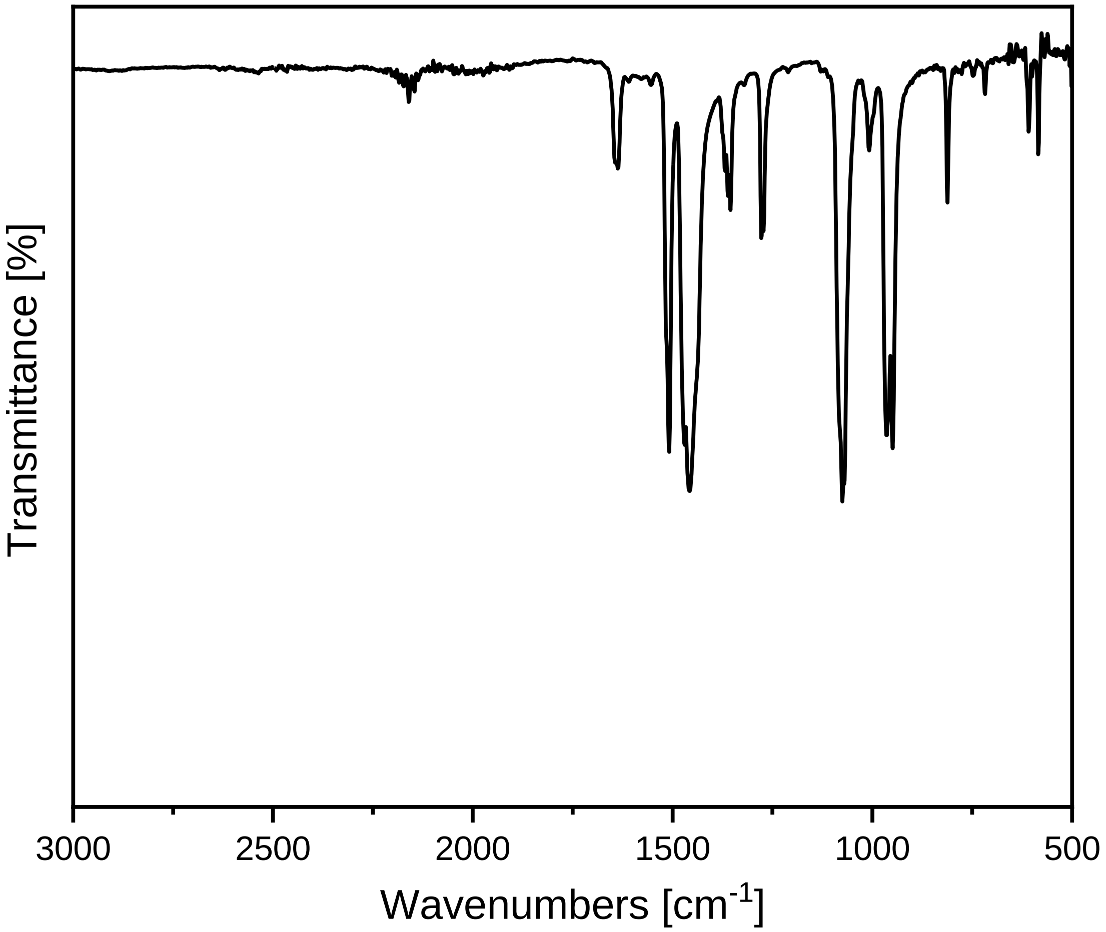

Figure S17. FT-IR spectrum of [Zn(C_6F_5)$_2$].

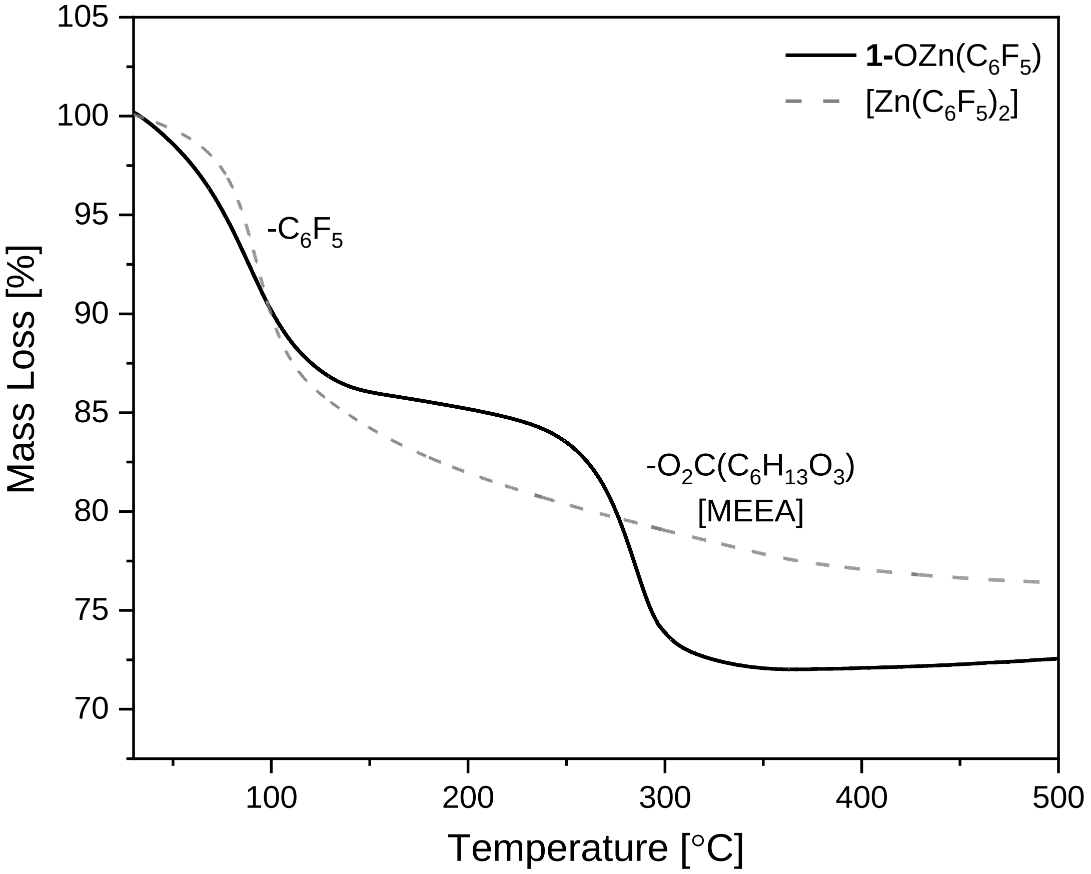

Figure S18. TGA thermograms for **1-OZn(C_6F_5)** (solid black line) and [Zn(C_6F_5)$_2$] (dashed grey line) for comparison; loss of -C_6F_5 is observed from ~30-200 °C for both.

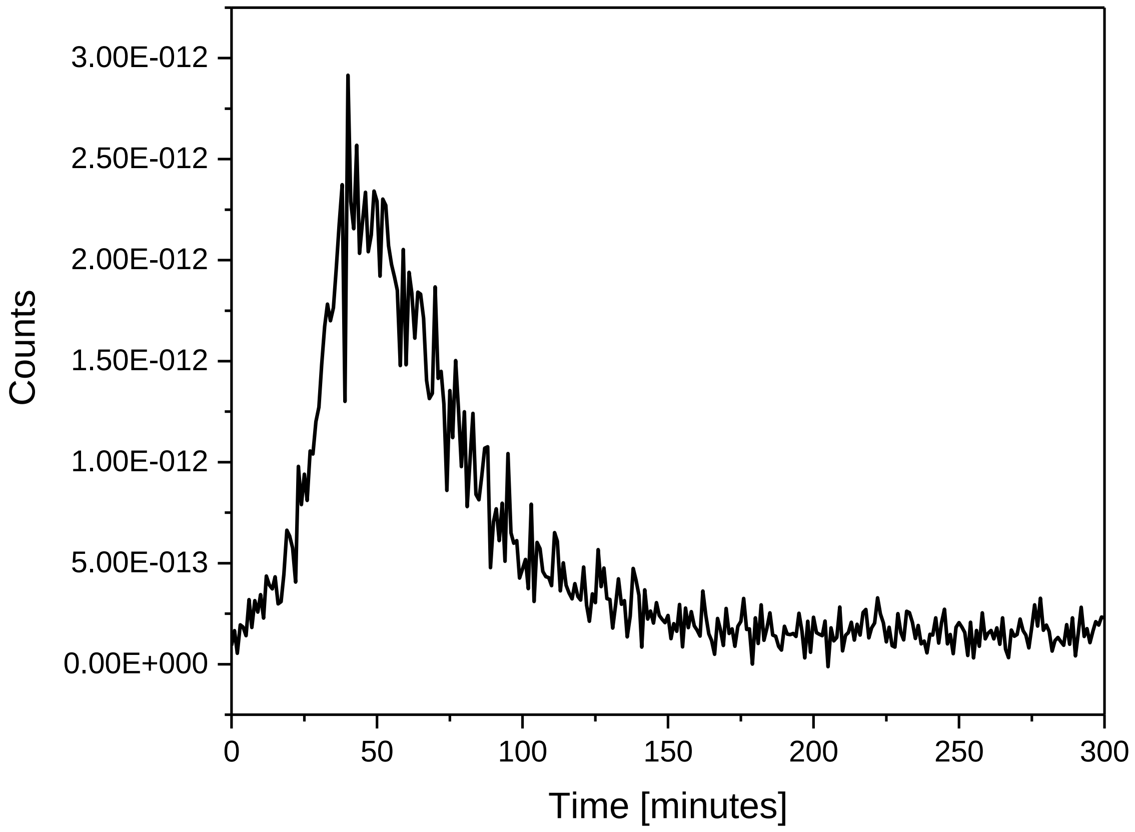

Figure S19. Counts vs. time for the m/z = 168 ion (C_6F_5 + 1) for **1-OZn(C_6F_5)**, obtained by TGA-MS. Heating sequence: Segment 1 = ramp from 20 to 100 °C at 2 °C/minute (40 minutes); Segment 2 = isotherm at 100 °C for 15 minutes; Segment 3 = ramp from 100 to 600 °C at 2 °C/minute.

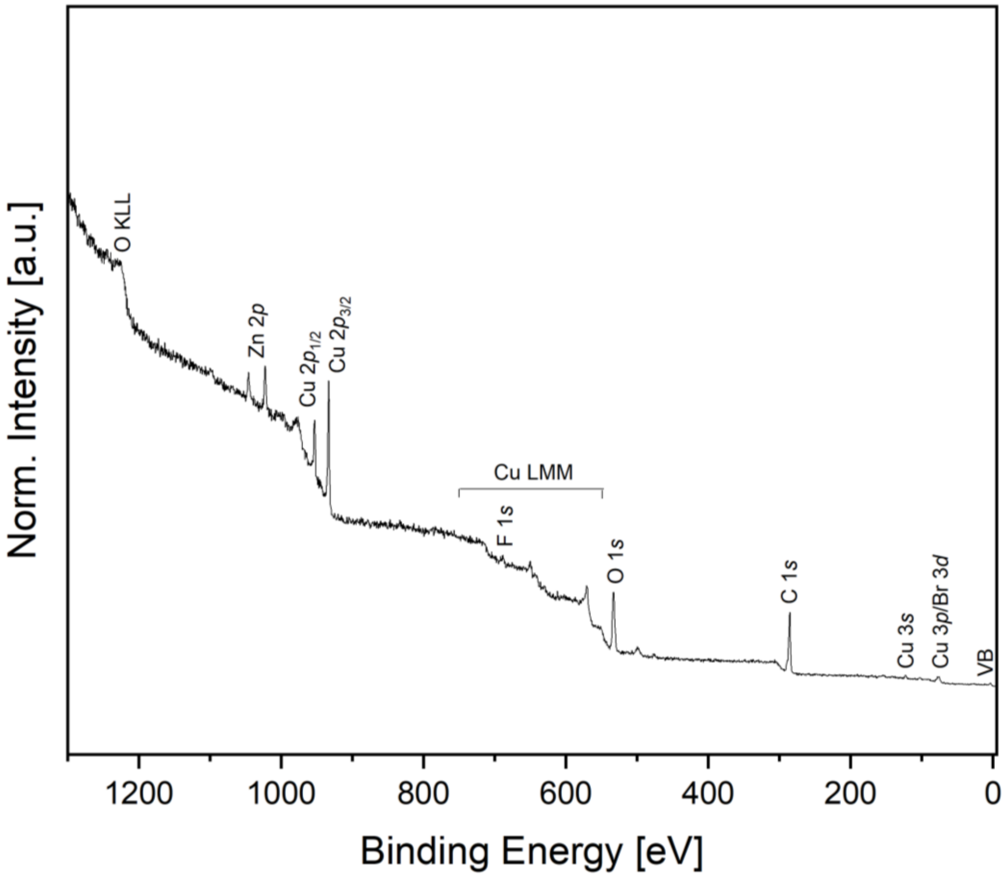

Figure S20. Survey XP spectrum of **1-OZn(C_6F_5)**.

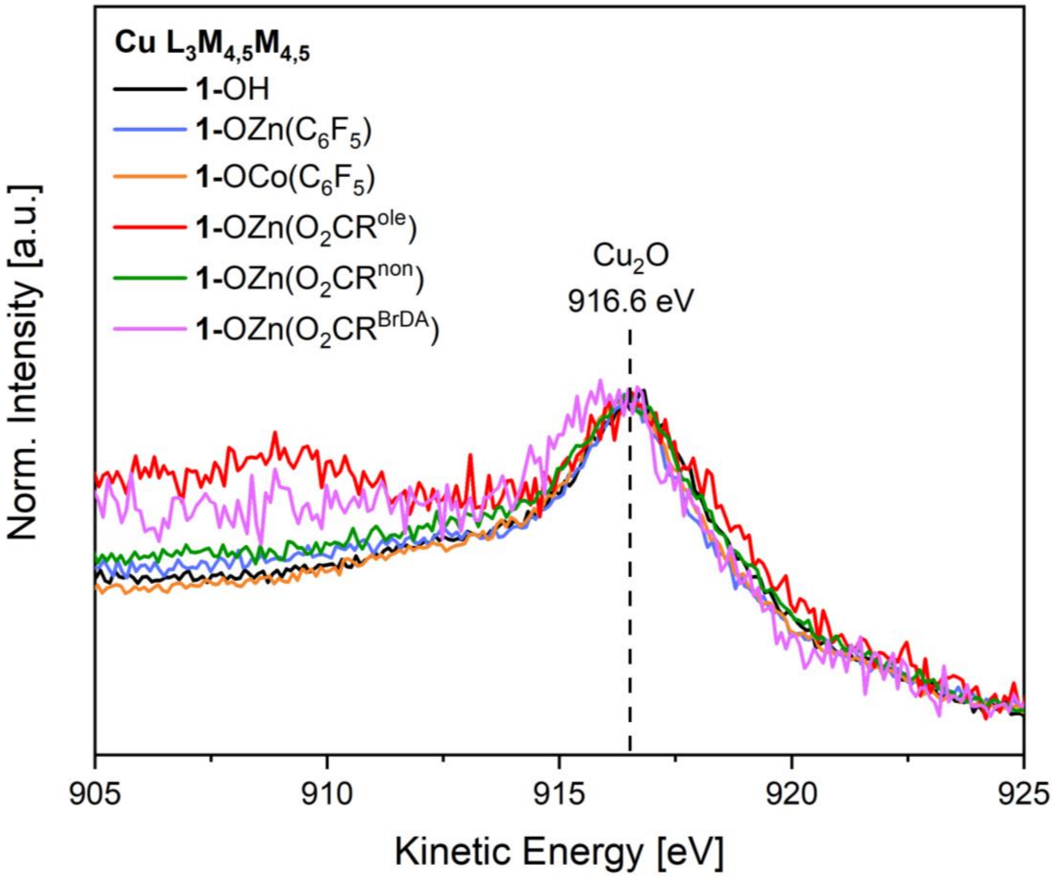

Figure S21. Cu $L_3M_{4,5}M_{4,5}$ Auger XP spectrum of **1-OH**, 1**-OZn(C_6F_5)**, **1-Co(C_6F_5)** and **1-OZn(O_2CR')** (R' = ole, non, BrDA).[9-12]

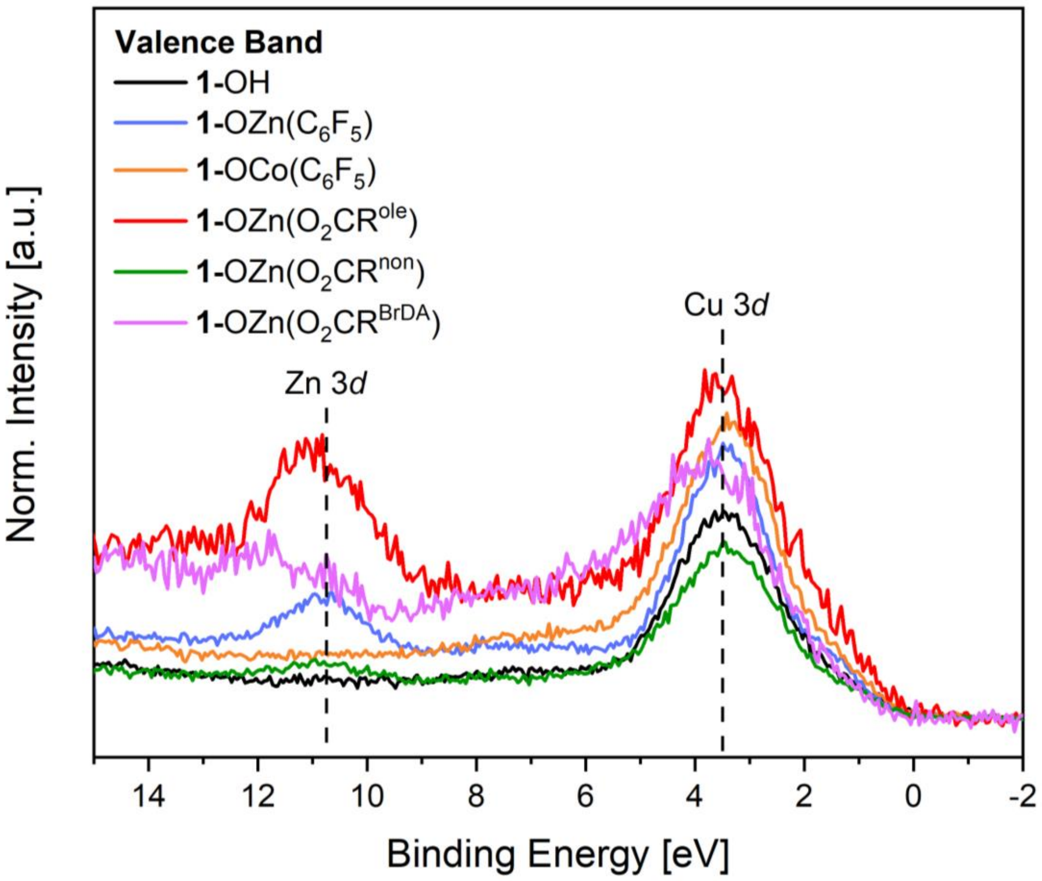

Figure S22. Valence band XP spectrum of **1-OH**, **1-OZn(C_6F_5)**, **1-Co(C_6F_5)** and **1-OZn(O_2CR')** (R' = ole, non, BrDA).[13]

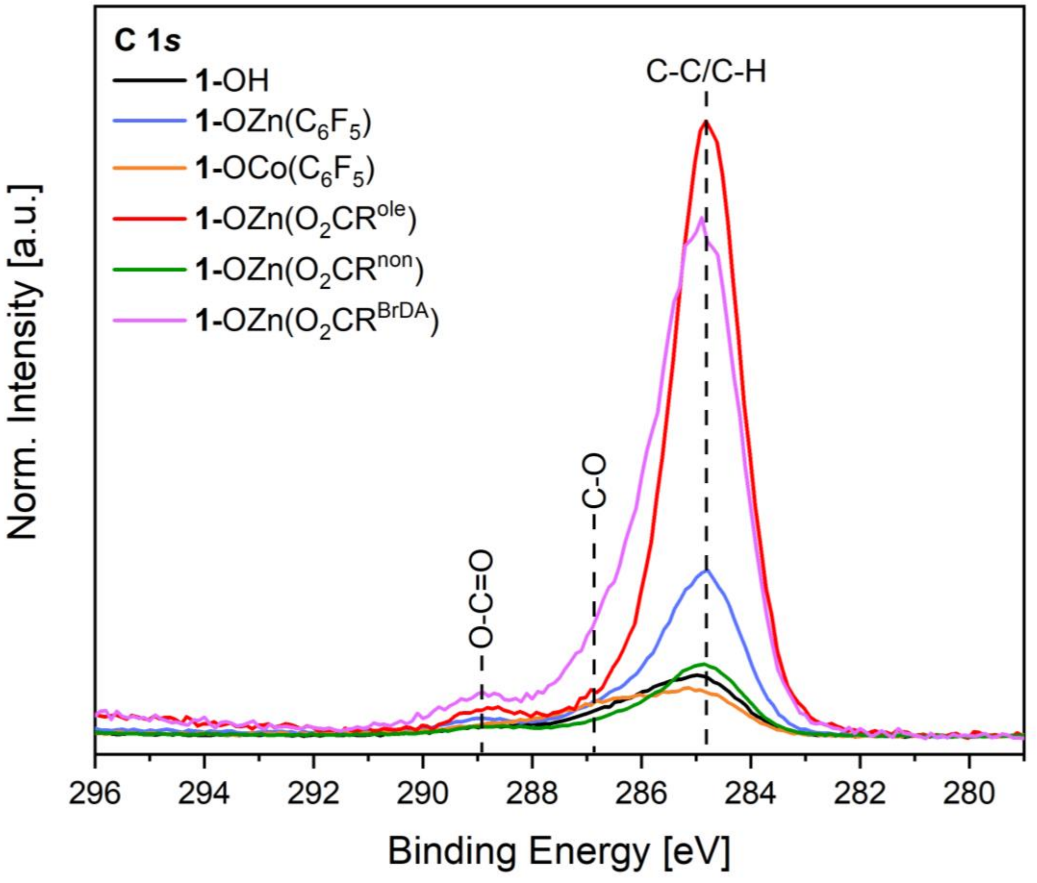

Figure S23. C 1*s* XP spectrum of **1-OH**, **1-OZn(C_6F_5)**, **1-Co(C_6F_5)** and **1-OZn(O_2CR')** (R' = ole, non, BrDA).[14,15]

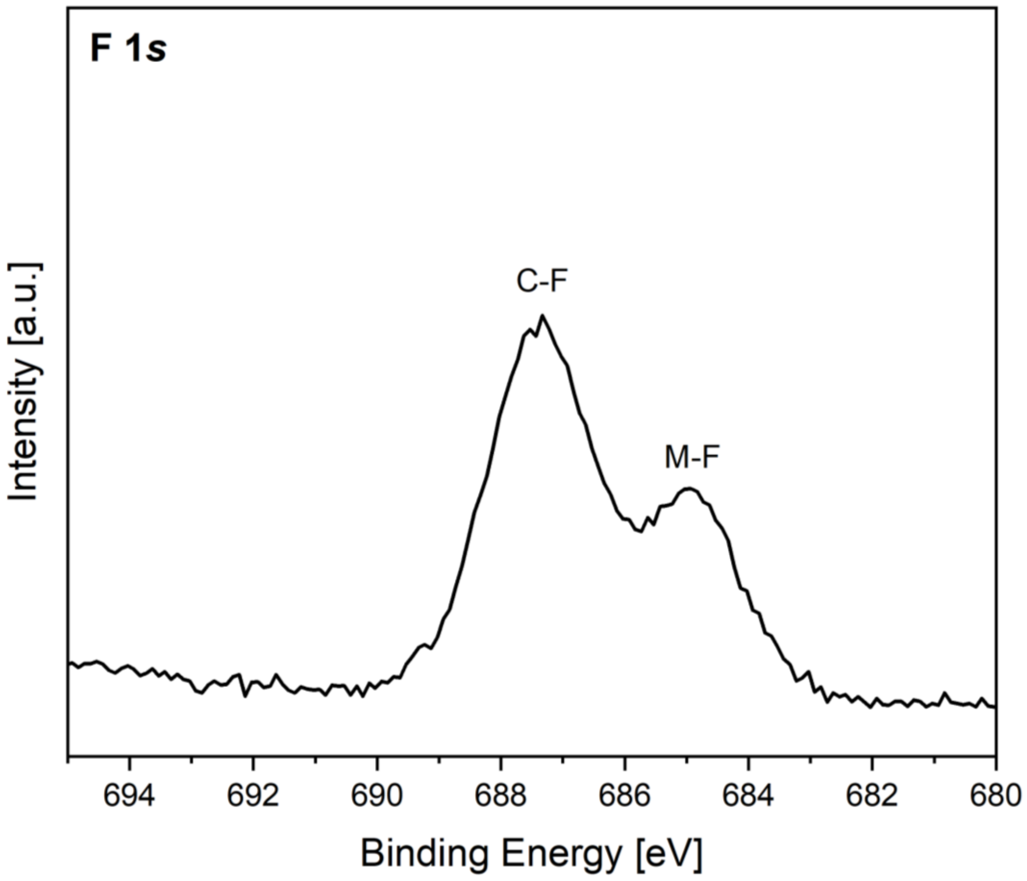

Figure S24. F 1*s* XP spectrum of [Zn(C_6F_5)$_2$].[16-18]

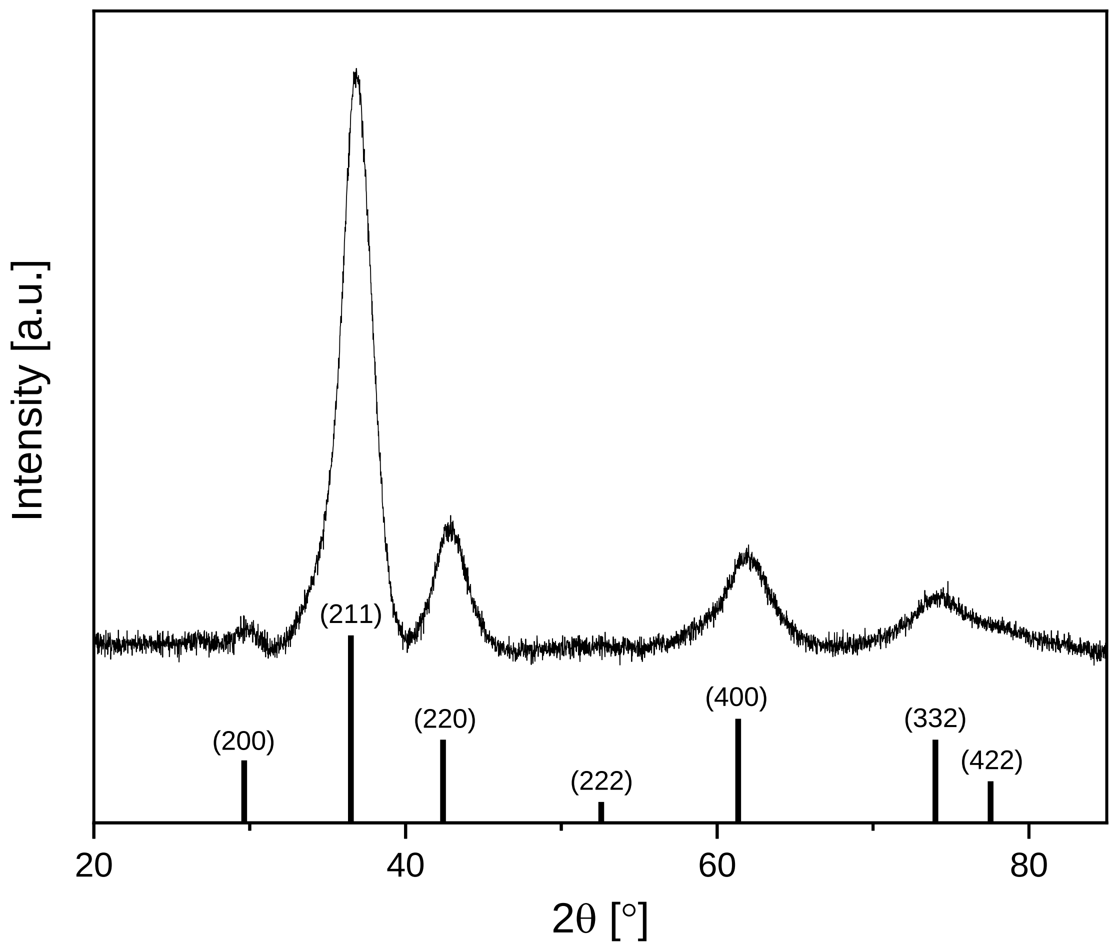

Figure S25. Powder X-ray diffraction pattern of **1-OCo(C_6F_5)**. Pattern indexed against cubic Cu_2O as vertical bars (JCPDS 00-002-1067). Average crystallite size = 3 nm (Scherrer analysis).

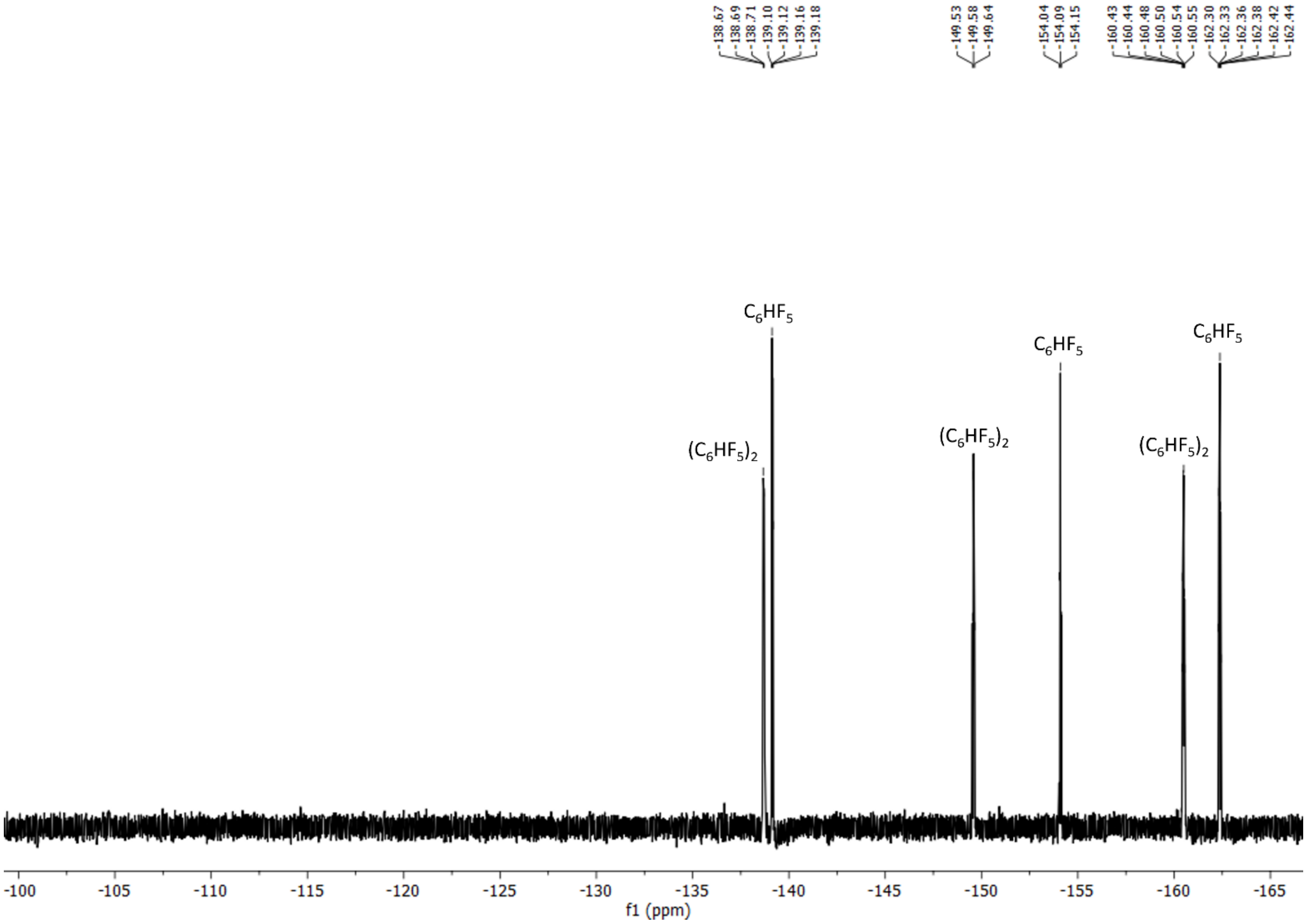

Figure S26. $^{19}F\{^1H\}$ NMR spectrum of the crude reaction mixture following the addition of 20 mol% of $[Co(C_6F_5)_2]\cdot 2THF$ to **1-OH** (benzene-d_6/toluene, 377 MHz, 298 K). ^{19}F NMR chemical shifts for $[Zn(C_6F_5)_2]$ (377 MHz, benzene-d_6): –118.0, –152.5 and –160.5 ppm (see Figure S8); ^{19}F chemical shifts for C_6HF_5 (188 MHz, benzene-d6): –139.4, –154.0 and –162.7 ppm;[7] ^{19}F chemical shifts for $(C_6F_5)_2$ (376 MHz, $CDCl_3$): –138.6, –150.9 and –161.4 ppm.[8]

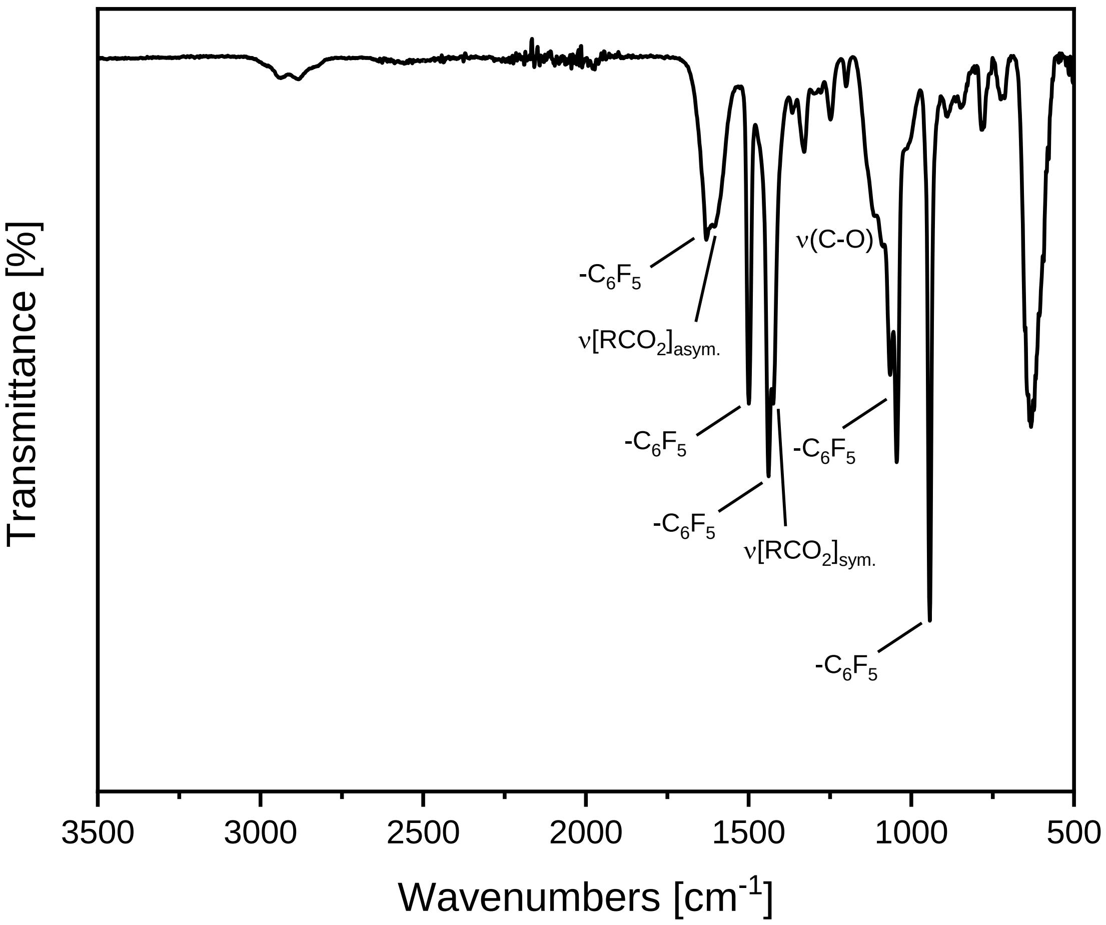

Figure S27. FT-IR spectrum of **1-OCo(C_6F_5)**.

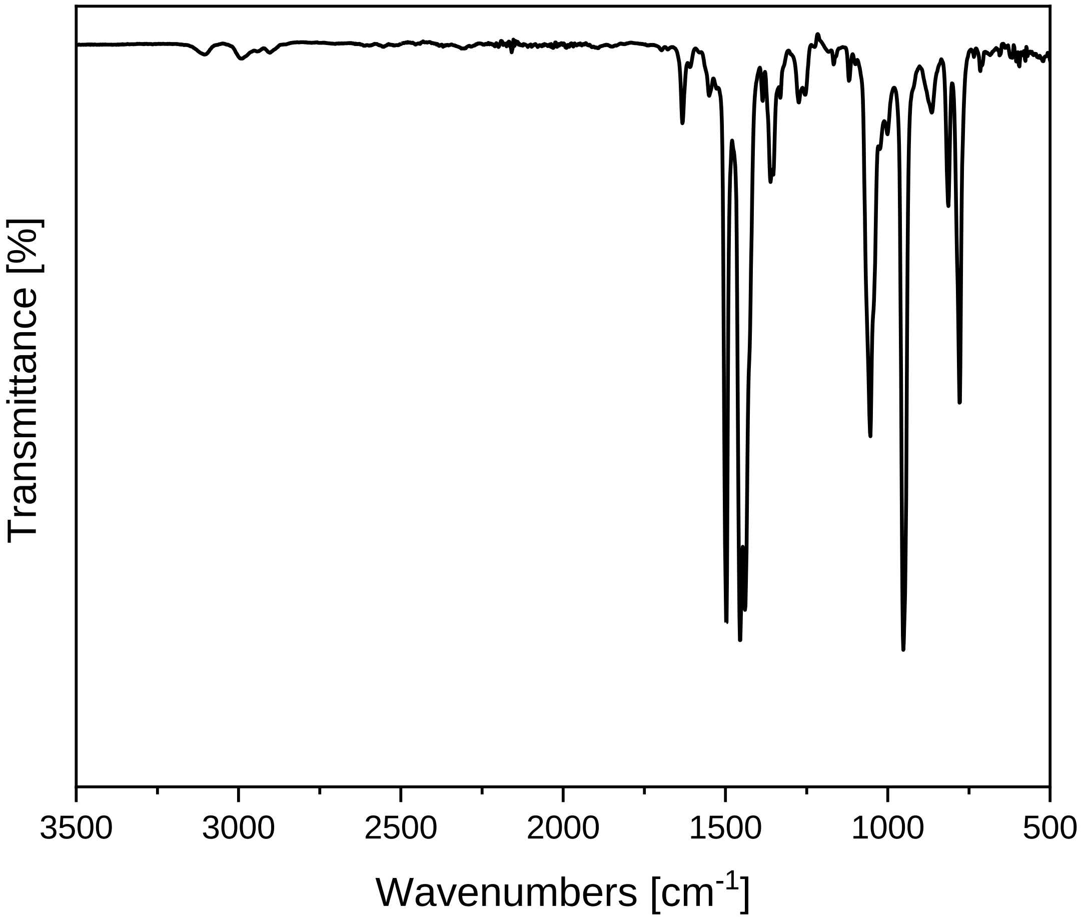

Figure S28. FT-IR spectrum of $[Co(C_6F_5)_2]\cdot 2THF$.

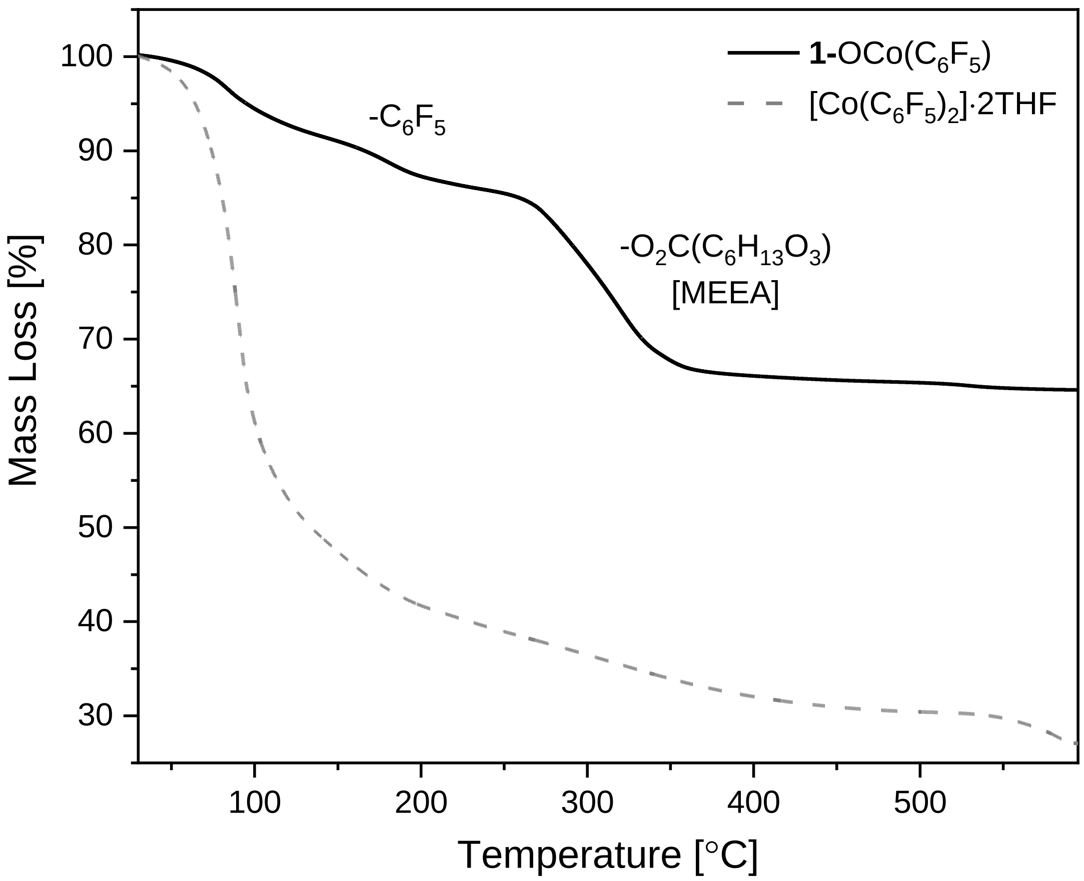

Figure S29. TGA thermogram of **1-OCo(C_6F_5)**, with comparison to [Co(C_6F_5)$_2$]·2THF. The expected organic mass loss, assuming the remaining material is Cu_2O/CoO, is 32 % (observed = 33 %).

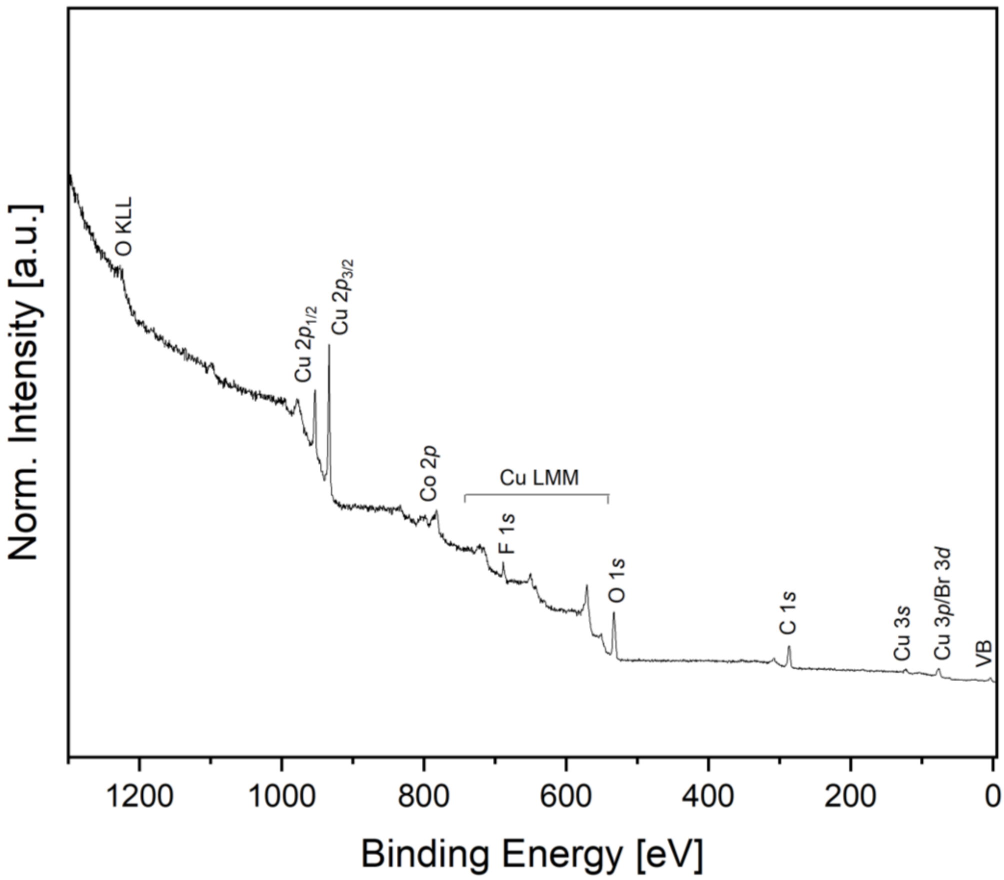

Figure S30. Survey XP spectrum of **1-OCo(C_6F_5)**.

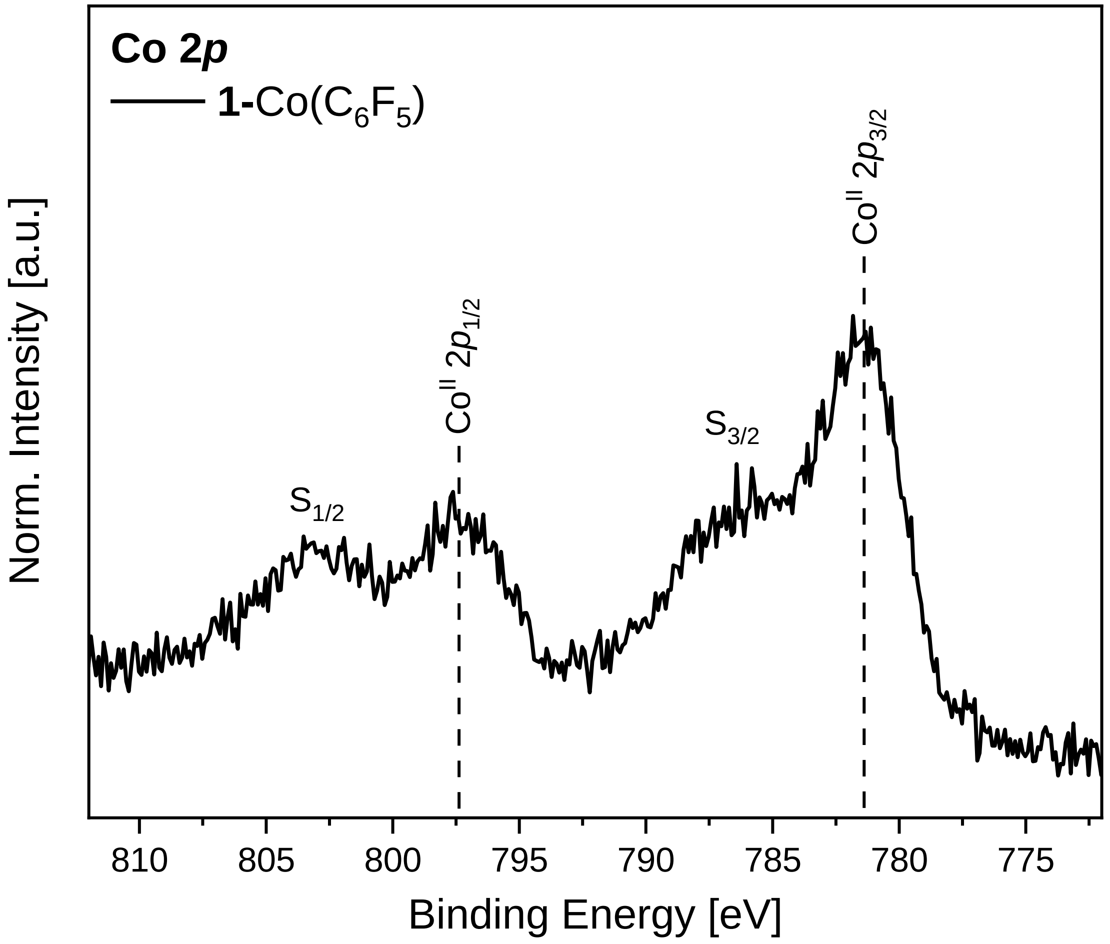

Figure S31. Co 2*p* XP spectrum of **1-Co(C_6F_5)**; the main intensity peaks at 781 and 798 eV are commensurate with the 2+ oxidation state, and satellites ($S_{X/Y}$) on the higher binding energy side of the core lines provides further validation for this assignment.[19]

Table S3. Carboxylic acids and their associated pKa.[20]

Carboxylic Acid	pKa
2-[2-(2-methoxyethoxy)ethoxy]acetic acid (H[MEEA])	3.39 ± 0.1
Oleic Acid (HO_2CR^{ole})	4.78 ± 0.1
Nonanoic Acid (HO_2CR^{non})	4.78 ± 0.1
10-Bromodecanoic Acid (HO_2CR^{BrDA})	4.78 ± 0.1

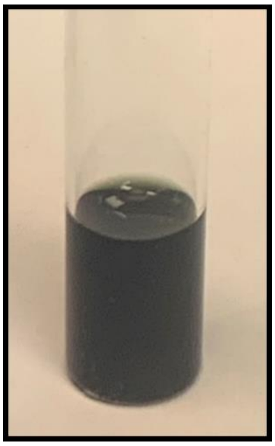

Figure S32. Photograph of **1-OZn(O$_2$CR^{ole})** demonstrating that the addition of HO$_2$CR^{ole} to **1-OZn(C$_6$F$_5$)** results in re-generation of a colloidal solution in toluene.

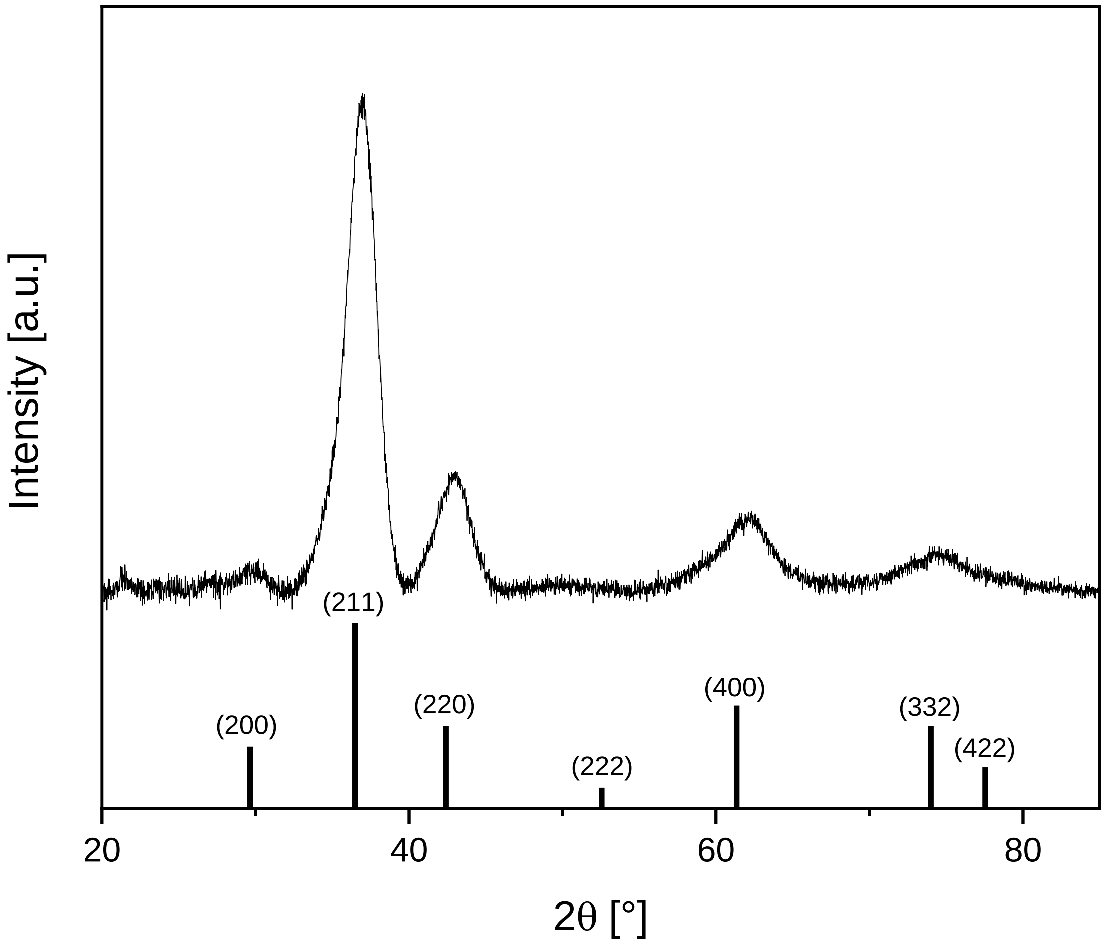

Figure S33. Powder X-ray diffraction pattern of **1-OZn(O$_2$CR^{ole})** (O$_2$CR^{ole} = O$_2$C(CH$_2$)$_7$(CH=CH)(CH$_2$)$_8$CH$_3$; oleate). Pattern indexed against cubic Cu$_2$O as vertical bars (JCPDS 00-002-1067). Average crystallite size = 3 nm (Scherrer analysis).

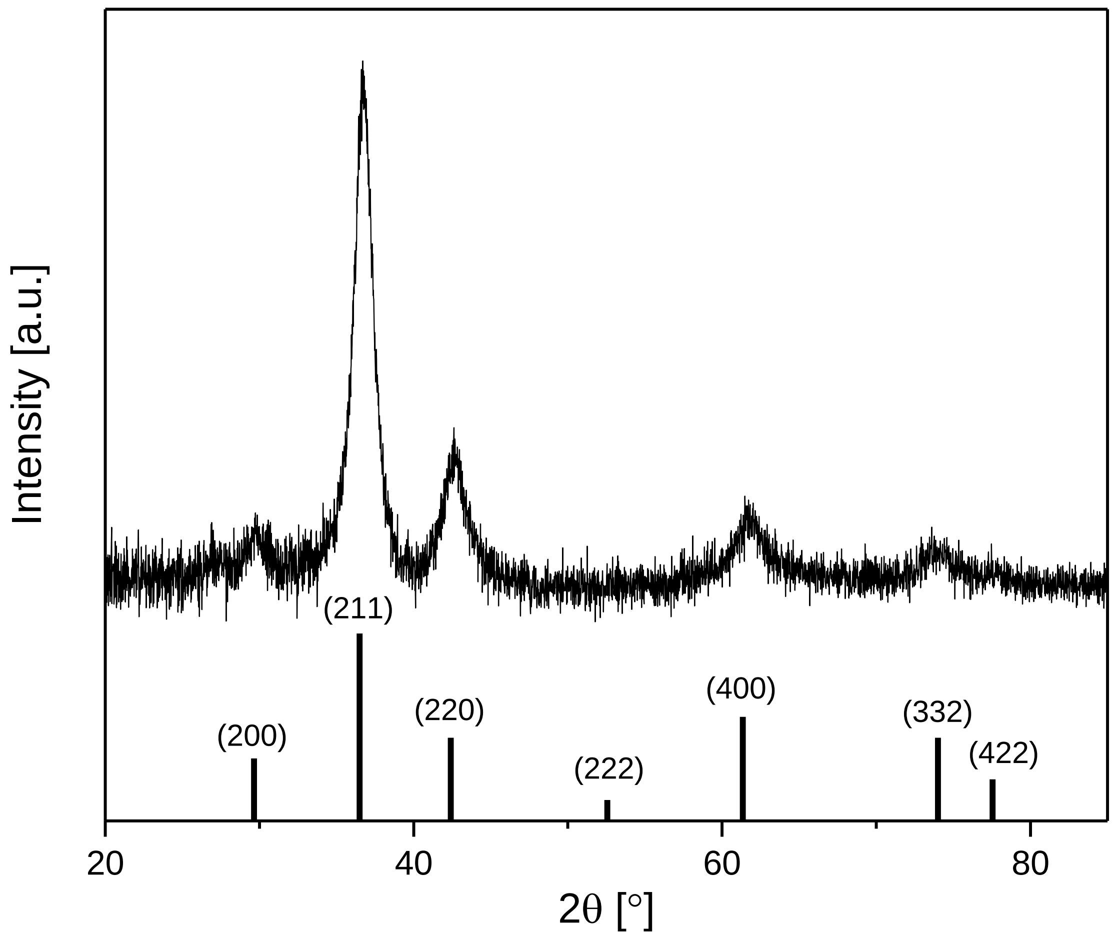

Figure S34. Powder X-ray diffraction pattern of **1-OZn(O_2CR^{non})** (O_2CR^{non} = $O_2C(CH_2)_7CH_3$; nonanoate). Pattern indexed against cubic Cu_2O as vertical bars (JCPDS 00-002-1067). Average crystallite size = 3 nm (Scherrer analysis).

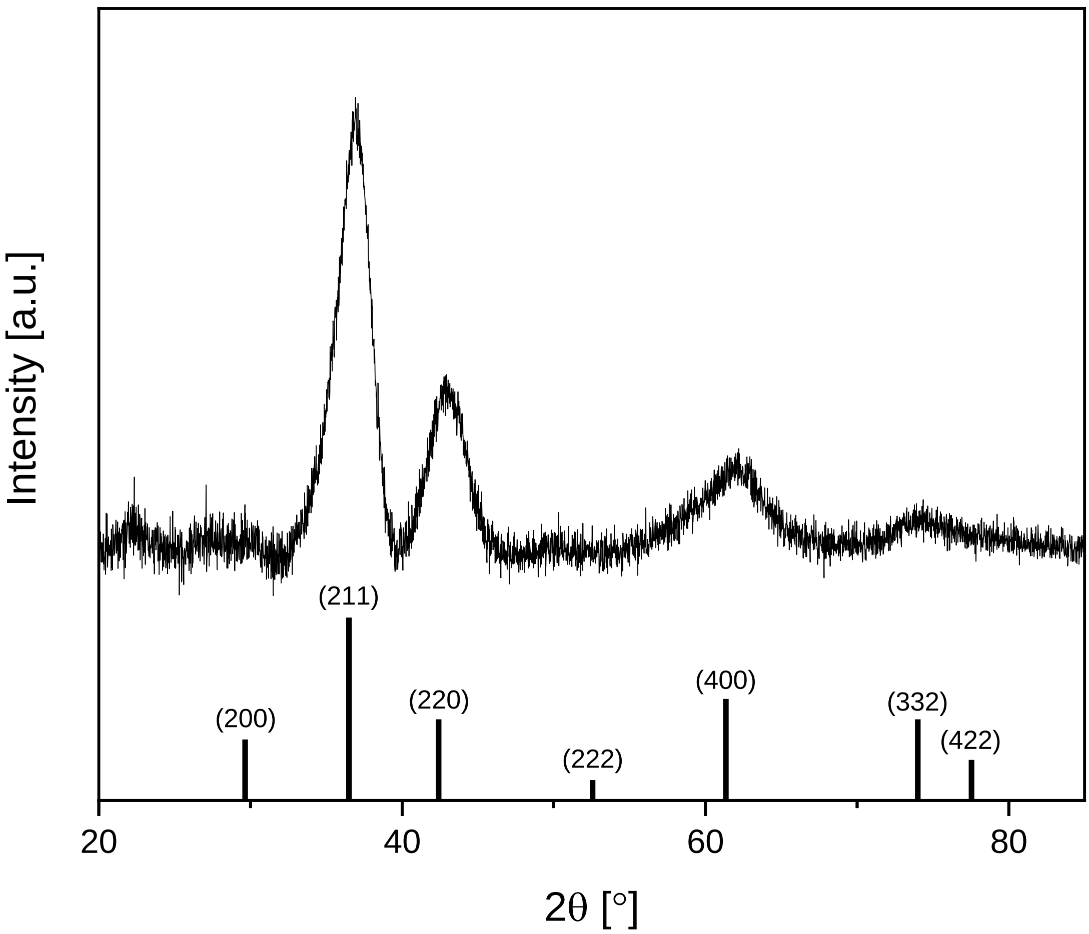

Figure S35. Powder X-ray diffraction pattern of **1-OZn(O_2CR^{BrDA})** (O_2CR^{BrDA} = $O_2C(CH_2)_8CH_2Br$; 10-bromodecanoate). Pattern indexed against cubic Cu_2O as vertical bars (JCPDS 00-002-1067). Average crystallite size = 3 nm (Scherrer analysis).

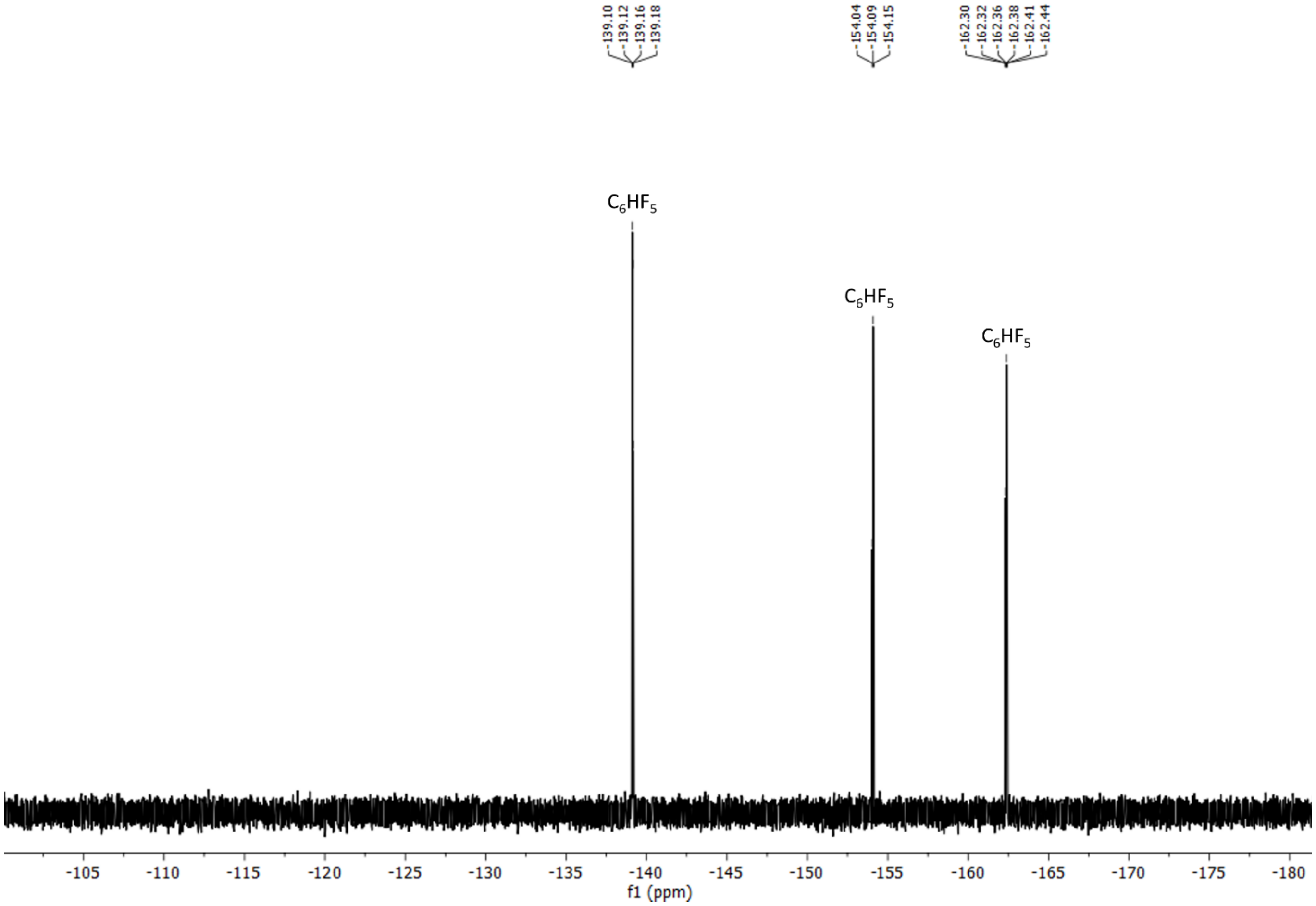

Figure S36. $^{19}F\{^{1}H\}$ NMR spectrum of the crude reaction mixture following the addition of 20 mol% of HO_2CR^{ole} to **1-OZn(C_6F_5)** (benzene-d_6/toluene, 377 MHz, 298 K). ^{19}F chemical shifts for C_6HF_5 (188 MHz, benzene-d_6): –139.4, –154.0 and –162.7 ppm.[7]

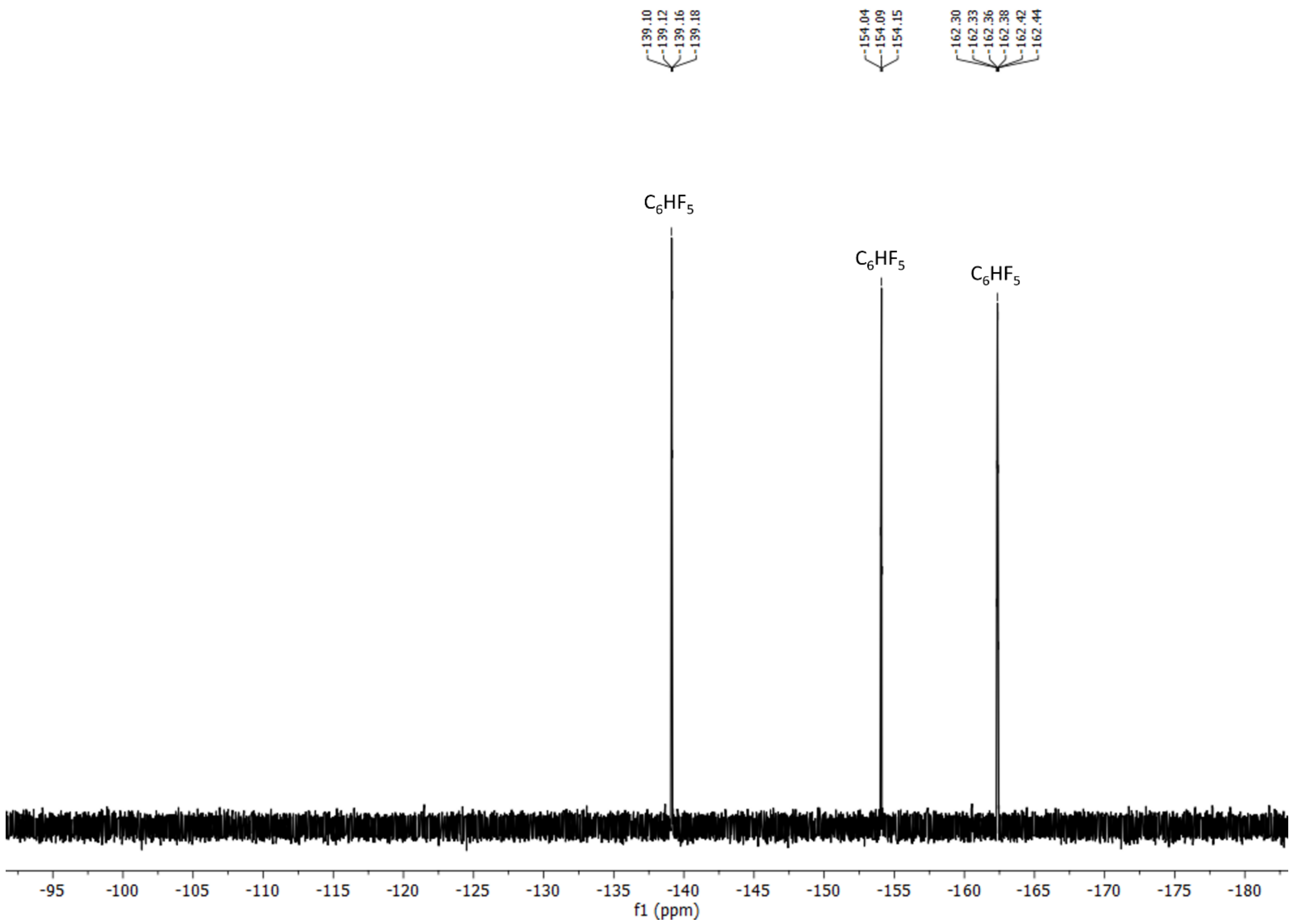

Figure S37. $^{19}F\{^{1}H\}$ NMR spectrum of the crude reaction mixture following the addition of 20 mol% of HO_2CR^{non} to **1-OZn(C_6F_5)** (benzene-d$_6$/toluene, 377 MHz, 298 K). ^{19}F chemical shifts for C_6HF_5 (188 MHz, benzene-d$_6$): –139.4, –154.0 and –162.7 ppm.[7]

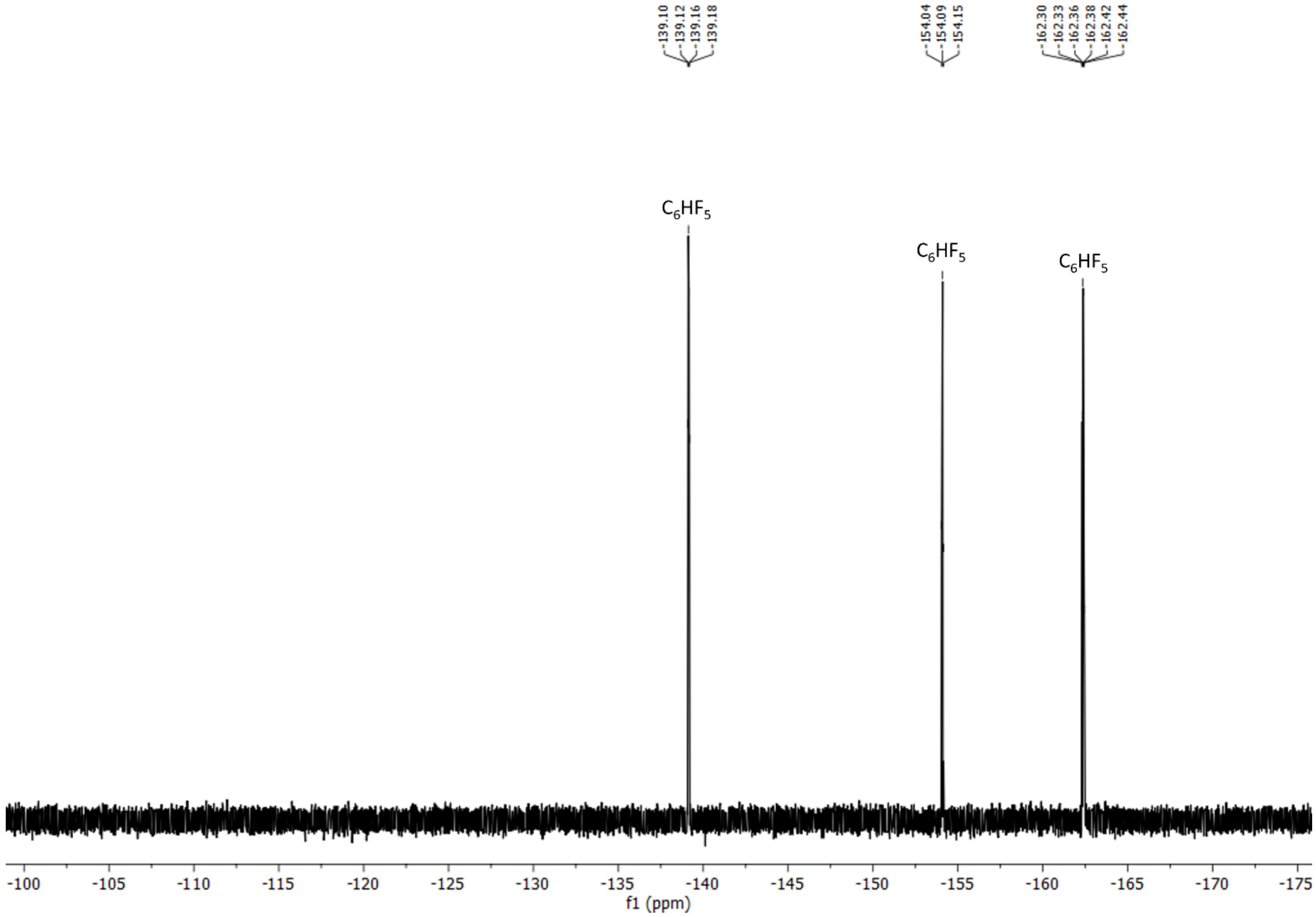

Figure S38. $^{19}F\{^1H\}$ NMR spectrum of the crude reaction mixture following the addition of 20 mol% of HO_2CR^{BrDA} to **1-OZn(C_6F_5)** (benzene-d_6/toluene, 377 MHz, 298 K). ^{19}F chemical shifts for C_6HF_5 (188 MHz, benzene-d_6): –139.4, –154.0 and –162.7 ppm.[7]

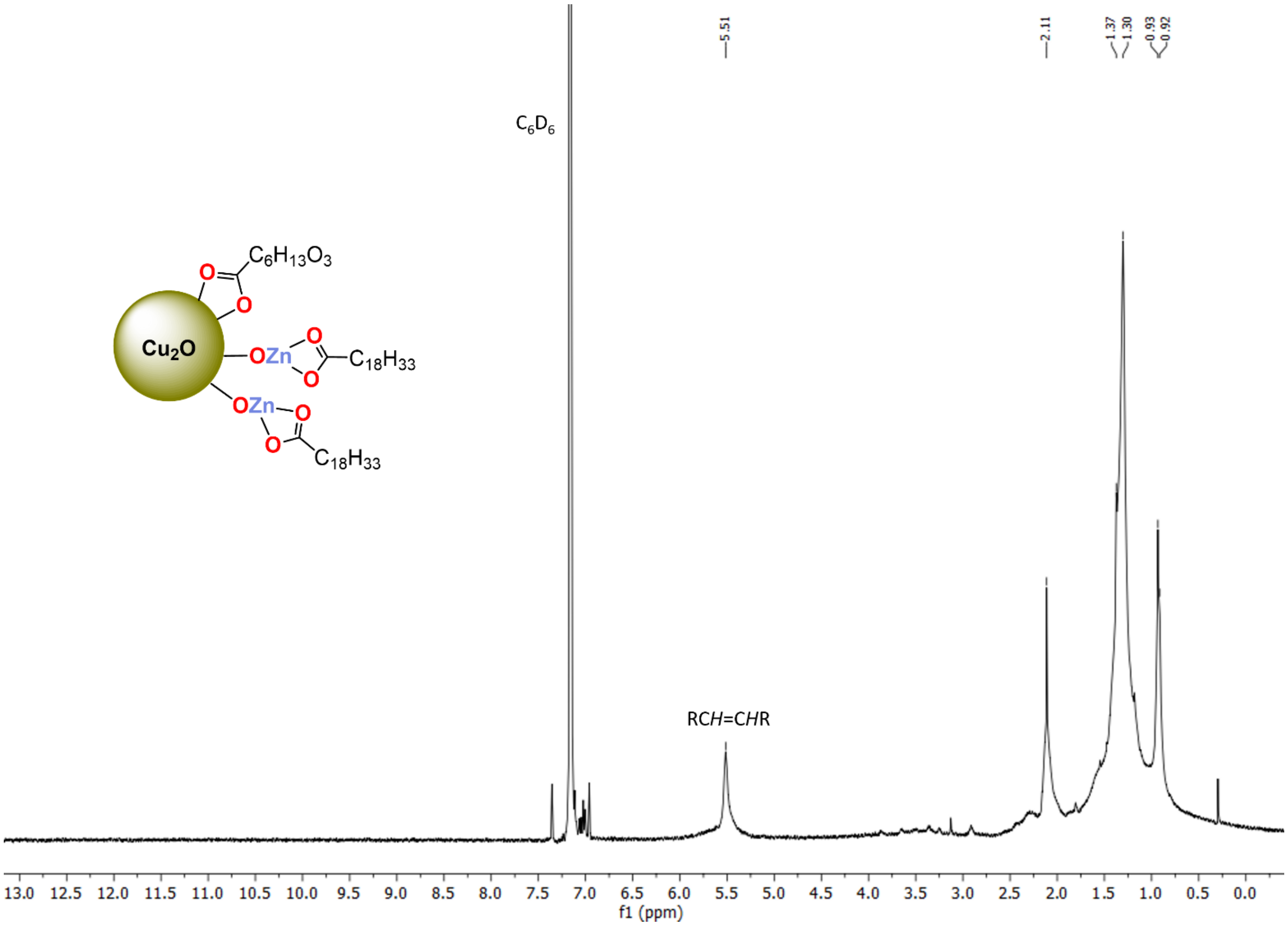

Figure S39. ^{1}H NMR spectrum of **1-OZn(O$_2$CR^{ole})** (benzene-d$_6$, 400 MHz, 298 K).

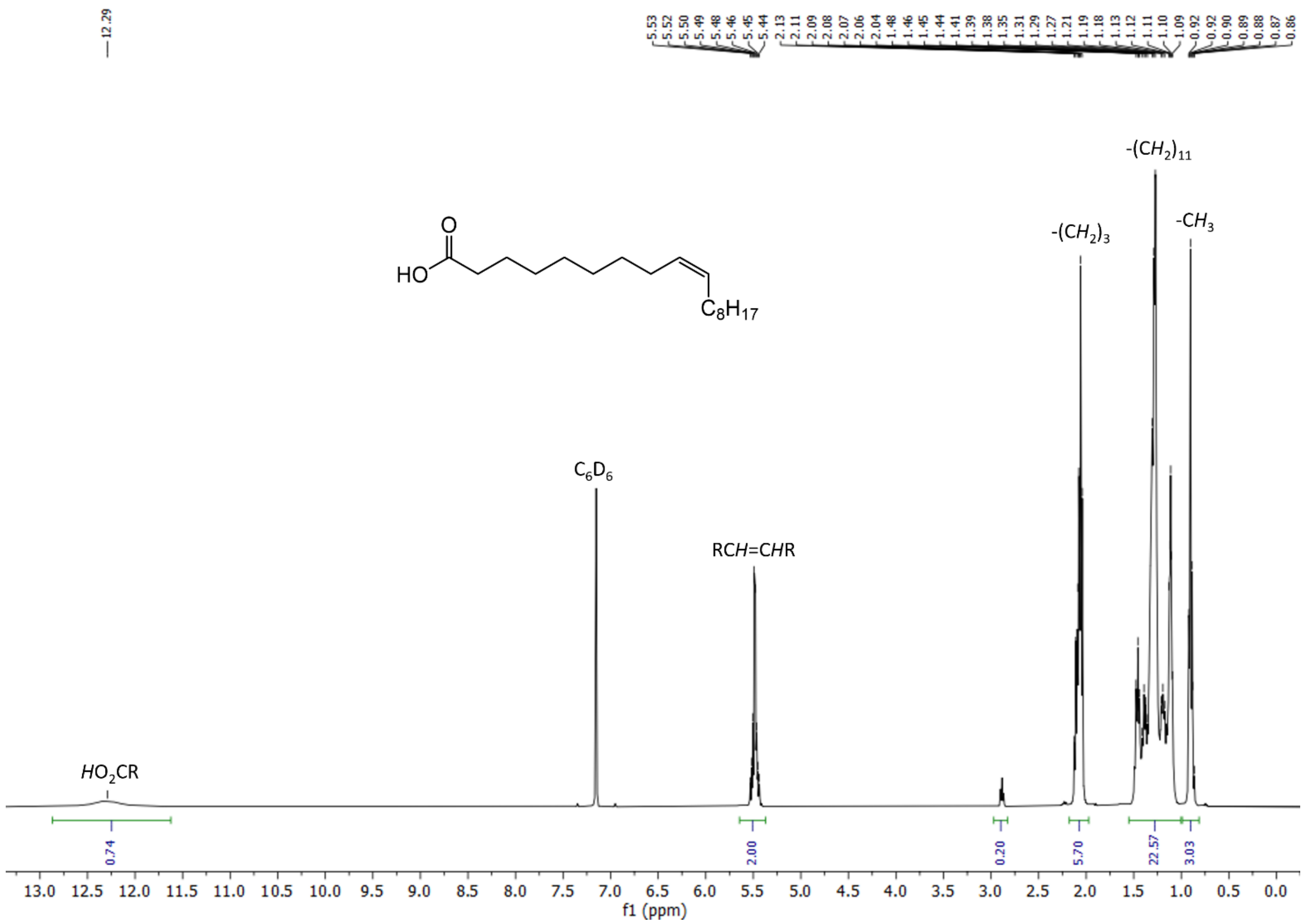

Figure S40. ^{1}H NMR spectrum of oleic acid (HO_2CR^{ole}; benzene-d_6, 400 MHz, 298 K).

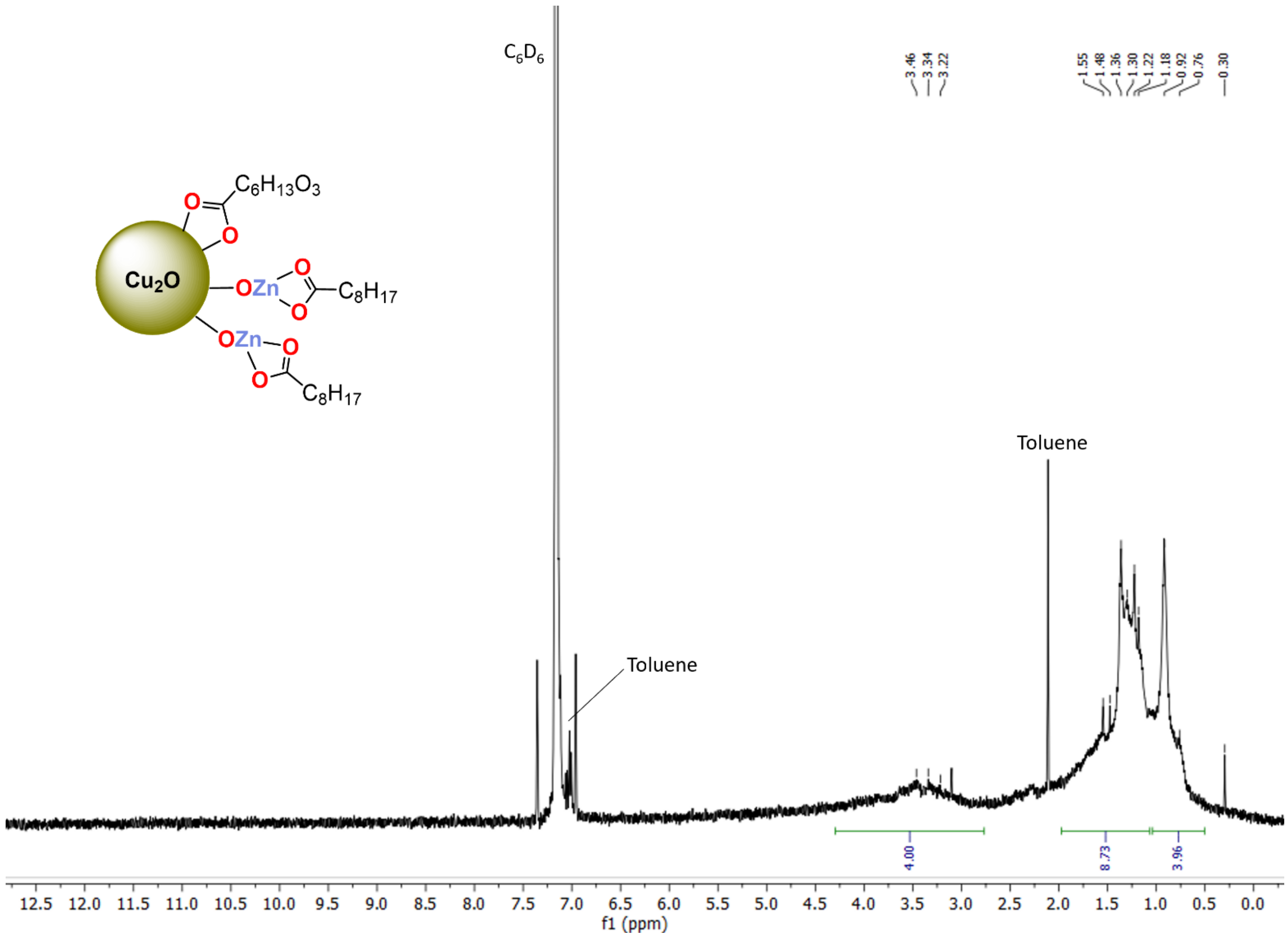

Figure S41. ^{1}H NMR spectrum of **1-OZn(O_2CR^{non})** (benzene-d_6, 400 MHz, 298 K).

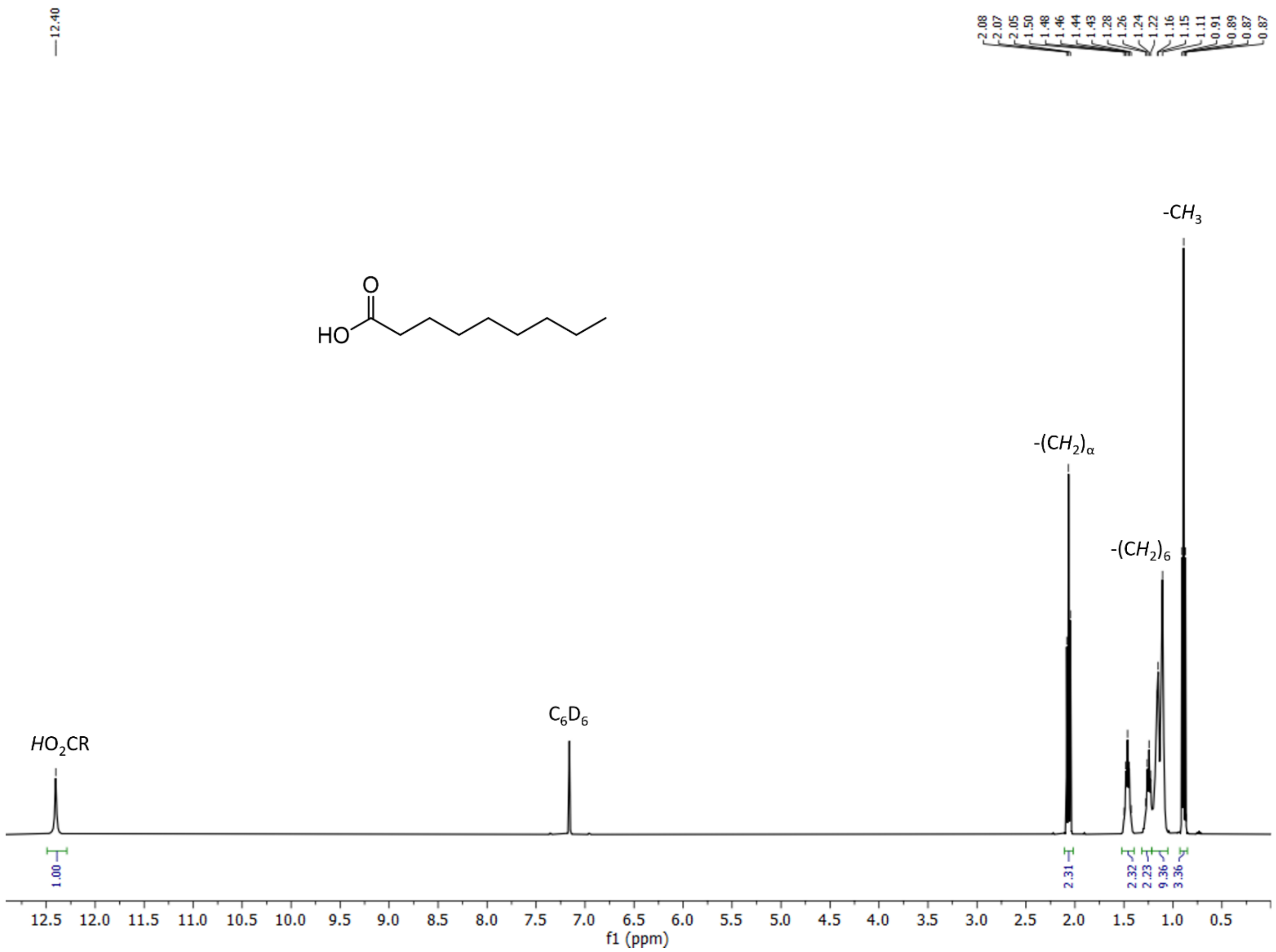

Figure S42. ^{1}H NMR spectrum of nonanoic acid (HO_2CR^{non}; benzene-d_6, 400 MHz, 298 K).

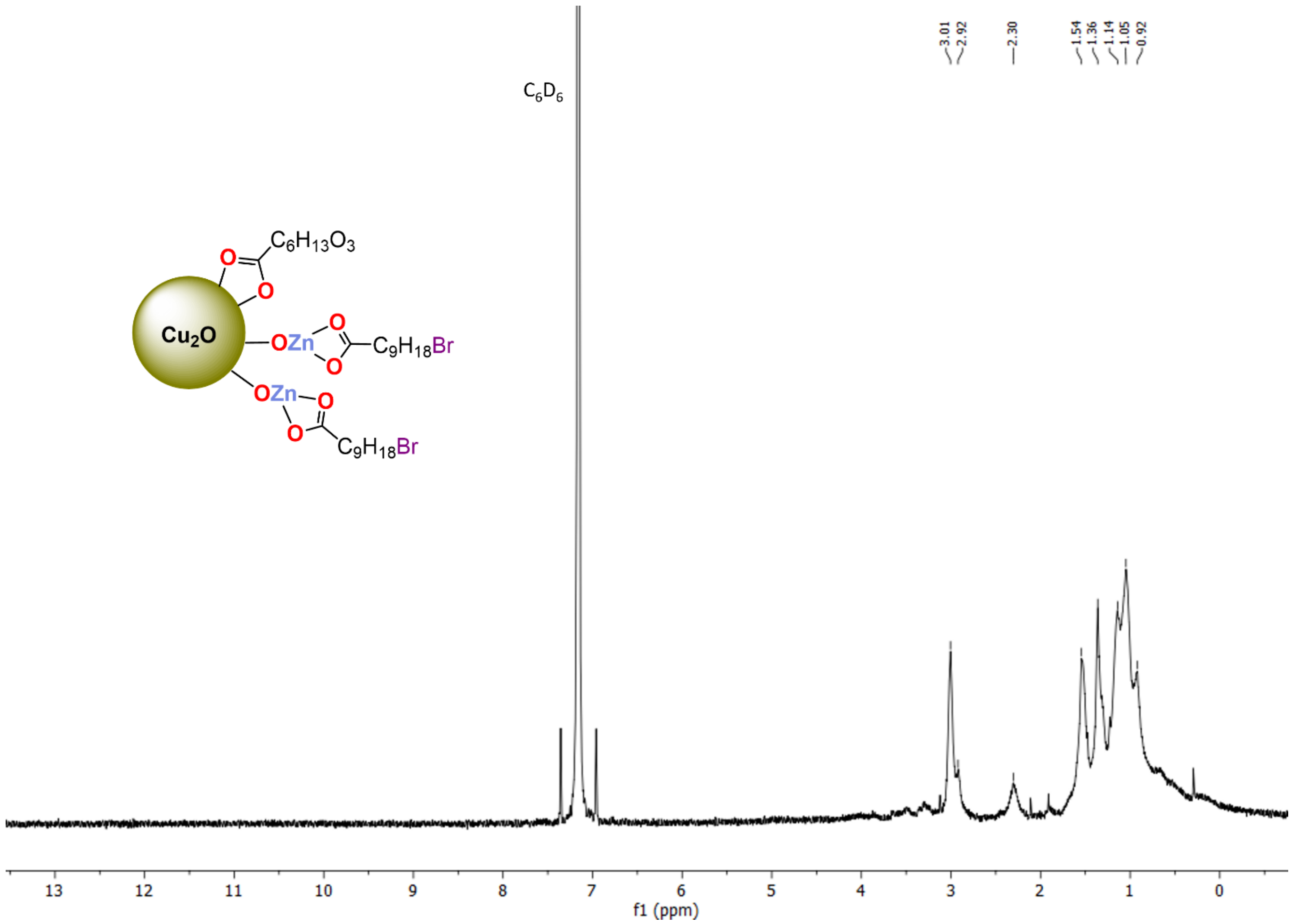

Figure S43. ^{1}H NMR spectrum of **1-OZn(O$_2$CR^{BrDA})** (benzene-d$_6$, 400 MHz, 298 K).

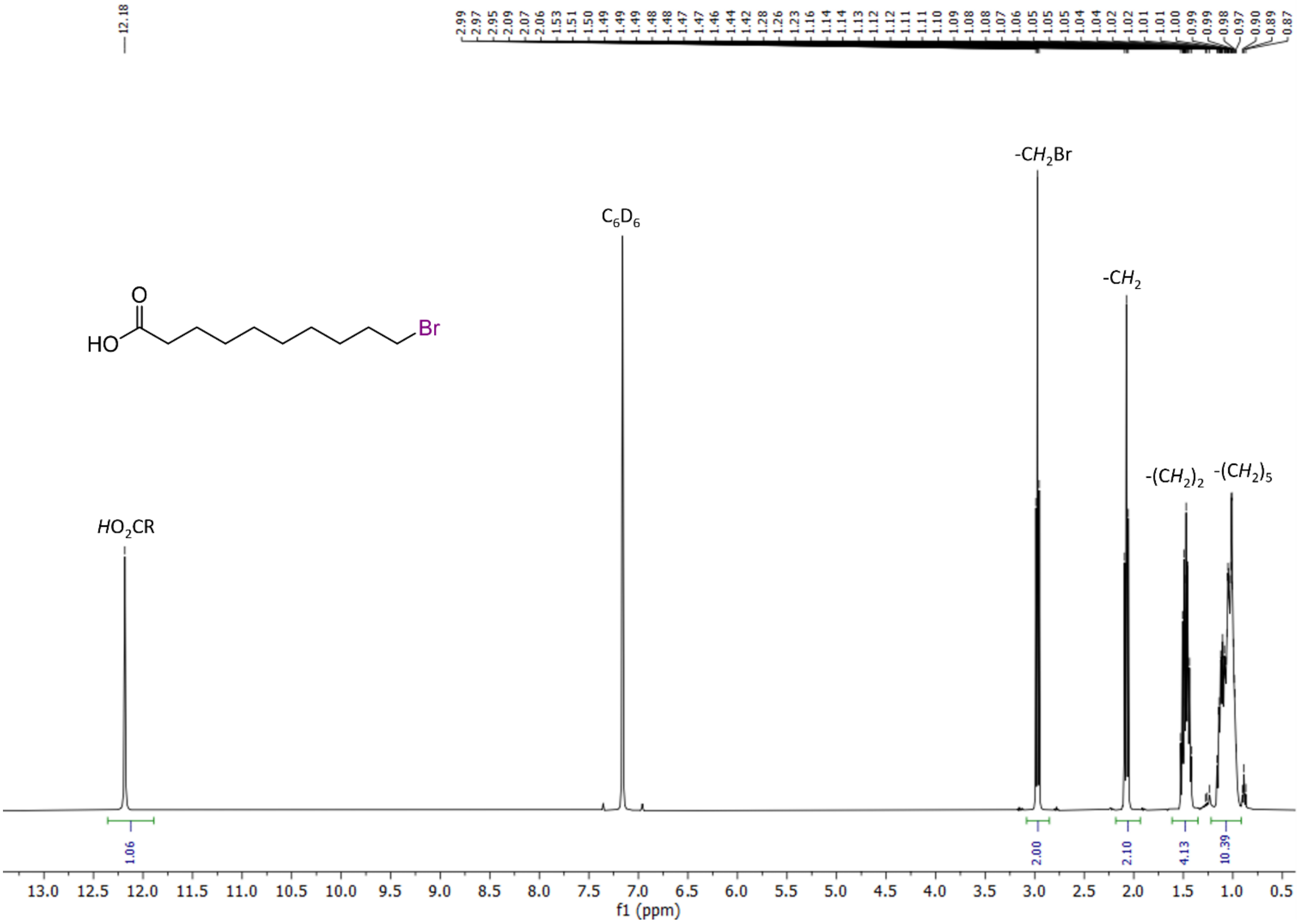

Figure S44. ^{1}H NMR spectrum of 10-bromodecanoic acid (HO_2CR^{BrDA}; benzene-d_6, 400 MHz, 298 K).

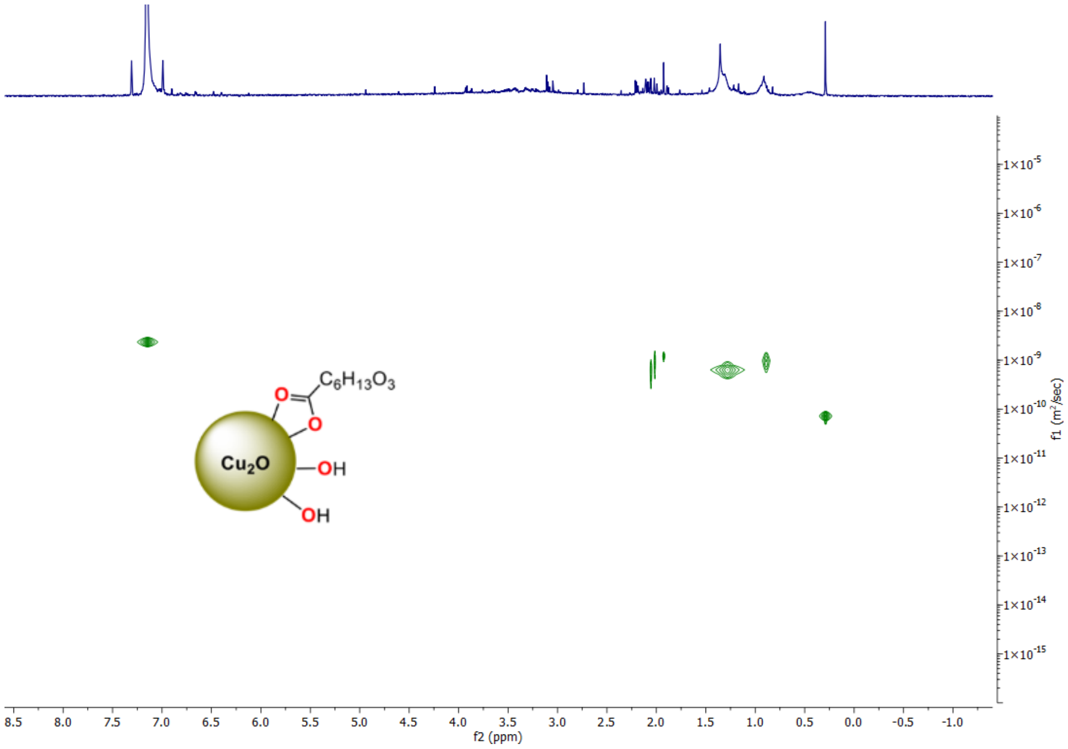

Figure S45. ^{1}H DOSY NMR spectrum of **1-OH** (benzene-d_6, 400 MHz, 298 K); diffusion coefficient = $5.22 \times 10^{-10} m^2 s^{s-1}$.

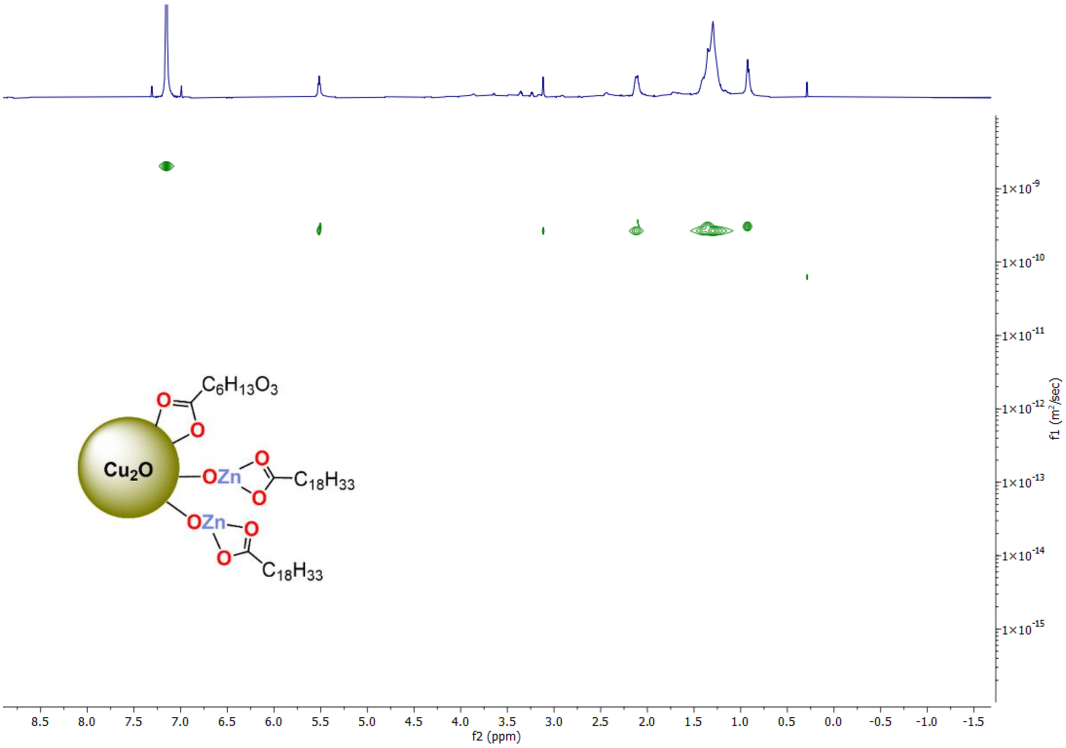

Figure S46. ^{1}H DOSY NMR spectrum of [**1-OZn(O$_2$CR^{ole})**] (benzene-d$_6$, 400 MHz, 298 K); diffusion coefficient = 2.53×10^{-10} m^{2}s^{-1}.

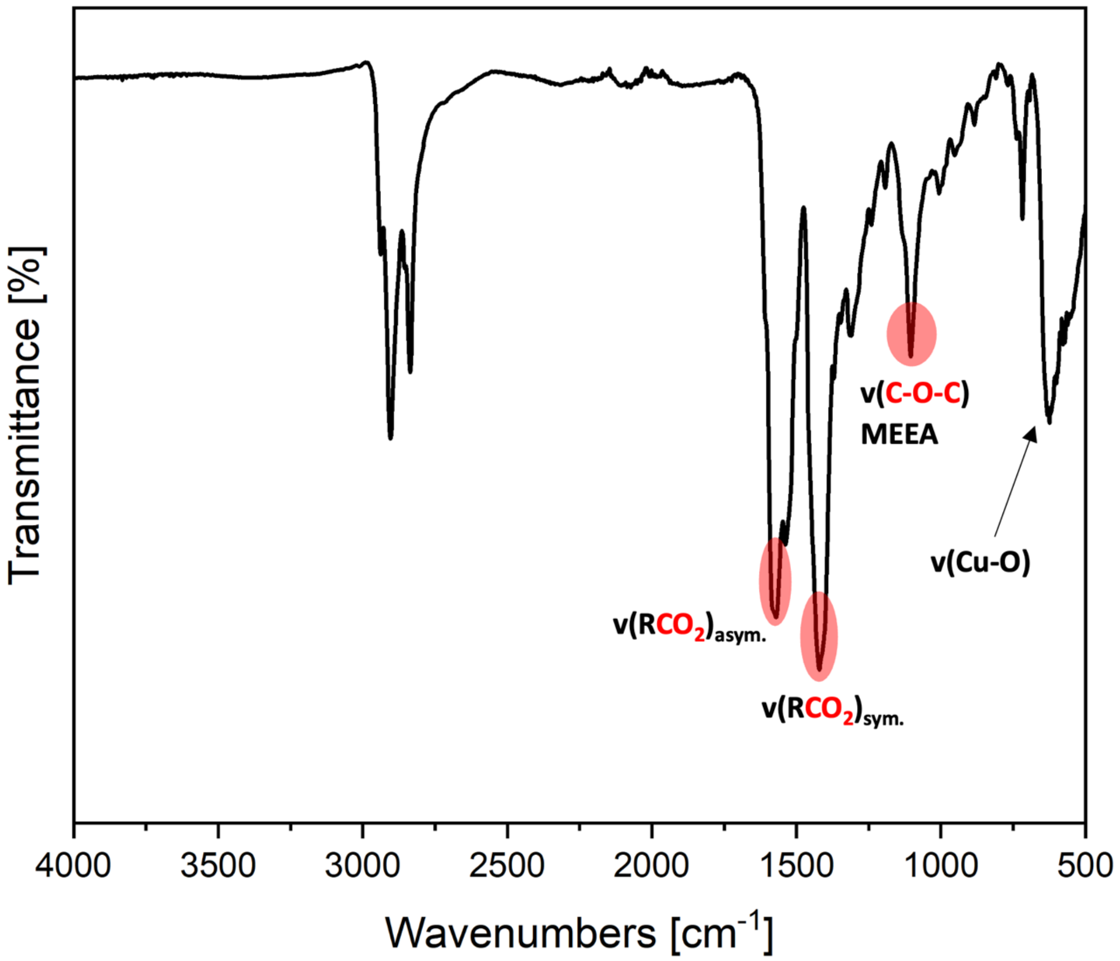

Figure S47. FT-IR spectrum of **1-OZn(O$_2$CR^{non})**. Stretches associated with -C_6F_5 are not observed (see Figures S16 and S17 for comparison).

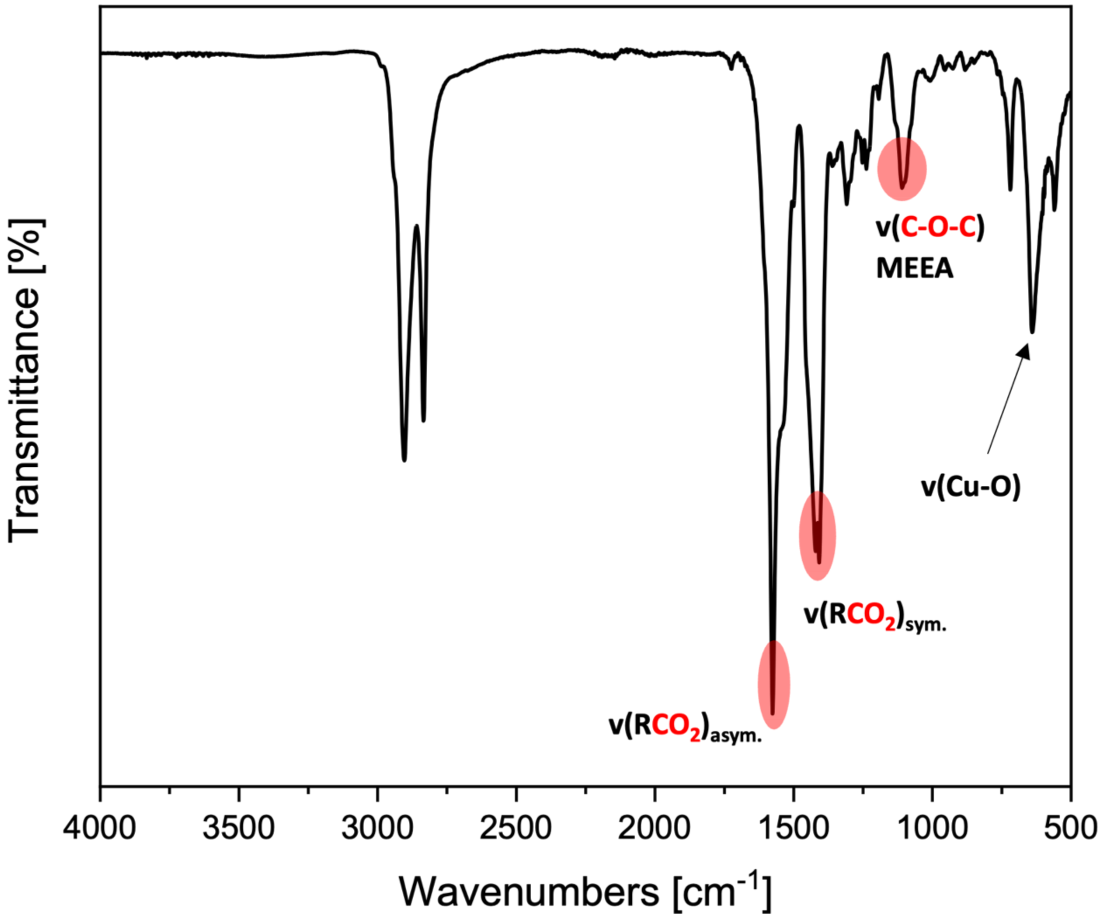

Figure S48. FT-IR spectrum of **1-OZn(O$_2$CR^{BrDA})**. Stretches associated with -C_6F_5 are not observed (see Figures S16 and S17 for comparison).

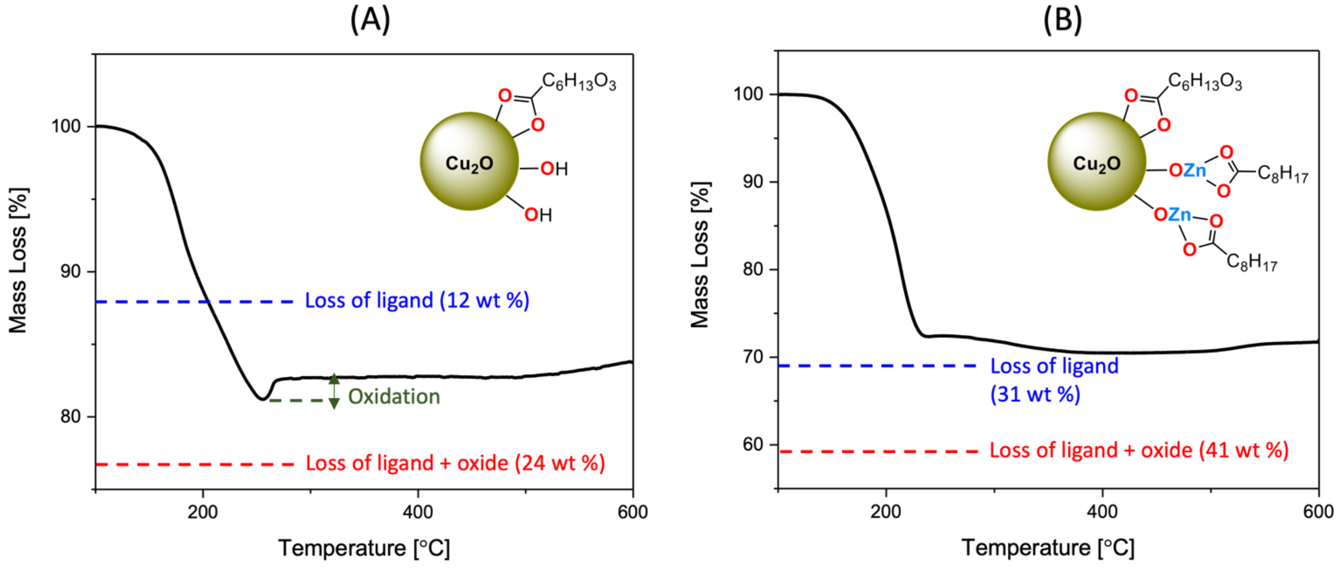

Figure S49. Comparison between TGA thermograms of **(A) 1-OH**, and **(B) 1-OZn(O$_2$CR^{non})**. In both thermograms, the theoretical mass loss from loss of ligand is indicated by a blue dashed line, and the theoretical mass loss from loss of both ligand and oxide by a red dashed line. A mass increase is observed above 250 °C in **(A)**, which represents oxidation of Cu formed during the TGA experiment and is indicated in green.

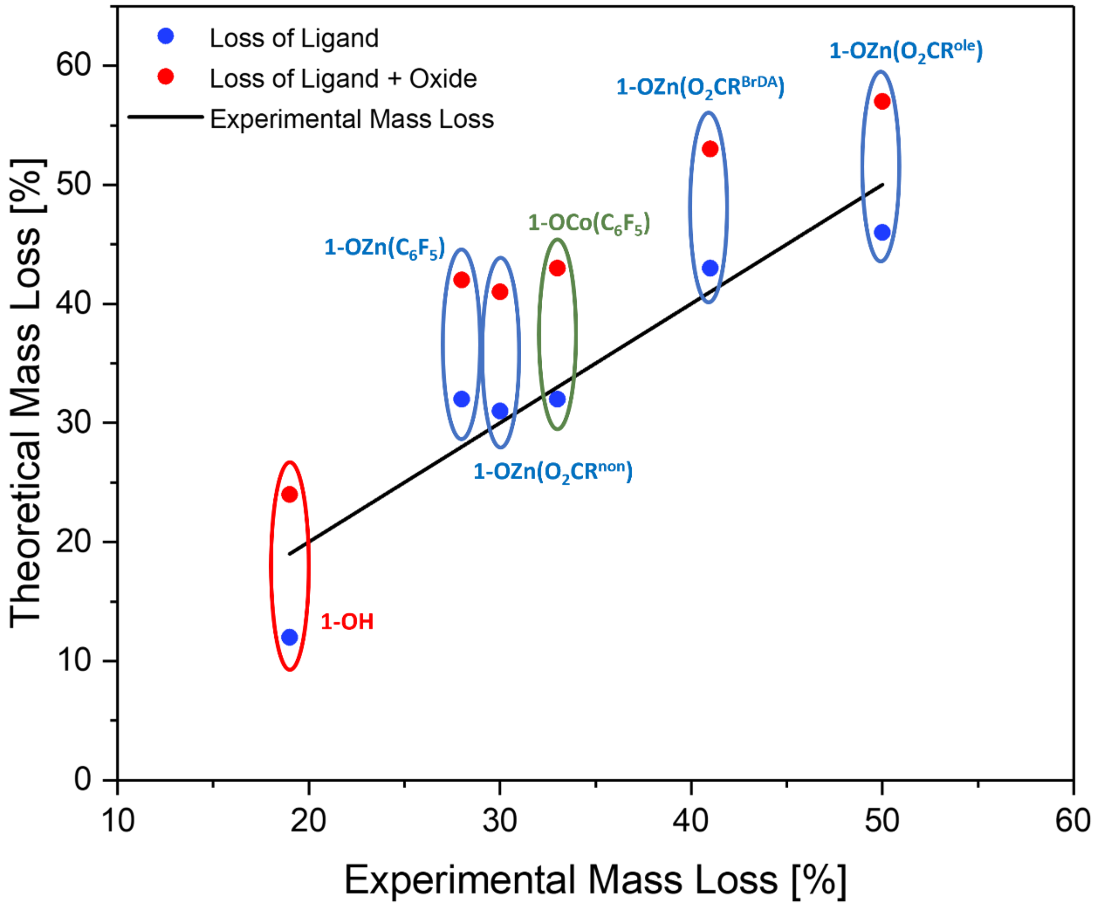

Figure S50. Experimental mass loss *versus* theoretical mass loss for thermal gravimetric analyses of **1-OH**, **1-OM(C_6F_5)** (M = Zn, Co) and **1-OZn(O_2CR')** (R' = R^{ole}, R^{non}, R^{BrDA}); blue data points represent loss of ligand, red data points represent loss of ligand + oxide, and the line of best fit represents the mass loss observed experimentally, and indicates that while loss of ligand + oxide is observed for **1-OH**, only loss of ligand is observed for **1-OM(C_6F_5)** and **1-OZn(O_2CR')**.

Table S4. Experimental and theoretical mass loss for **1-OH**, **1-OM(C_6F_5)** (M = Zn, Co) and **1-OZn(O_2CR')** (R' = R^{ole}, R^{non}, R^{BrDA}); the theoretical mass losses in bold and green font most closely match the experimental values.

Material	Experimental Mass Loss [%]	Theoretical Mass Loss for Loss of Ligand [%]	Theoretical Mass Loss for Loss of Ligand + Oxide [%]
**1-OH**	19	12	**24**
**1-OZn(C_6F_5)**	28	**32**	42
**1-OCo(C_6F_5)**	33	**32**	43
**1-OZn(O_2CR^{ole})**	50	**46**	57
**1-OZn(O_2CR^{non})**	30	**31**	41
**1-OZn(O_2CR^{BrDA})**	41	**43**	53

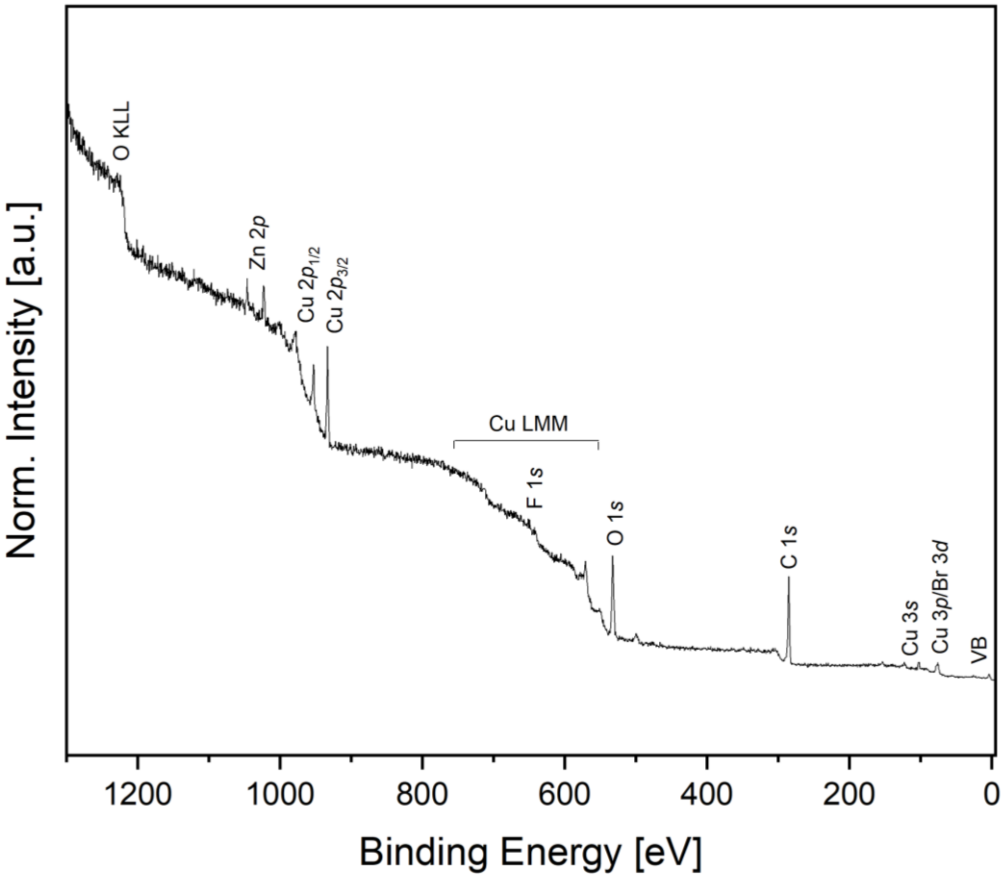

Figure S51. Survey XP spectrum of **1-OZn(O$_2$CR^{ole})**.

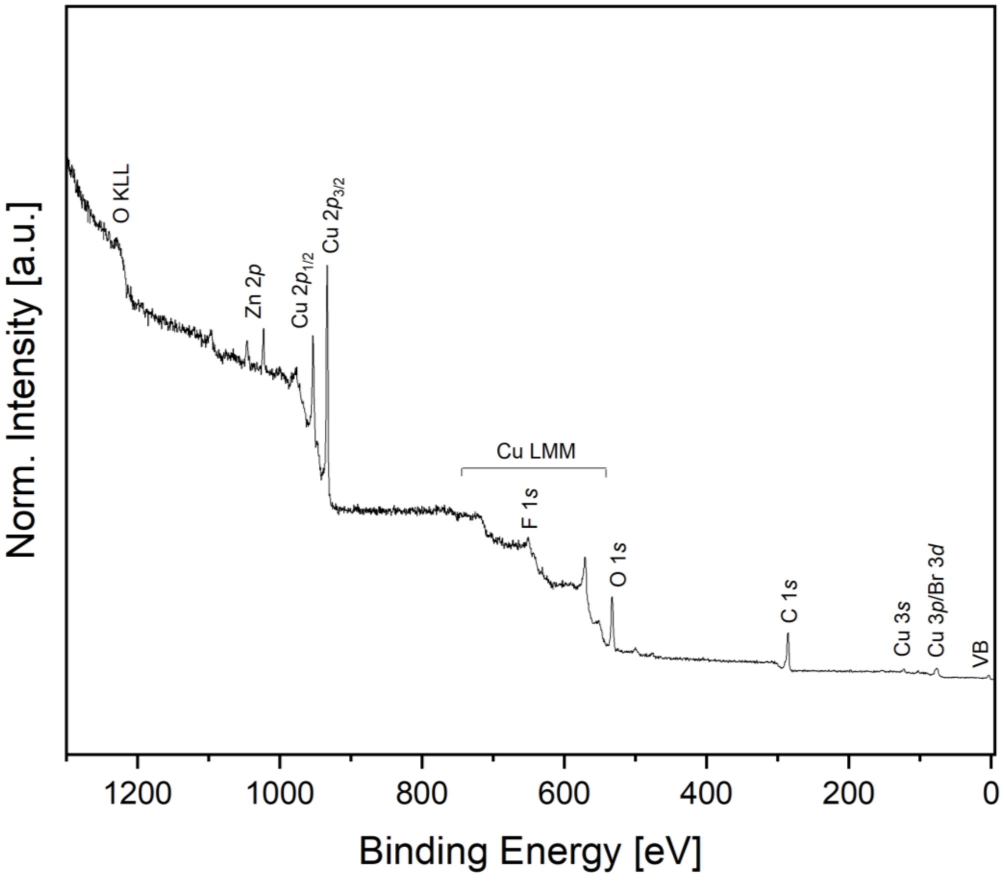

Figure S52. Survey XP spectrum of **1-OZn(O$_2$CR^{non})**.

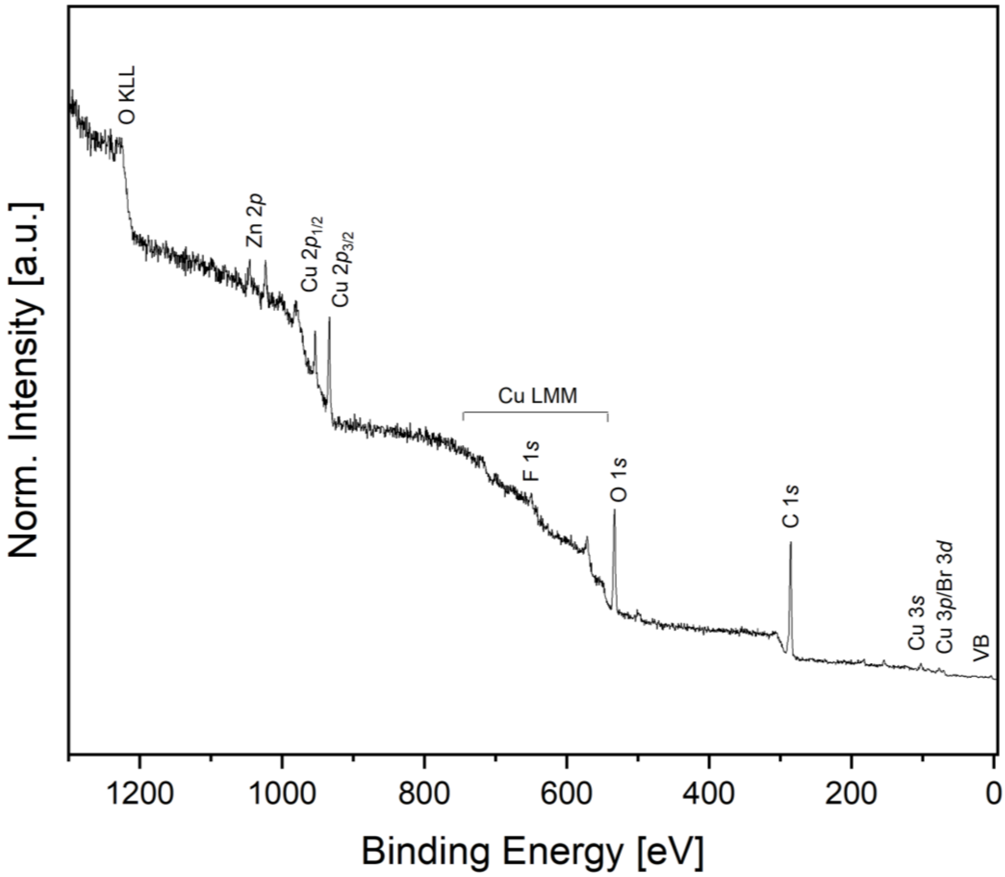

Figure S53. Survey XP spectrum of **1-OZn(O_2CR^{BrDA})**.

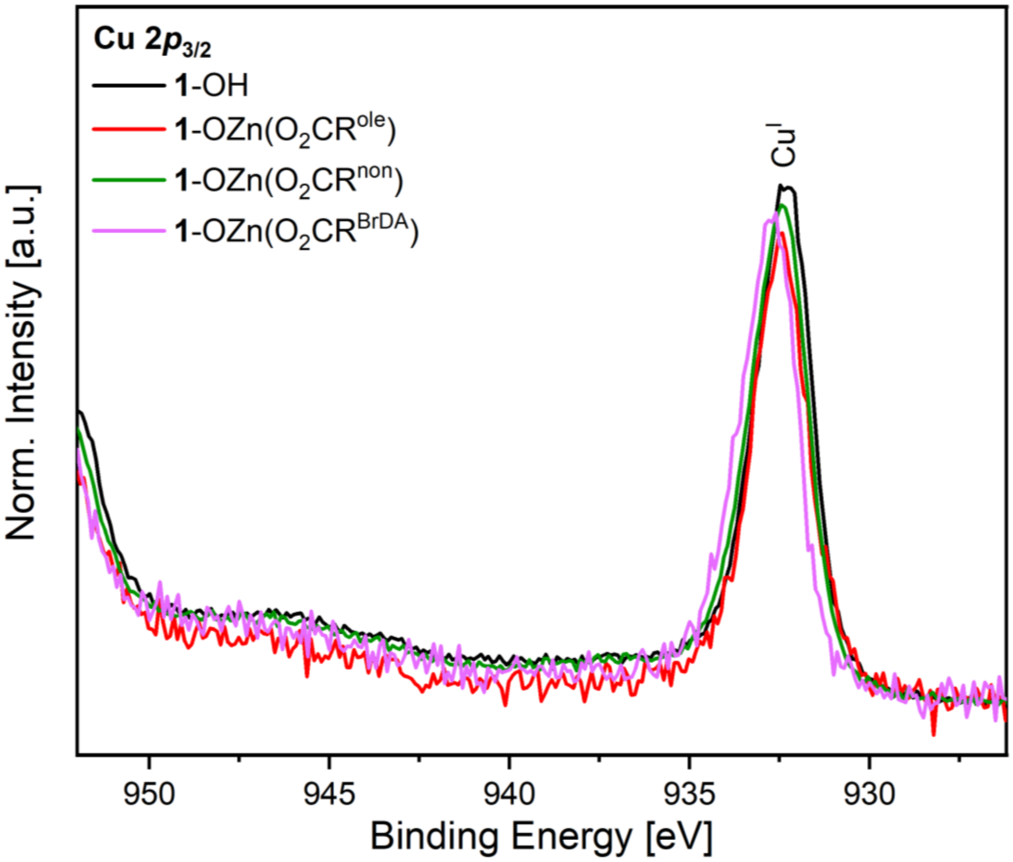

Figure S54. Cu 2$p_{3/2}$ XP spectrum of **1-OH**, **1-OZn(O_2CR^{ole})**, **1-OZn(O_2CR^{non})** and **1-OZn(O_2CR^{BrDA})**. A peak position of ~932.5 eV is diagnostic of Cu^{I}.[9-12]

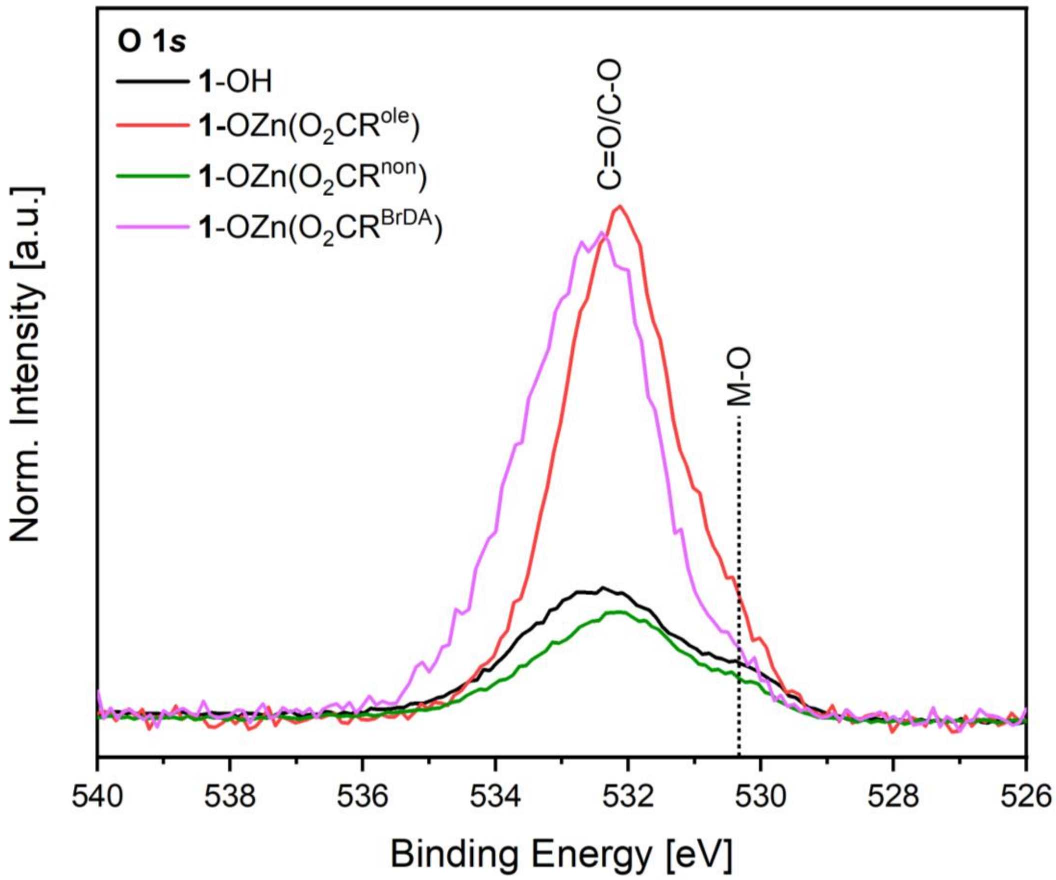

Figure S55. O 1*s* XP spectrum of **1-OH**, **1-OZn(O_2CR^{ole})**, **1-OZn(O_2CR^{non})** and **1-OZn(O_2CR^{BrDA})**. M-O environments of Cu_2O and C=O/C–O environments of the carboxylate ligands are observed.[14,15]

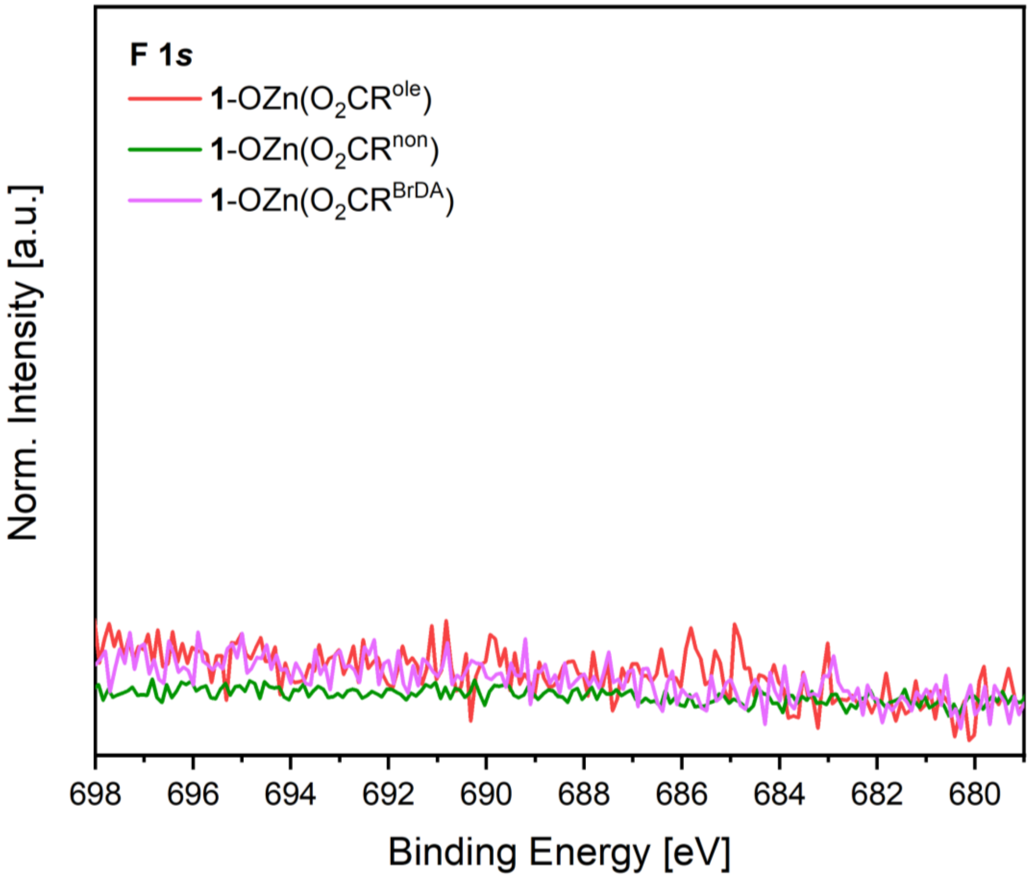

Figure S56. F 1*s* XP spectrum of **1-OZn(O_2CR^{ole})**, **1-OZn(O_2CR^{non})** and **1-OZn(O_2CR^{BrDA})**. No fluorine environments were observed.

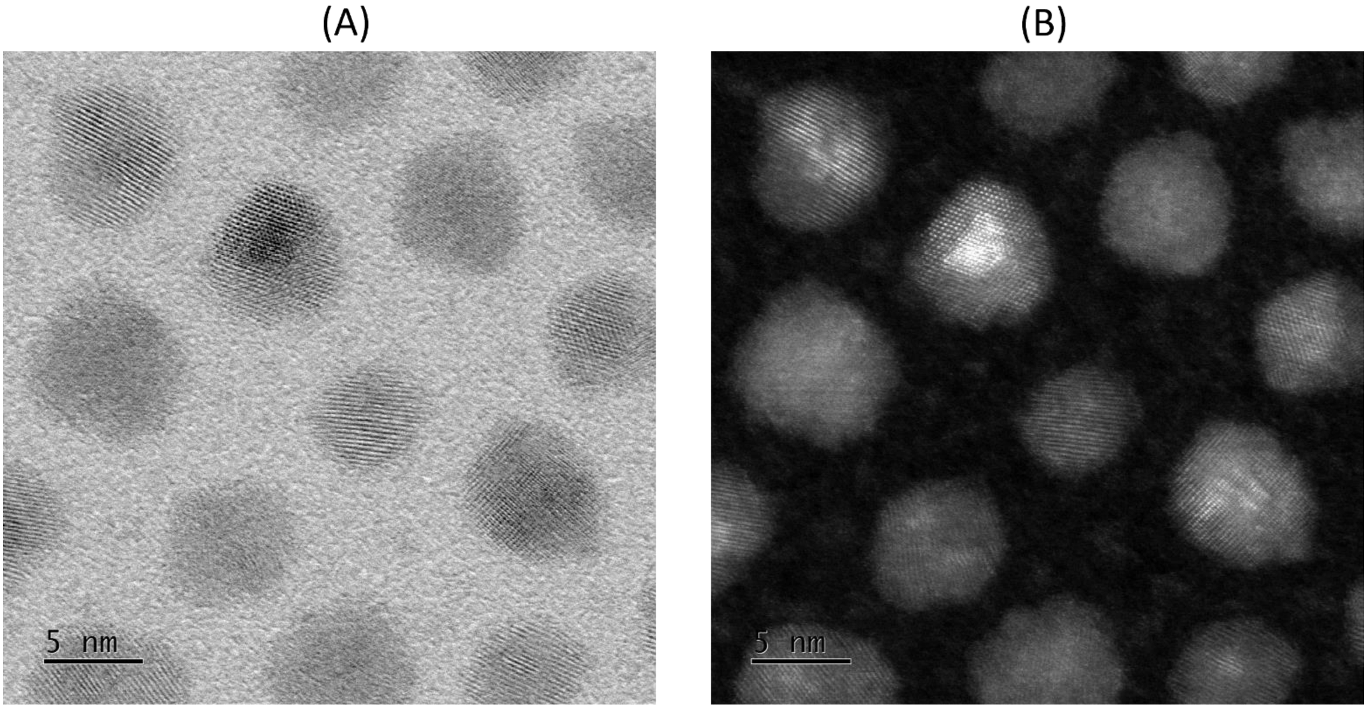

Figure S57. (A) Annular bright field STEM image and **(B)** high angle annular dark field STEM image of **1-OZn(O_2CR^{ole})**.

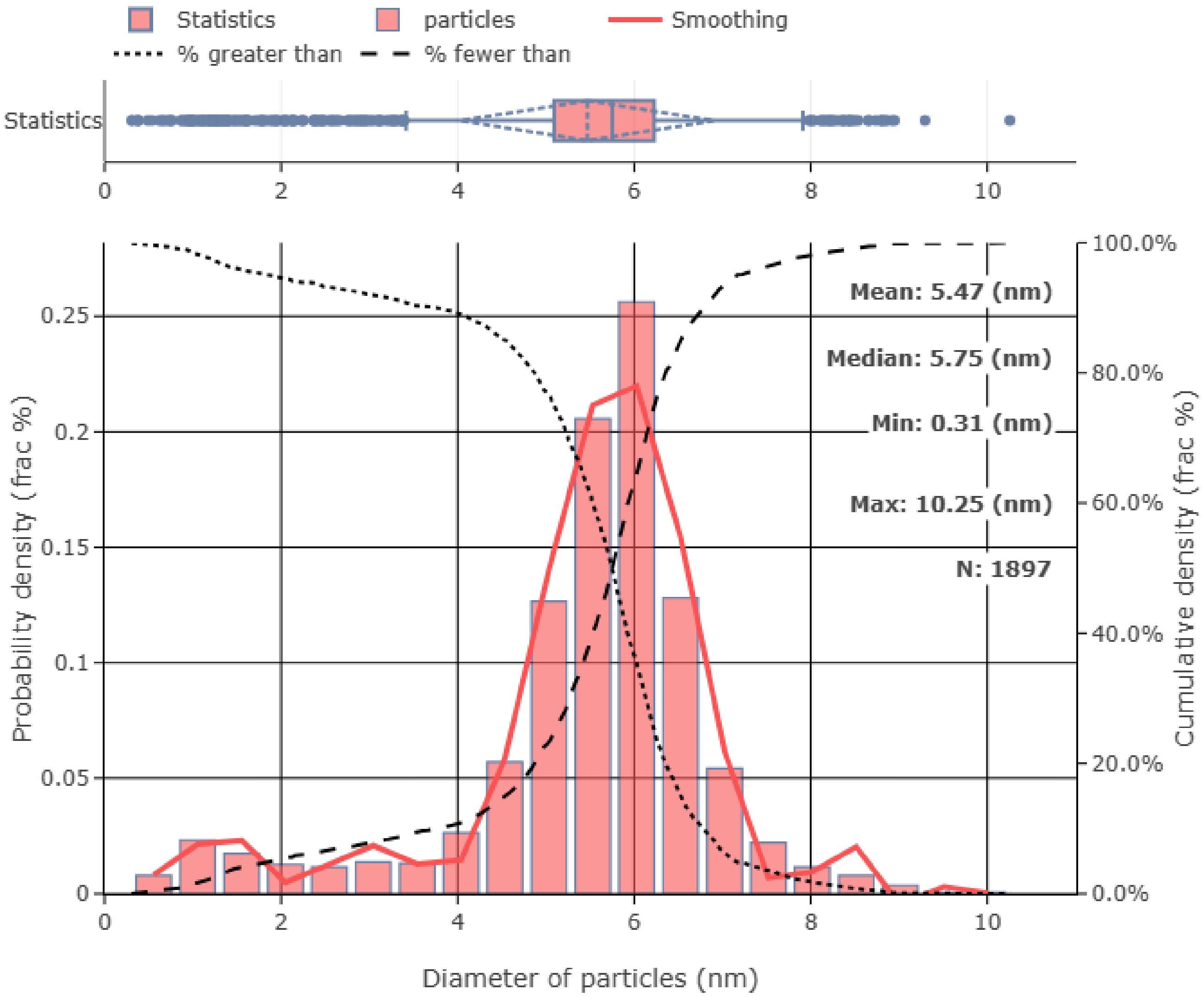

Figure S58. Size distribution histogram obtained from the TEM data for **1-OZn(O_2CR^{ole})**. Average particle size = 5.5 ± 1.4 nm.

(A)

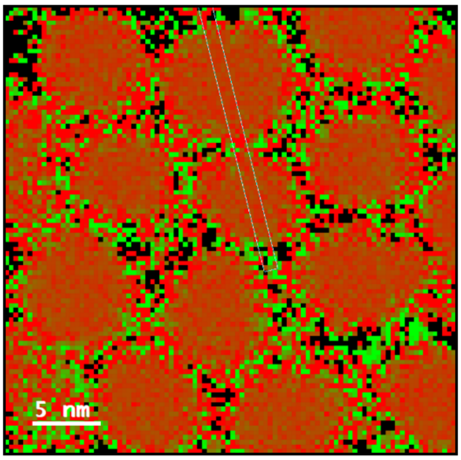

(B)

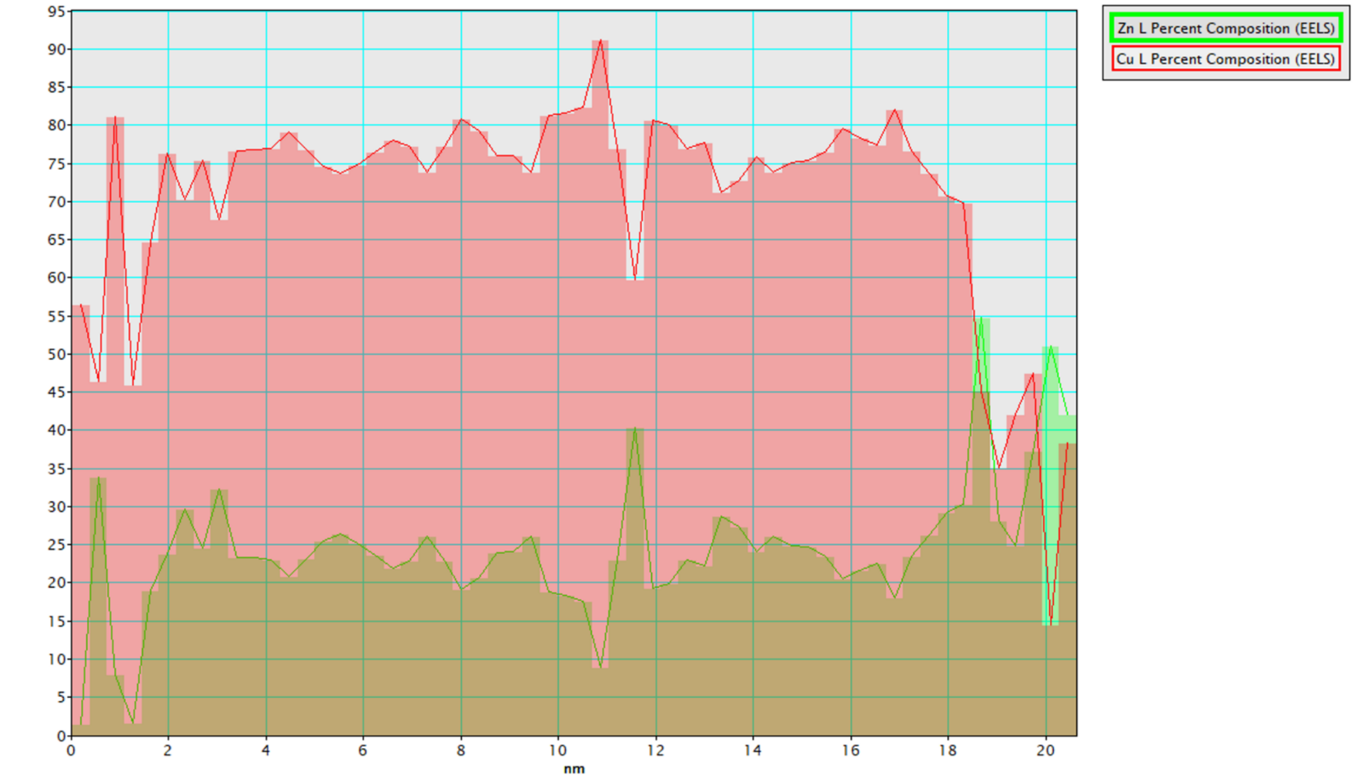

Figure S59. (A) Composite profile for **1-OZn(O_2CR^{ole})** (red = Cu, green = Zn) and **(B)** percent composition of Cu and Zn across two nanocrystals, indicated by a white box in **(A)**, determined using EELS.

(A)

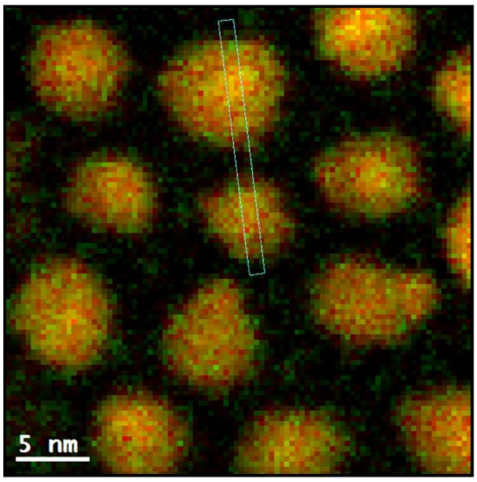

(B)

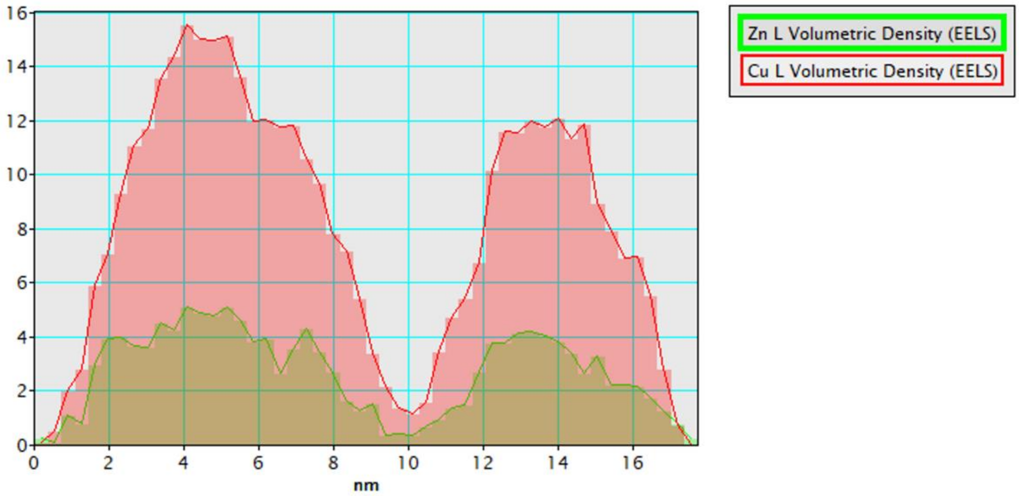

Figure S60. (A) Composite volume profile for **1-OZn(O$_2$CR^{ole})** (red = Cu, green = Zn) and **(B)** percent volumetric density of Cu and Zn across two nanocrystals, indicated by a white box in **(A)**, determined using EELS.

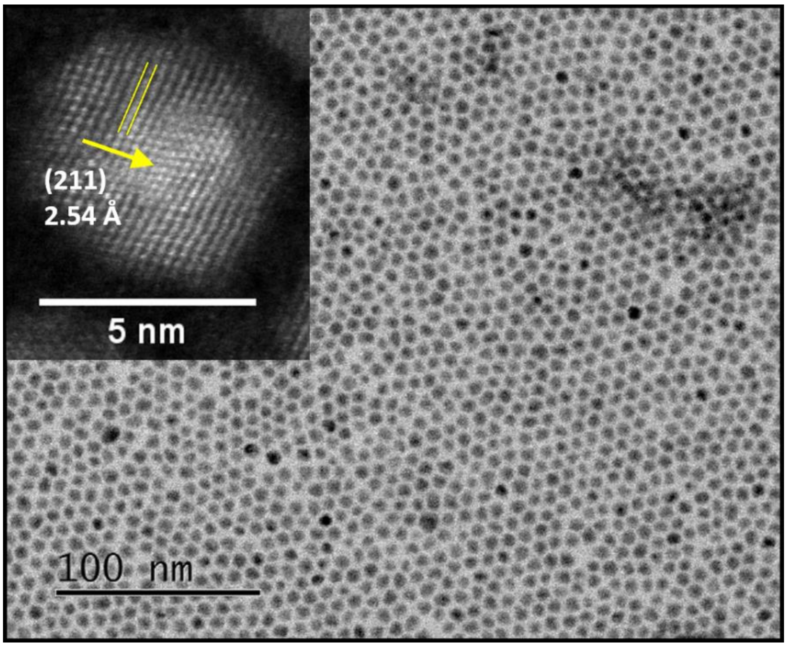

Figure S61. Annular bright field STEM image and HR-TEM image (top left)of **1-OZn(O_2CR^{non})**. The (211) lattice fringe, with a spacing of 2.54 Å, was located in the HR-TEM image.

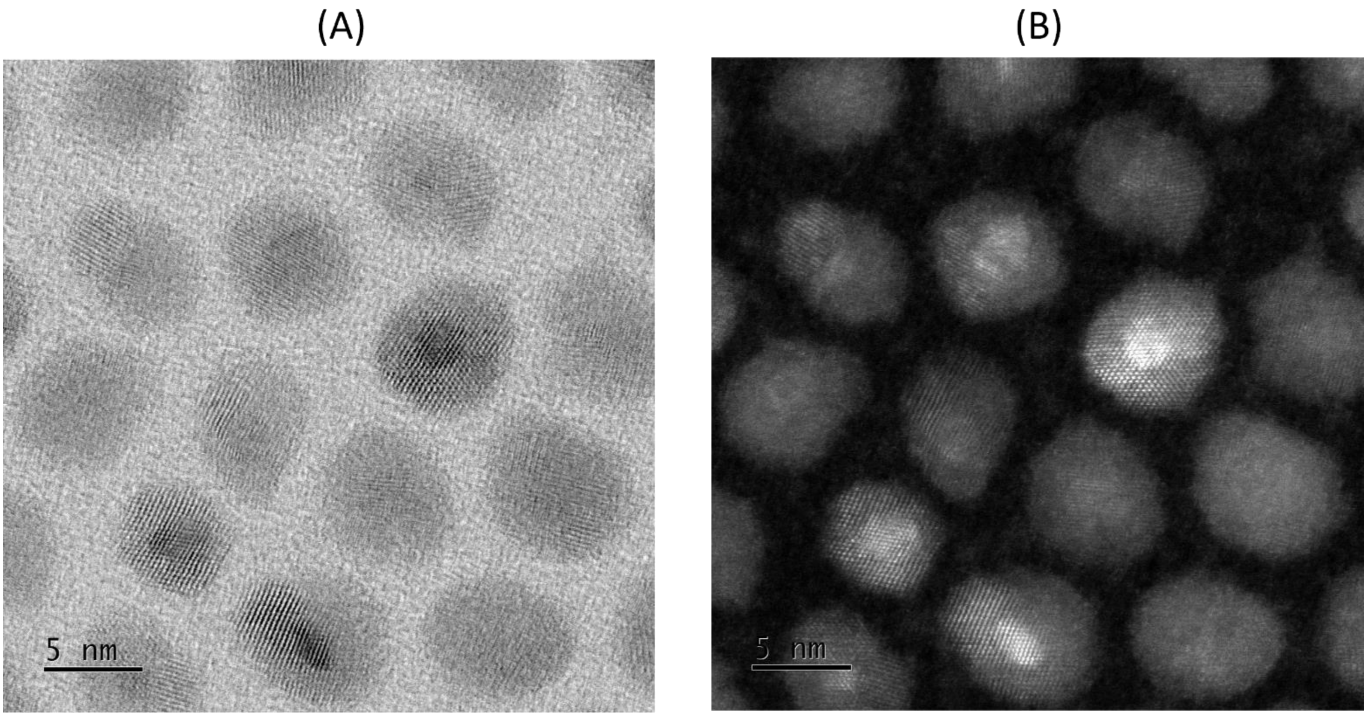

Figure S62. (A) Annular bright field STEM image and **(B)** high angle annular dark field STEM image of **1-OZn(O_2CR^{non})**.

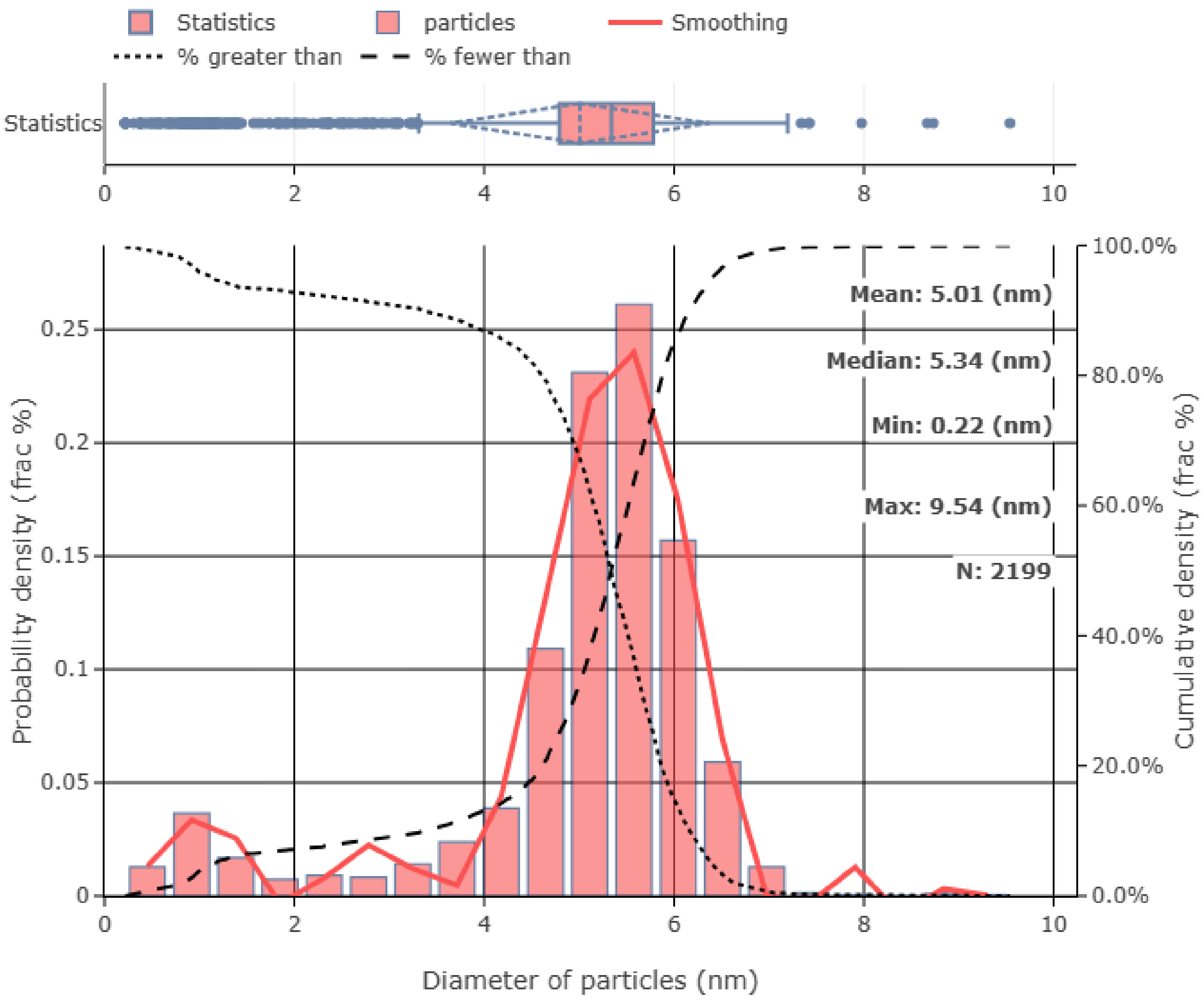

Figure S63. Size distribution histogram obtained from the TEM data for **1-OZn(O_2CR^{non})**. Average particle size = 5.0 ± 1.4 nm.

(A)

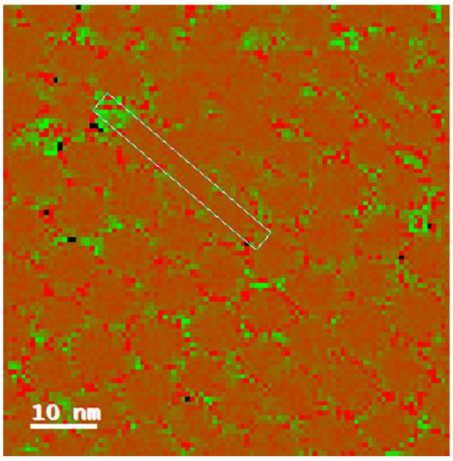

(B)

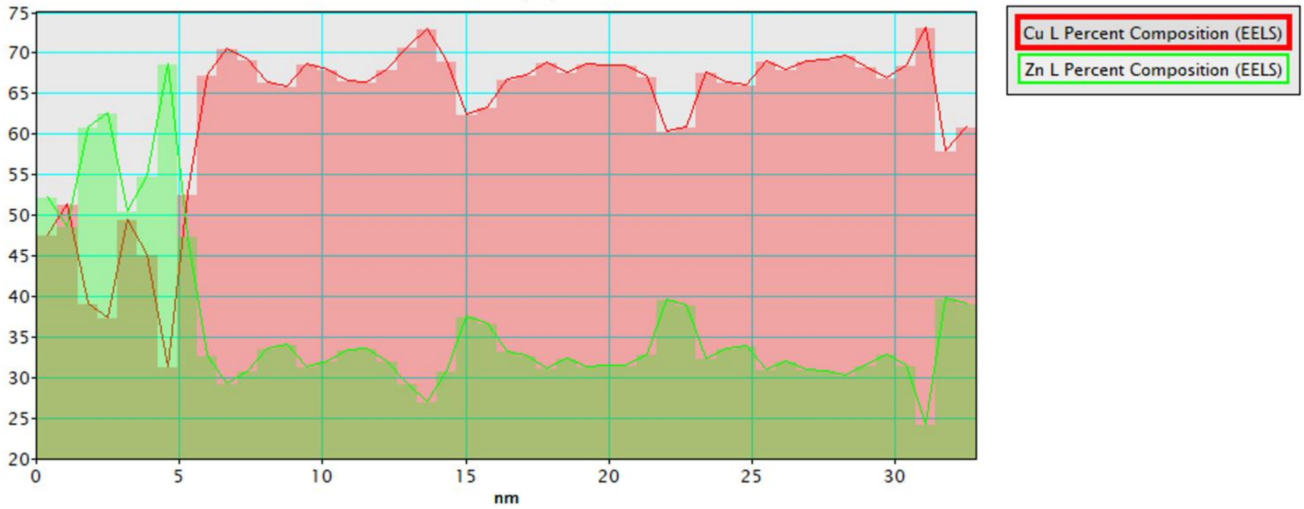

Figure S64. (A) Composite profile for **1-OZn(O$_2$CR^{non})** (red = Cu, green = Zn) and **(B)** percent composition of Cu and Zn across two nanocrystals, indicated by a white box in **(A)**, determined using EELS.

(A)

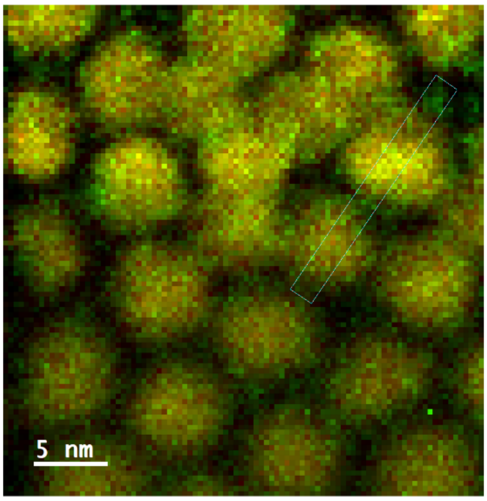

(B)

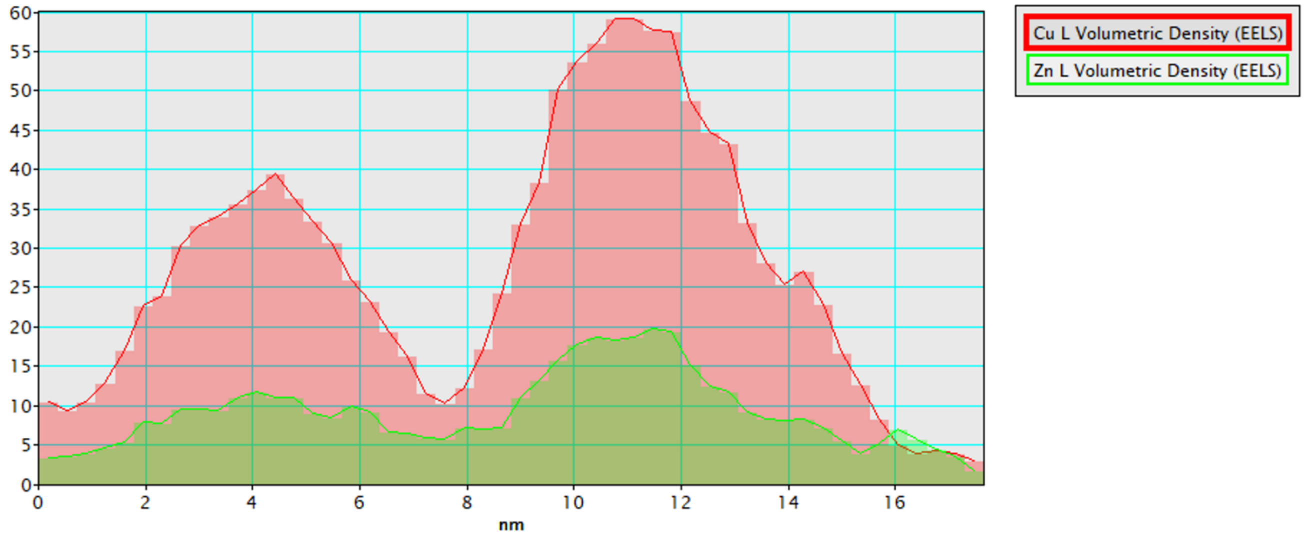

Figure S65. (A) Composite volume profile for **1-OZn(O$_2$CR^{non})** (red = Cu, green = Zn) and **(B)** percent volumetric density of Cu and Zn across two nanocrystals, indicated by a white box in **(A)**, determined using EELS.

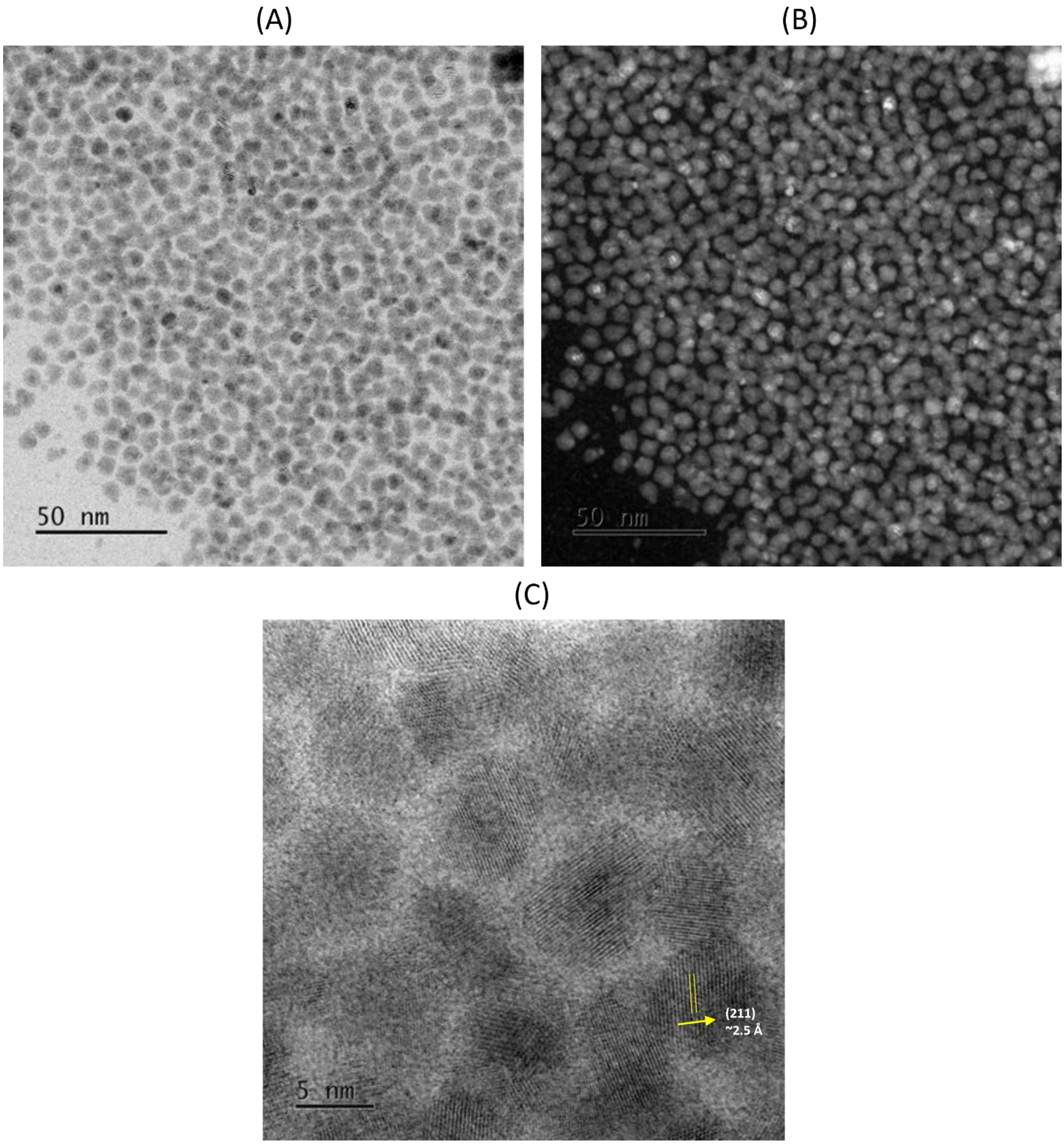

Figure S66. (A) Annular bright field STEM image, **(B)** high angle annular dark field STEM image and **(C)** annular bright field TEM image with (211) lattice fringe located for **1-OZn(O_2CR^{BrDA})**.

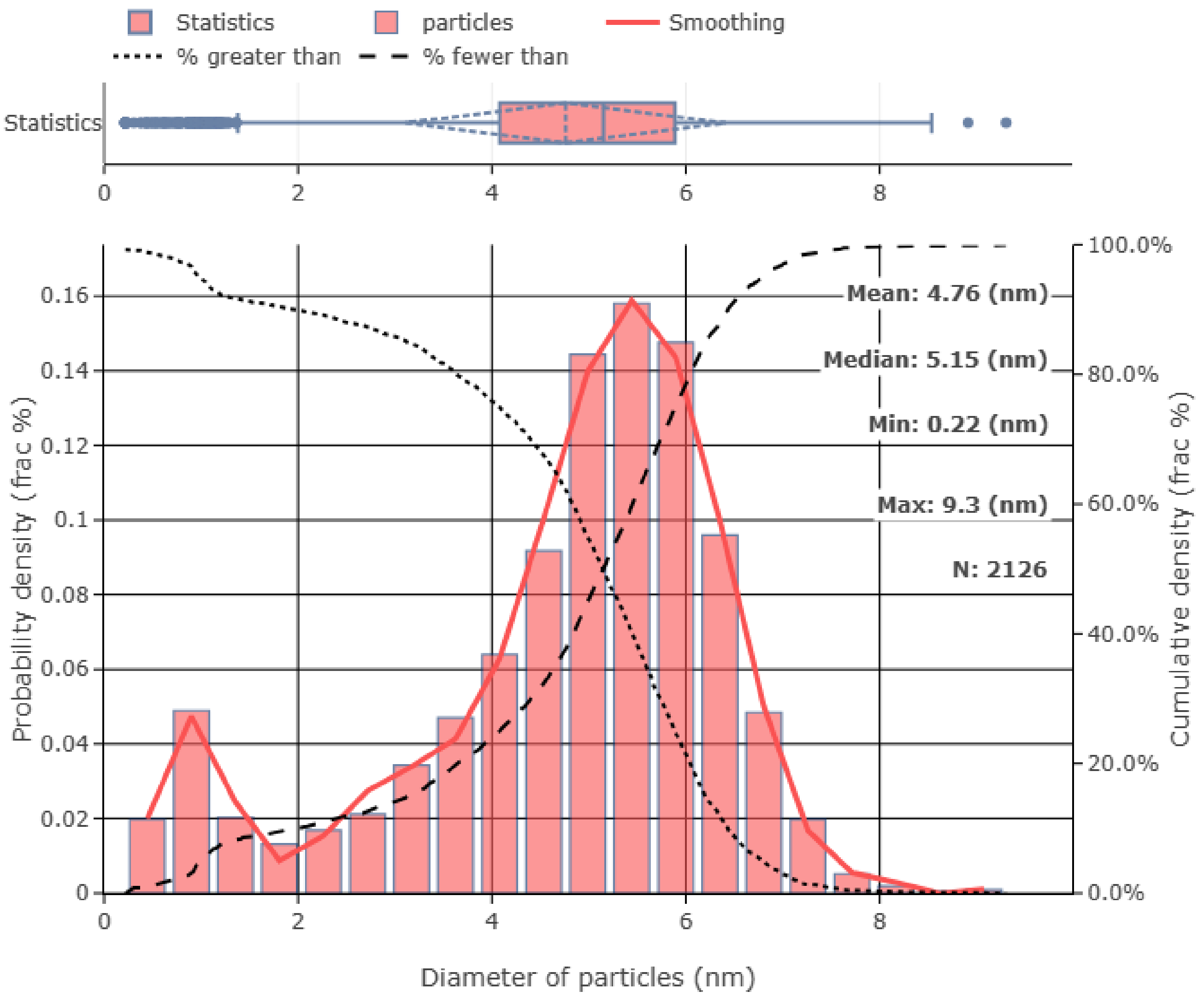

Figure S67. Size distribution histogram obtained from the TEM data for **1-OZn(O_2CR^{BrDA})**. Average particle size = 4.8 ± 1.7 nm.

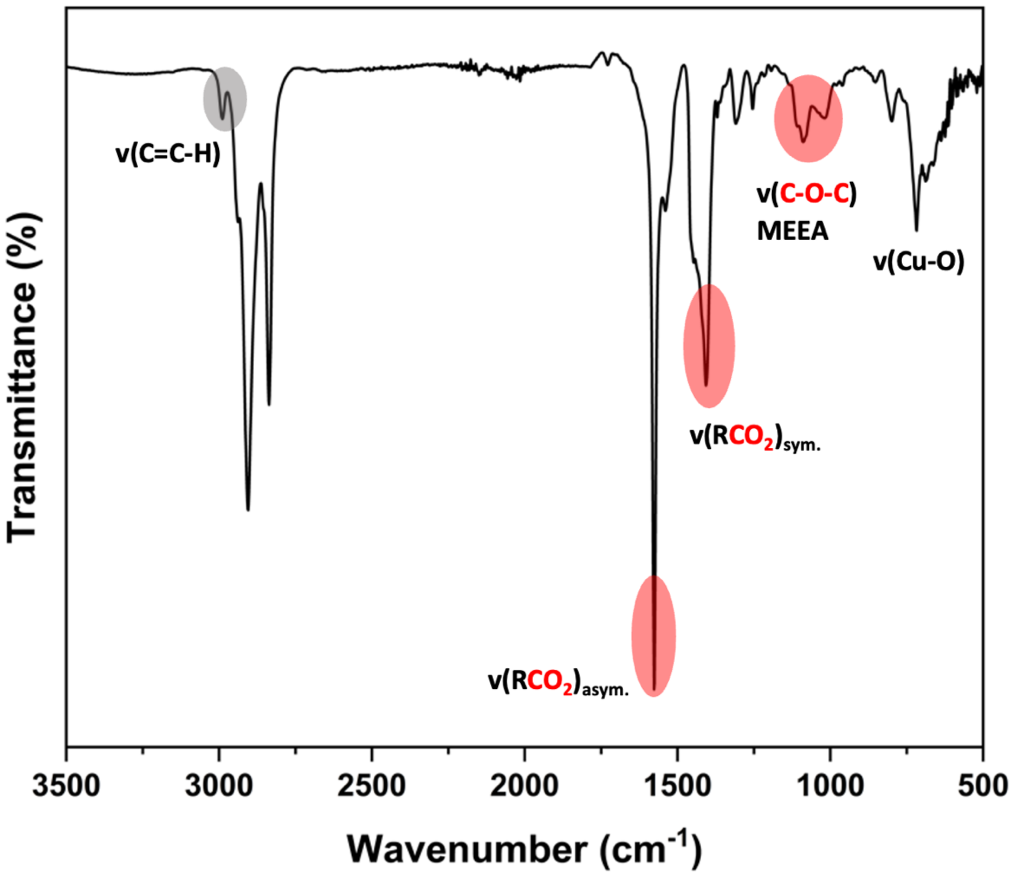

Figure S68. FT-IR spectrum of **1-OCo(O_2CR^{ole})**. Key spectral features that indicate oleate coordination to Co are: (1) A stretch at 2990 cm^{-1} representing ν(C=C-H) of the internal alkene; (2) absence of a free ν(C=O) for oleic acid, indicating oleate incorporation as a carboxylate and not a carboxylic acid; and (3) asymmetric and symmetric carboxylate stretches indicating carboxylate coordination by MEEA and oleate. Stretches associated with -C_6F_5 are not observed (see Figures S16 and S17 for comparison).

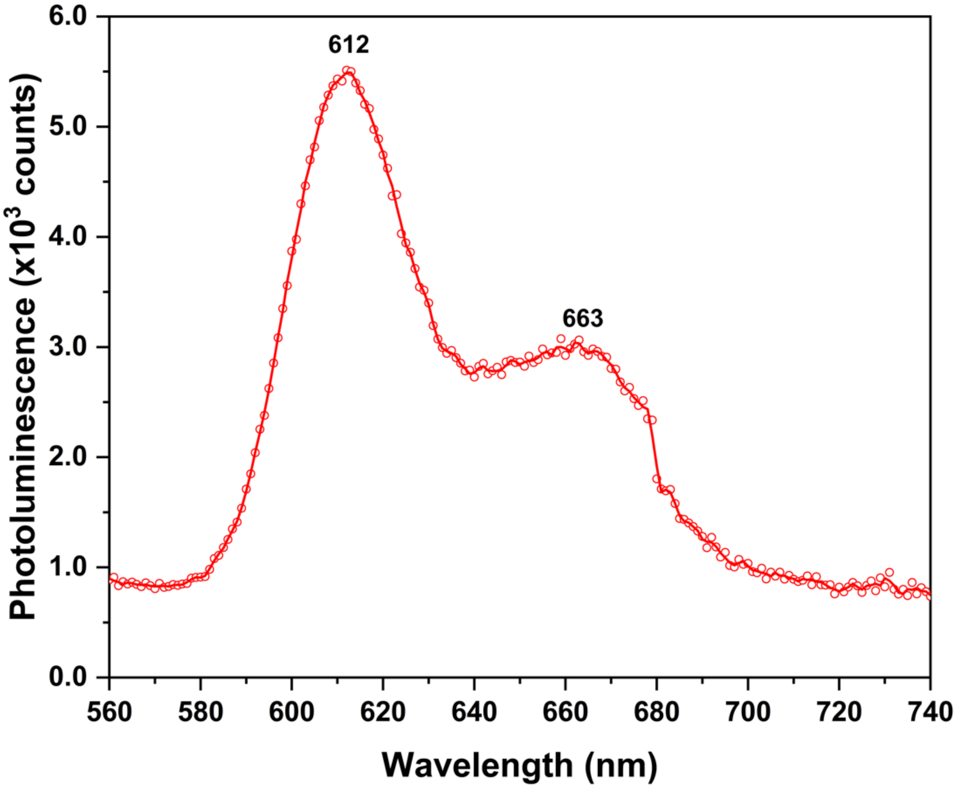

Figure S69. Photoluminescence spectrum of **1-OH** (0.18 mM in toluene). Experimental data (open circles) and fitted spectrum (smooth line) showing emission maxima at 612 and 663 nm.

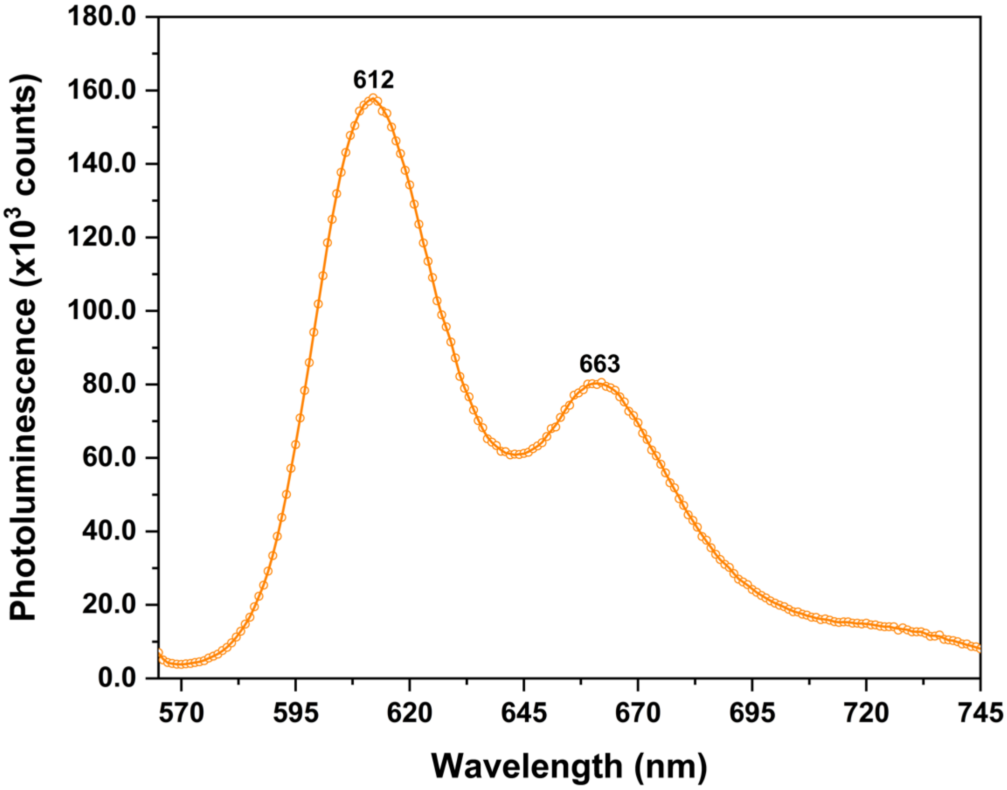

Figure S70. Photoluminescence spectrum of **1-OZn(O_2CR^{ole})** (0.18 mM in toluene). Experimental data (open circles) and fitted spectrum (smooth line) showing emission maxima at 612 and 663 nm.

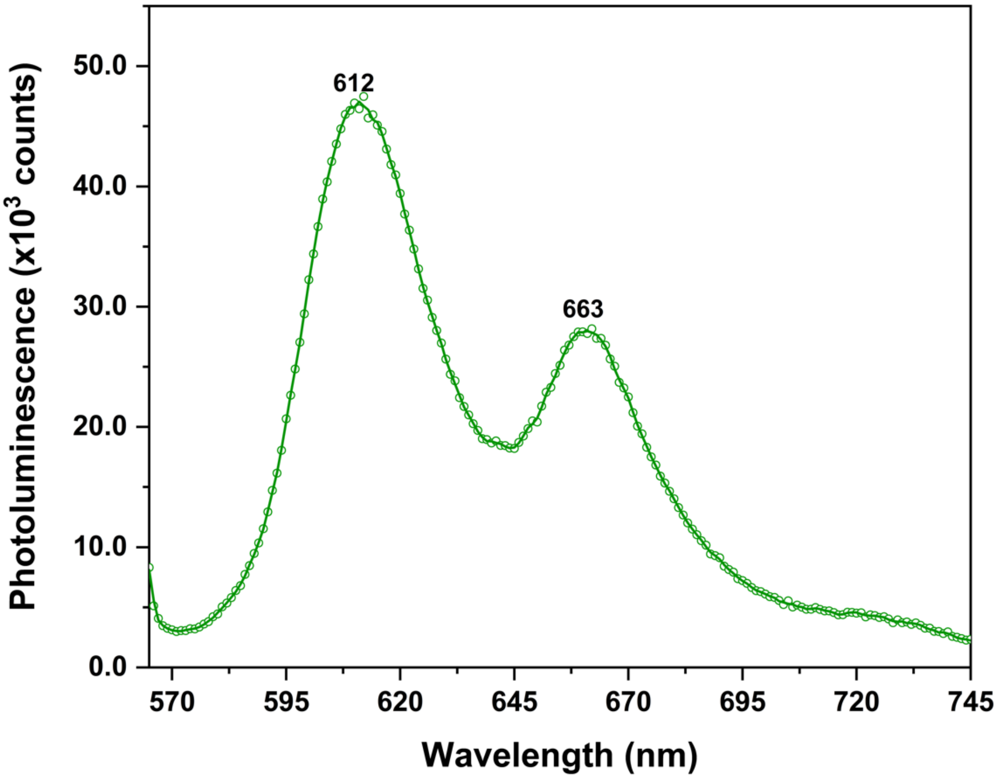

Figure S71. Photoluminescence spectrum of **1-OCo(O$_2$CR^{ole})** (0.18 mM in toluene). Experimental data (open circles) and fitted spectrum (smooth line) showing emission maxima at 612 and 663 nm.

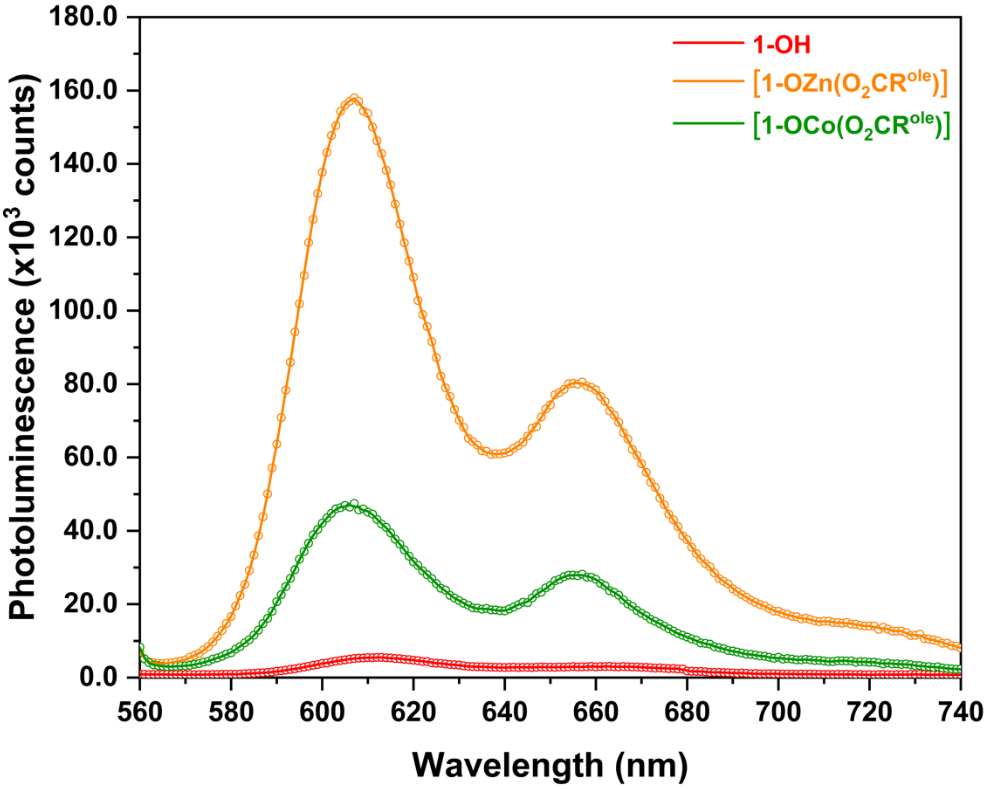

Figure S72. Stacked photoluminescence spectra of **1-OH**, **1-OZn(O$_2$CR^{ole})** and **1-OCo(O$_2$CR^{ole})**. In each case, the solution concentration is 0.18 mM in toluene. Experimental data (open circles) and fitted spectrum (smooth line).

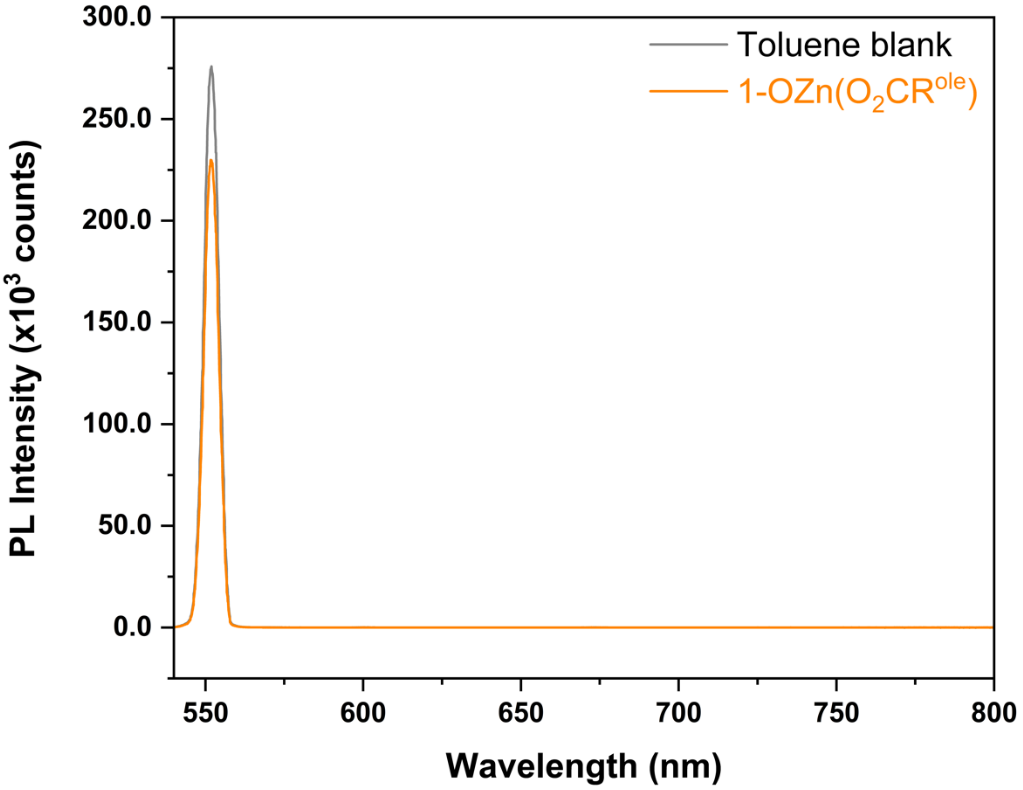

Figure S73. Signals of an integrating sphere measurement for **1-OZn(O_2CR^{ole})** (0.18 mM) and blank (toluene). No emission is observed past excitation.

References:

1. Burger, B. J.; Bercaw, J. E. Vacuum Line Techniques for Handling Air-Sensitive Organometallic Compounds in *Experimental Organometallic Chemistry-A Practicum in Synthesis and Characterization, Vol. 357*, American Chemical Society, Washington D.C., **1987**, p. 79.
2. Cowie, B. E.; Häfele, L.; Phanopoulos, A.; Said, S. A.; Lee, J. K.; Regoutz, A.; Shaffer, M. S. P.; Williams, C. K. Matched Ligands for Small, Stable Colloidal Nanoparticles of Copper, Cuprous Oxide and Cuprous Sulfide. *Chem. Eur. J.* **2023**, *29*, e202300228.
3. M. Wojdyr, *J. Appl. Cryst.* **2010**, *43*, 1126-1128.
4. Brown, N. J.; Weiner, J.; Hellgardt, K.; Shaffer, M. S. P.; Williams, C. K. Phosphinate Stabilised ZnO and Cu Colloidal Nanocatalysts for CO_2 Hydrogenation to Methanol. *Chem. Commun.* **2013**, *49*, 11074-11076.
5. Husbands, D. I.; Tallis, W.; Waldsax, J. C. R.; Woodings, C. R.; Jaycock, M. J. A Study of the Adsorption of Stearic Acid onto Ferric Oxide. *Powder Technol.* **1971**, *5*, 31-38.
6. Cooper, R. J.; Camp, P. J.; Henderson, D. K.; Lovatt, P. A.; Nation, D. A.; Richards, S.; Tasker, P. A. The Binding of Phosphonic Acids at Aluminium Oxide Surfaces and Correlation with Passivation of Aluminium Flake. *Dalton Trans.* **2007**, 1300-1308.
7. Jana, A.; Roesky, H. W.; Schulzke, C.; Samuel, P. P. Reaction of Tin(II) Hydride with Compounds Containing Aromatic C-F Bonds. *Organometallics* **2010**, *29*, 4837-4841.
8. Beil, S. B.; Möhle, S.; Enders, P.; Waldvogel, S. R. Electrochemical Instability of Highly Fluorinated Tetraphenyl Borates and Syntheses of Their Respective Biphenyls. *Chem. Commun.* **2018**, *54*, 6128-6131.
9. Pike, S. D.; White, E. R.; Regoutz, A.; Sammy, N.; Payne, D. J.; Williams, C. K.; Shaffer, M. S. P. Reversible Redox Cycling of Well-Defined, Ultrasmall Cu/Cu_2O Nanoparticles. *ACS Nano* **2017**, *11*, 2714-2723.
10. Chawla, S. K.; Sankarraman, N.; Payer, J. H. Diagnostic Spectra for XPS Analysis of Cu O S H Compounds. **1992**, *61*, 1-18.
11. Regoutz, A.; Kerherve, G.; Villar-Garcia, I.; Williams, C. K.; Payne, D. J. The Influence of Oxygen on the Surface Interaction Between CO_2 and Copper Studied by Ambient Pressure X-ray Photoelectron Spectroscopy. *Surf. Sci.* **2018**, *677*, 121-127.
12. Schön, G. ESCA Studies of Cu, Cu_2O and CuO. *Surf. Sci.* **1973**, *35*, 96-108.
13. van der Heide, P. A. W. Multiplet Splitting Patterns Exhibited by the First Row Transition Metal Oxides in X-ray Photoelectron Spectroscopy. *J. Electr. Spectr. Relat. Phenom.* **2008**, *164*, 8-18.
14. Galleni, L.; Sajjadian, F. S.; Conard, T.; Escudero, D.; Pourtois, G.; van Setten, M. J. Modeling X-ray Photoelectron Spectroscopy of Macromolecules Using *GW*. *J. Phys. Chem. Lett.* **2022**, *13*, 8666-8672.
15. Zhu, C.; Osherov, A.; Panzer, M. J. Surface Chemistry of Electrodeposited Cu_2O Films Studied by XPS. *Electrochimica Acta* **2013**, *111*, 771-778.
16. Nansé, G.; Papirer, E.; Fioux, P.; Moguet, F.; Tressaud, A. Fluorination of Carbon Blacks: An X-Ray Photoelectron Spectroscopy Study: I. A Literature Review of XPS Studies of Fluorinated Carbons. XPS Investigation of Some Reference Compounds. *Carbon* **1997**, *35*, 175-194.
17. Sun, L.; Peng, C.; Kong, L.; Li, Y.; Feng, W. Interface-Structure-Modulated CuF_2/CF_x Composites for High-Performance Lithium Primary Batteries. *Energy Environ. Mater.* **2022**, *0*, 1-9.
18. Löchel, B. P.; Strehblow, H.-H. Breakdown of Passivity of Nickel by Fluoride. *J. Electrochem. Soc.* **1984**, *131*, 713-723.
19. Deacy, A. C.; Kilpatrick, A. F. R.; Regoutz, A.; Williams, C. K. Understanding Metal Synergy in Heterodinuclear Catalysts for the Copolymerization of CO_2 and Epoxides. *Nat. Chem.* **2020**, *12*, 372-380.
20. Obtained from Scifinder; Calculated using Advanced Chemistry Development (ACD/Labs) Software V11.02 (© 1994-2023 ACD/Labs)
